# Local protein structure prediction using discriminative models

**DOI:** 10.1186/1471-2105-7-14

**Published:** 2006-01-11

**Authors:** Oliver Sander, Ingolf Sommer, Thomas Lengauer

**Affiliations:** 1 Max-Planck-Institute for Informatics, Department of Computational Biology and Applied Algorithmics, Stuhlsatzenhausweg 85, D-66123 Saarbrücken, Germany

## Abstract

**Background:**

In recent years protein structure prediction methods using local structure information have shown promising improvements. The quality of new fold predictions has risen significantly and in fold recognition incorporation of local structure predictions led to improvements in the accuracy of results.

We developed a local structure prediction method to be integrated into either fold recognition or new fold prediction methods. For each local sequence window of a protein sequence the method predicts probability estimates for the sequence to attain particular local structures from a set of predefined local structure candidates.

The first step is to define a set of local structure representatives based on clustering recurrent local structures. In the second step a discriminative model is trained to predict the local structure representative given local sequence information.

**Results:**

The step of clustering local structures yields an average RMSD quantization error of 1.19 Å for 27 structural representatives (for a fragment length of 7 residues). In the prediction step the area under the ROC curve for detection of the 27 classes ranges from 0.68 to 0.88.

**Conclusion:**

The described method yields probability estimates for local protein structure candidates, giving signals for all kinds of local structure. These local structure predictions can be incorporated either into fold recognition algorithms to improve alignment quality and the overall prediction accuracy or into new fold prediction methods.

## Background

In recent years progress has been made in protein structure prediction by incorporating information on local protein structure. David Baker's group has successfully used local fragment predictions [1-4] in conjunction with a fragment assembly procedure to substantially improve new fold predictions [5-7]. Also for fold recognition and remote homology detection methods the integration of local fragment predictions led to improved results [8].

Methods for analyzing fragments focus on sequence or structure or both. We are looking for fragments that occur in several proteins, that are sufficiently similar in structure, and that exhibit enough sequence similarity to be detectable by discriminative methods.

Specifically, we address the question: Given a local sequence fragment, how much can we learn about the local structure it adopts? It is expected that, in many cases, knowledge of the sequence of the fragment is not enough to determine its structure. Often long-range interactions with parts of the amino acid chain that are far away in sequence but close in space can be determinants for local structure. However, in other cases the local sequence properties give rise to a single or just a few local structures. Knowledge of these conserved cases enables the prediction of local structure in the respective regions of the protein.

In the following we briefly review two approaches to analyzing protein fragments (one by Baker's group and one by Hunter and Subramaniam) and compare them with our own method.

**Figure 1:**
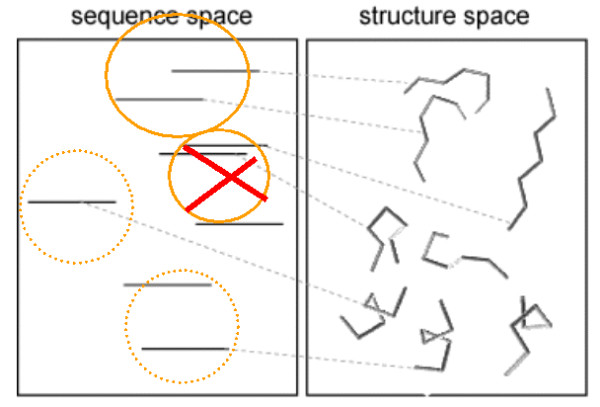
Clustering in sequence space and discarding clusters with too much structural variation (Baker's Method).

Baker's approach [1,3] starts by clustering fragments based on sequence similarity. In the second step for each of the clusters the structural variation of the fragments is examined. Clusters that vary too much are discarded. The remaining clusters represent sequence neighborhoods that adopt only one or few local structures (see Figure 1).

**Figure 2:**
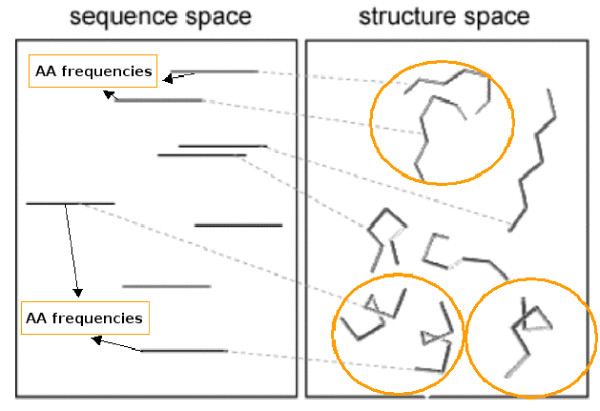
Clustering in structure space and generating sequence profiles for each structural cluster for Naive Bayes classification (Hunter's Method).

Hunter's approach [9] clusters fragments based on structural similarity in order to define 28 canonical local structures. These are supposed to roughly model local structural variation. After tabulating the corresponding sequences of each of the classes and calculating position-specific amino-acid probabilities, a simple prediction algorithm (Naive Bayes) is used to predict the canonical fragment structure based on sequence information (see Figure 2).

**Figure 3:**
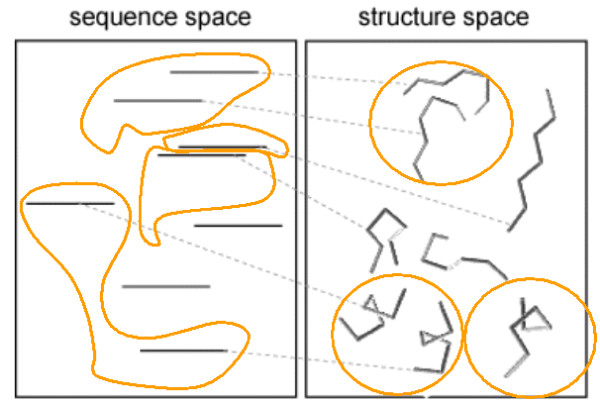
Clustering in structure space and using a nonlinear classifier for partitioning sequence space with respect to corresponding structure labels (Our Method).

Our approach (Figure 3) combines the advantages of both ideas. Baker's algorithm partitions sequence space regardless of the corresponding structures. As sequences vary to a greater extent than structures, incorporating structural information while partitioning sequence space is crucial. This idea can be realized using a classifier, which partitions sequence space into decision regions, such that inference of local structure is optimized. In place of Hunter's simple Naive Bayes approach, we suggest a more flexible classifier like a support vector machine or a random forests to map from local sequence to structure. Here, the question of the partitioning granularity of sequence space amounts to the classifier regularization problem, which can be addressed by commonplace validation methods. Moreover, our method of defining recurrent local structures is different from Hunter's approach and exhibits improved performance regarding Hunter's quality criteria.

Early work on local structural fragments was done by Rooman et *al*. [10], who use a hierarchical procedure to cluster 7-residue fragments into a coarse grouping (4 clusters), which was shown to be similar to DSSP classes. Fetrow [11] uses a neural net to produce a low-dimensional feature representation for a distance- and angle-based description of local structure. Using *k*-means, this reduced feature representation is clustered into 6 groups, which show significant amino acid sequence patterns. Camproux et *al*. [12] use the hidden states in an HMM to derive a 12-letter alphabet of fragments which considers the distances within and the volume spanned by the fragment as well as consecutiveness of these structural building blocks. De Brevern et *al*. [13] propose a 16-letter alphabet generated by a self-organzing map based on a dihedral angle similarity measure. Similarly to Camproux's work, the chaining of consecutive fragments is considered. Using a Bayesian approach local structure is predicted based on local sequence (allowing a fuzzy n-to-m relationship between local structure and sequence). Recently the predictive performance on this alphabet was improved by Etchebest et *al*. [14]. A comprehensive evaluation of these and other structural alphabets was done by Karchin et *al*. [15,16].

## Results

To validate our methods we examine the quality of the structural clustering procedure, the accuracy of the classification into the resulting structural classes and the accuracy of the probability estimates in the classification step.

### Local structure clustering

We chose C_*α *_distance matrix comparison for clustering structure fragments, as it provides a vector space representation for structural similarity and is thus applicable to large data sets (see Methods for details). In order to estimate the suitability of this representation, we examined its correlation with the widely used root mean square deviation (RMSD). Scatter plots depicting strongly positive correlation and a Pearson correlation coefficient of 0.7962 indicate that distance matrix comparison is a reasonable representation of structural similarity. Although there are refined versions of distance matrix comparison, for instance by downweighting large distances, here we used the basic version.

A tradeoff exists between accentuating a natural clustering tendency, the number of clusters (resulting in a finer or coarser structure representation), and the complexity of the subsequent classification task (more defined classes are harder to predict accurately). Finally we decided on a threshold based on the following considerations: (1) There is no clear natural clustering as shown in the next section. (2) Posing a reasonable task for the subsequent classification the number of clusters should not be too large. (3) A comparison with the results of Hunter et *al*. [9] (whose method is the closest to our approach) requires a similar setting regarding modeling quality.

**Figure 4:**
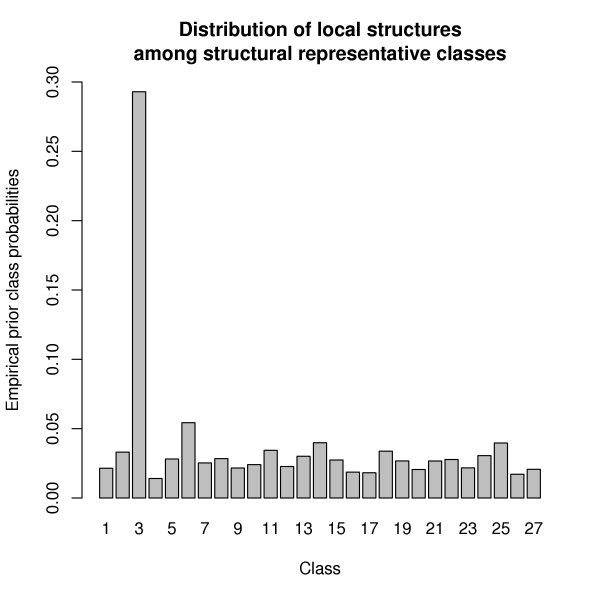
Prior probabilities of the structural classes. The helical fragments (class 3) are clearly dominating the other classes. Extended beta structures are split up into several smaller classes, reflecting their higher structural variability compared to helices.

Clustering with a threshold of 720 for the C_*α *_distance matrix comparison we obtain 27 clusters on the whole dataset (295,411 fragments). As can be seen in Figure 4, the distribution over the 27 classes is highly skewed. One class containing about 29% of the fragments dominates the other classes which contain between 1% and 5% of the fragments. As expected, the dominant class contains alpha helical fragments. Beta strand fragments are distributed among several structural classes. Their geometrical variation is almost as high as in loop regions, as is to be expected [17].

The structural clustering in [18] results in two dominant classes, one for alpha, and one for beta fragments. In terms of quantization error our clustering is superior, indicating that a subpartitioning of beta fragments is advisable. However, because of this different class balancing the classification results are not comparable. With two dominant clusters for alpha and beta fragments, secondary structure prediction would suffice to produce high classification accuracy.

#### Quality of the structural clustering

The validation of clustering results aims at quantifying to which extent the clustering resembles the natural grouping of data points. Many clustering algorithms require the specification of parameters, e.g. the number of clusters for *k*-means or a linkage threshold for hierarchical agglomerative clustering algorithms. Quality measures are used to adjust these parameters in order to cluster the data points appropriately.

In our clustering procedure we specify a threshold which controls the binning of fragments in the leader clustering algorithm. This parameter indirectly controls the number of clusters for the subsequent *k*-means refinement.

**Figure 5:**
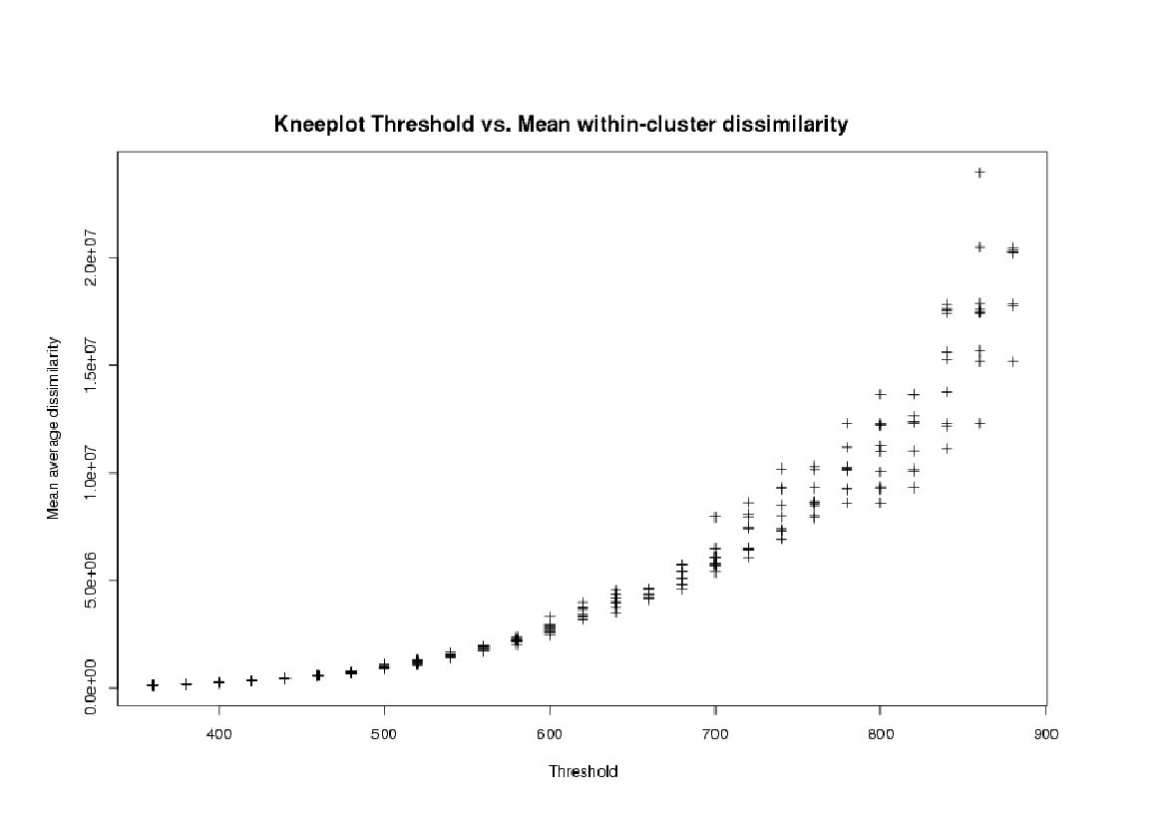
Kneeplot of the mean average dissimilarity across varying thresholds for the leader clustering algorithm. The mean average dissimilarity is computed as 1K∑k=1K1|Ck|∑i∈Ck∑i'∈Ckdi,i'
 MathType@MTEF@5@5@+=feaafiart1ev1aaatCvAUfKttLearuWrP9MDH5MBPbIqV92AaeXatLxBI9gBaebbnrfifHhDYfgasaacH8akY=wiFfYdH8Gipec8Eeeu0xXdbba9frFj0=OqFfea0dXdd9vqai=hGuQ8kuc9pgc9s8qqaq=dirpe0xb9q8qiLsFr0=vr0=vr0dc8meaabaqaciaacaGaaeqabaqabeGadaaakeaadaWcaaqaaiabigdaXaqaaiabdUealbaadaaeWaqaamaalaaabaGaeGymaedabaWaaqWaaeaacqWGdbWqdaWgaaWcbaGaem4AaSgabeaaaOGaay5bSlaawIa7aaaaaSqaaiabdUgaRjabg2da9iabigdaXaqaaiabdUealbqdcqGHris5aOWaaabmaeaadaaeWaqaaiabdsgaKnaaBaaaleaacqWGPbqAcqGGSaalcqWGPbqAcqGGNaWjaeqaaaqaaiabdMgaPjabcEcaNiabgIGiolabdoeadnaaBaaameaacqWGRbWAaeqaaaWcbaaaniabggHiLdaaleaacqWGPbqAcqGHiiIZcqWGdbWqdaWgaaadbaGaem4AaSgabeaaaSqaaaqdcqGHris5aaaa@51D3@, with *C*_*k *_being the data points in cluster *k*, and *K *being the total number of clusters. As the result of the leader algorithm depends on the input order of the data, the clustering procedure is repeated ten times with shuffled input.

As a first quality criterion of the clustering we used the mean of the averaged pairwise within-cluster distances. The result of the leader algorithm depends on the input order of the data, thus we repeated the clustering procedure ten times with shuffled input. The resulting curves are smooth, exhibiting no natural cutoff (Figure 5). This resembles the observations of Hunter and Subramaniam [18].

As a second quality criterion of the clustering process we used the quantization error in terms of RMSD. Clustering can be regarded as a data grouping process by which we represent whole groups by single representatives (similar to vector quantization in a vector space). As representative of a structural cluster, we choose the fragment with the lowest sum of distances to all other fragments in the respective cluster.

The quantization error is defined as the average RMSD distance of all fragments in the data set to their respective representatives. For two reasons we use the root mean square deviation to compute the quantization error. First, the RMSD is easier to interpret as it is commonly used to compare protein structures. Second, this validation shows that the C_*α *_distance matrix measure, which we used for efficient clustering, is able to find structure representatives that are good in terms of RMSD.

For 27 clusters, our clustering results in a quantization error of 1.19 Å. Hunter and Subramaniam specify the quantization error of their method for 28 clusters as 1.71 Å [18]. The difference between these two figures is an indication for the superiority of the structural representatives produced by our clustering method.

In the supplementary material [see Additional file 1] we provide the position-specific propensities for the types of secondary structure (H, E, and C). Alpha helices are clearly represented by cluster 3. This includes a few fragments with transitions to coils at the ends. Fragments in cluster 11 also have a clear helical center whereas coil structures dominate towards the ends. Overall, extended *β*-elements and coils are rather mixed in the clustering. Clusters 5 and 21 show stretches of *β*-strands with coils at the beginnings and ends, respectively. Clusters 6 and 14 both exhibit a *β *tendency in the middle and more coils towards both ends.

The supplement also contains information on the structural variability in each cluster, visualized as pairwise RMSD and pairwise secondary structure dissimilarity plots. These plots show that most of the clusters could be further divided into smaller subgroups. This is consistent with the smoothness of the kneeplot in Figure 5.

#### Examples of structural clusters

**Figure 6:**
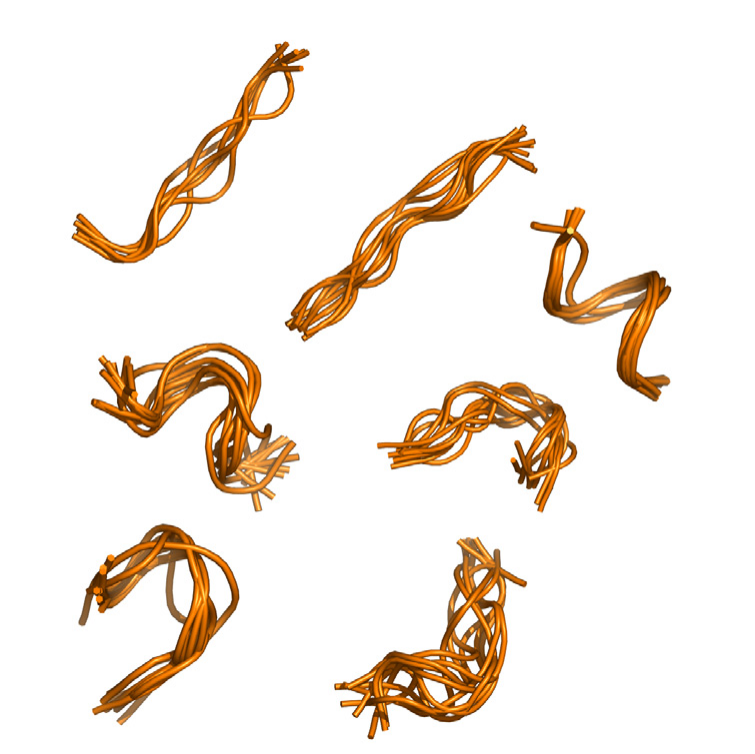
Selected examples of local structure clusters, including local structures like helices, beta strands, and beta strands merging into turns or loop regions. Each cluster is represented by ten randomly chosen fragments (superpositioned by Kearsley's quaternion method [19] and visualized in PyMOL [20]). Some of these clusters represent well defined local patterns, whereas others are only a rough grouping of similar structures.

To give an impression of the structural clusters, we show some examples in Figure 6. Random samples of ten fragments from clusters were taken, superpositioned by Kearsley's quaternion method [19] and visualized in PyMOL [20]. Note that the sample is not necessarily representative. From the resulting visualizations, we chose a few that demonstrate interesting local structures like helices, beta strands, beta strands attached to turns or loop regions. Whereas some of these clusters represent well defined local patterns, others are only a coarse grouping of similar structures.

### Classification

In this section we describe experimental results of the sequence to structure mapping by a discriminative classifier. Knowing that local sequence plays only a partial role in the formation of local structure, it is obvious that the mapping is a difficult task, when totally neglecting tertiary relationships.

Examining the correlation between sequence similarity (Log-average scores [21], Manhattan distance, and Euclidean distance of sequence profiles) and structural similarity (RMSD) for local windows of 7 residues in length shows negligible correlation of -0.0479, 0.0186, and 0.0398 respectively.

All of the following observations refer to fragments with a length of 7 residues. The structure labels obtained from the previous step are for a fixed clustering threshold of 720, which leads to 27 class labels.

First we show results for classification without confidence estimates, with focus on support vector machines as the predictive model. Then, results for the probability estimates are shown. The respective problematic SVM performance suggests a shift of focus towards random forests as classification model.

#### Prediction accuracy

We used decision trees (C5.0), support vector machines (SVM) and random forests (RF) to predict local structure classes given sequence information. The peak accuracies for C5.0, SVMs, and RFs are 0.2320, 0.3615, and 0.3409 respectively (see Table 1; for runtime restrictions not all combinations have been computed).

As a rather simple model, a decision tree was used to estimate the difficulty of the classification problem. Using C5.0 with standard parameters and input based on physico-chemical properties yields classification accuracy of 0.2320. This stresses the inherent complexity of the mapping task.

For SVMs the training complexity is quite high, thus exhaustive testing of all interesting parameter combinations is not possible. We used a simple grid-search procedure on promising parameter regions. By manual intervention these regions where extended into directions, in which further improvement was expected. For the relevant SVM parameters *C *(error penalty) and *γ *(RBF kernel parameter) we decided to use the parameter ranges proposed by Hsu et *al*. [22]. *C *ranges over {2, 4, 8, 16, 32, 64}, *γ *ranges over {0.03125, 0.0625, 0.125, 0.25, 0.5, 1, 2}.

Experimenting with different profile representations (see Methods), the encoding of amino acid properties exhibits clear advantages. For *C *= 2 and *γ *= 0.03125 the prediction accuracy rises from 0.3038 (for amino-acid profiles) to 0.3426 (for property-based profiles). The performance using only representations of single sequences is below that of using amino-acid profiles.

Changing the size of the training set from 5,000 to 20,000, the classification accuracy rises from 0.3426 to 0.3615. However, the run time is increased significantly to approximately one day for five-fold cross validation on 20,000 fragments (on a SUN Ultra SPARC-III+ with 900 MHz).

For random forests the overall accuracy is 0.3409 using the standard setting of 500 trees (for 20,000 training samples).

#### Accuracy of probability estimates

To achieve better confidence in the prediction step, for each classified sample a vector is returned that contains a probability for each of the classes. This probability is supposed to represent the confidence with which the sample can be assigned to the class. High probabilities represent high confidence.

To retrieve probability estimates from support vector machines predictions need to be postprocessed (see Methods). Predicting just the class with the highest probability, classification accuracy drops to 0.29 (compared to 0.34 if no probability estimates are used). Thus the postprocessing step significantly decreases predictive performance. During corresponding tests in [23] no performance loss was observed. In our case, due to the imbalance in the class sizes, probability estimates are biased towards the dominant class (the helical class in our case). This results in an over-representation of the dominant class at the top-rank.

**Figure 7:**
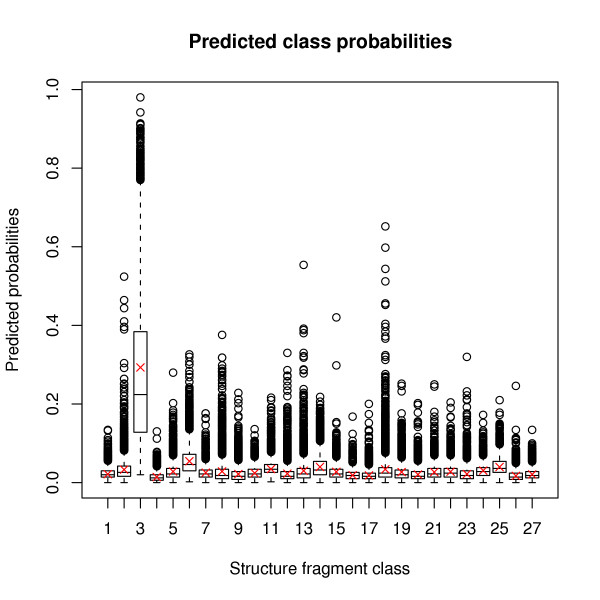
Boxplot of probability estimates (RF classifier). The boxes range from the first to the third quartile. The lines within the boxes denote the medians, the whiskers extend to the maximum and minimum values unless they are outliers, outliers are plotted as distinct circles. The red crosses indicate the prior class probabilities. (For presentation reasons, predictions for a subsample of the test set are shown).

Similarly to the boxplots for the class-wise probability estimates in Figure 7, for SVMs it can be observed as well that the ranges of the given estimates reflect class size (Figure 4). This also explains the poor performance of the SVM probability estimates. If the sensitivity towards the smaller classes were higher (higher probability estimates), the specificity would decrease dramatically, as many of the samples in the dominant class would be erroneously classified into one of the smaller classes. Thus by predicting conservative estimates for the small classes SVMs lose overall accuracy.

For random forests probabilistic outputs are a natural extension of the standard algorithm, yielding the same classification accuracy. Due to the low overall accuracies, probability estimates are mandatory, thus we decided to focus on using random forests.

**Figure 8:**
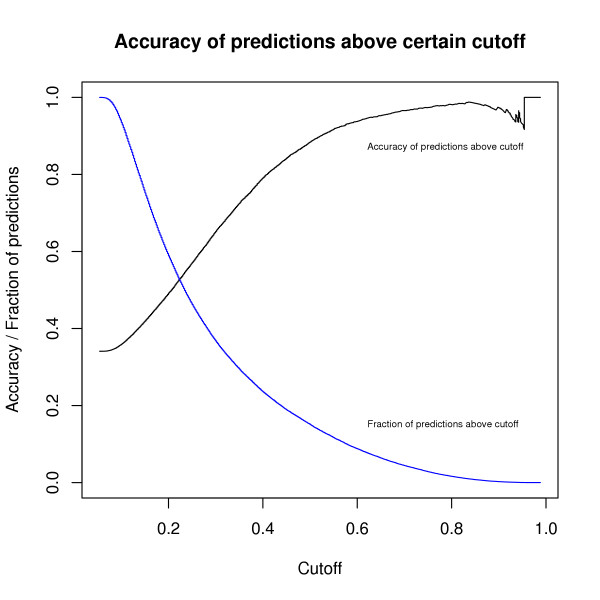
Classification accuracy, if all predictions below a given confidence threshold are discarded (RF classifier). High confidence parts of the plot mostly consist of predictions for the dominant class according to the predicted confidence ranges in figure 7.

Limiting predictions to high confidence decisions can increase classification accuracy. In Figure 8 we plot confidence thresholds against accuracy. However, interpreting the resulting plot can be misleading. Keeping the class specific ranges of confidence predictions from Figure 7 in mind, it becomes obvious, that large parts of the plot describe predictions for the dominant class.

**Figure 9:**
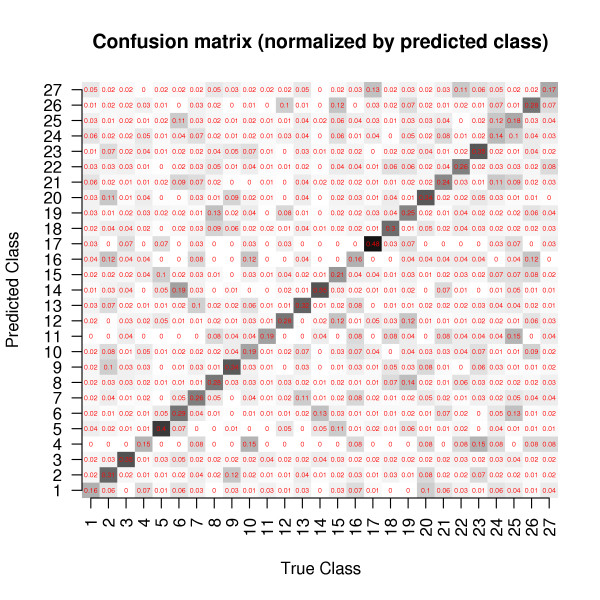
Prediction-conditioned confusion matrix (RF classifier).

To level out the effect of dominant classes, we evaluate prediction accuracy conditioned on the predicted class (see Figure 9). Thus given the classifier decision for a specific class, the real class is more likely to be the predicted one than another.

The confusion matrix shows some dominant mispredictions. Looking at these cases shows that the classes which are confused with each other, often exhibit similar secondary structures (see supplementary material). For example class 2 which is confused with class 9, both are coiled with a tendency to helix at the end. Likewise, cluster 4 which is often predicted instead of clusters 10 and 23 show a similar combination of coil and helix. Clusters 6, 14, and 25 have a conserved extended center with coils towards the ends. Clusters 8 and 19 are coiled with a helical tendency at the beginning and a bias towards extended structures at the end.

**Figure 10:**
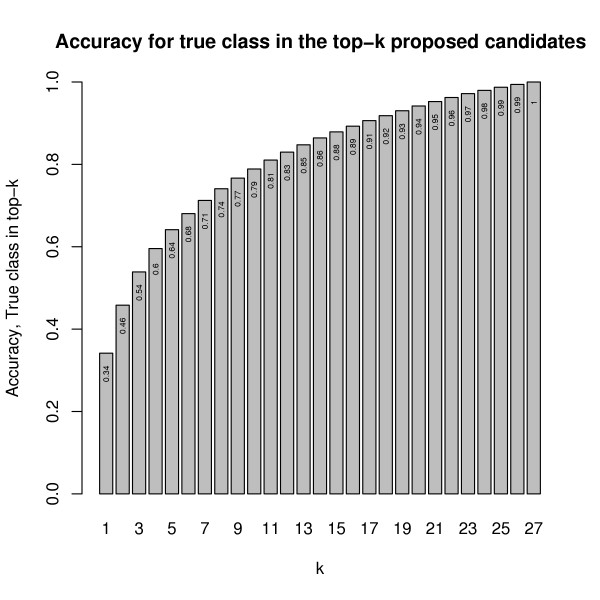
Correct classifications, if the top-*k *ranked predictions are used (20,000 samples, RF classifier).

Despite the difficulties resulting from the imbalance of class sizes, the probability estimates entail signals also for the occurrence of underrepresented classes. Using them to enrich local structure candidates for fragment assembly or incorporating them into an alignment score for fold recognition can improve prediction quality. In an enrichment prediction, we allow the classifier to return several class suggestions per input sample. In Figure 10 the classification accuracy is shown, if several class suggestions are allowed. With up to three class suggestions the prediction contains the correct class in more than 54% of the cases. In protein structure prediction based on fragment analysis this can reduce the search space significantly. If additional constraints are taken into account, e.g. smoothing over the predictions of overlapping fragments, the results can be improved further.

**Figure 11:**
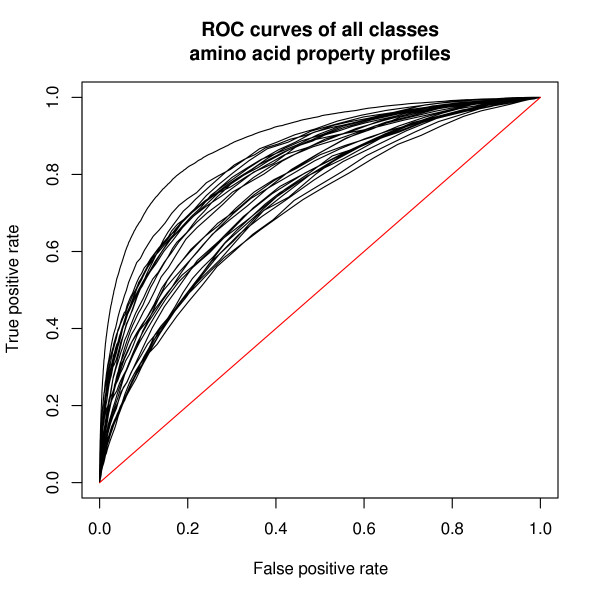
ROC curves for the 27 structural classes (using physico-chemical property profiles and RF classifier).

To evaluate the predicted probabilities of the single classes, without interfering effects of the class distribution, we generated receiver operating characteristics (ROC) plots of the results (see Figure 11). For an introduction to ROC plots see [24]. For classifiers with continuous output (e.g. confidence estimates), ROC graphs plot the false positive rate against the true positive rate. The false positive rate of a fixed class *i *is defined by FPR=false positivesnegatives
 MathType@MTEF@5@5@+=feaafiart1ev1aaatCvAUfKttLearuWrP9MDH5MBPbIqV92AaeXatLxBI9gBaebbnrfifHhDYfgasaacH8akY=wiFfYdH8Gipec8Eeeu0xXdbba9frFj0=OqFfea0dXdd9vqai=hGuQ8kuc9pgc9s8qqaq=dirpe0xb9q8qiLsFr0=vr0=vr0dc8meaabaqaciaacaGaaeqabaqabeGadaaakeaacqqGgbGrcqqGqbaucqqGsbGucqGH9aqpdaWcaaqaaiabbAgaMjabbggaHjabbYgaSjabbohaZjabbwgaLjabbccaGiabbchaWjabb+gaVjabbohaZjabbMgaPjabbsha0jabbMgaPjabbAha2jabbwgaLjabbohaZbqaaiabb6gaUjabbwgaLjabbEgaNjabbggaHjabbsha0jabbMgaPjabbAha2jabbwgaLjabbohaZbaaaaa@517D@. This is the number of samples erroneously classified in class *i *divided by the total number of samples not belonging to class *i*. The true positive rate is defined by TPR=true positivespositives
 MathType@MTEF@5@5@+=feaafiart1ev1aaatCvAUfKttLearuWrP9MDH5MBPbIqV92AaeXatLxBI9gBaebbnrfifHhDYfgasaacH8akY=wiFfYdH8Gipec8Eeeu0xXdbba9frFj0=OqFfea0dXdd9vqai=hGuQ8kuc9pgc9s8qqaq=dirpe0xb9q8qiLsFr0=vr0=vr0dc8meaabaqaciaacaGaaeqabaqabeGadaaakeaacqqGubavcqqGqbaucqqGsbGucqGH9aqpdaWcaaqaaiabbsha0jabbkhaYjabbwha1jabbwgaLjabbccaGiabbchaWjabb+gaVjabbohaZjabbMgaPjabbsha0jabbMgaPjabbAha2jabbwgaLjabbohaZbqaaiabbchaWjabb+gaVjabbohaZjabbMgaPjabbsha0jabbMgaPjabbAha2jabbwgaLjabbohaZbaaaaa@50BC@. This is the number of samples correctly classified in class *i *divided by the total number of samples in class *i*. The most important property of these rates is their independence from the class distribution. Random guessing would generate identical false positive and true positive rates, on average. Therefore, the diagonal (*y *= *x*) in the ROC plot is the performance of random guessing (shown as red line in our ROC plot).

**Figure 12:**
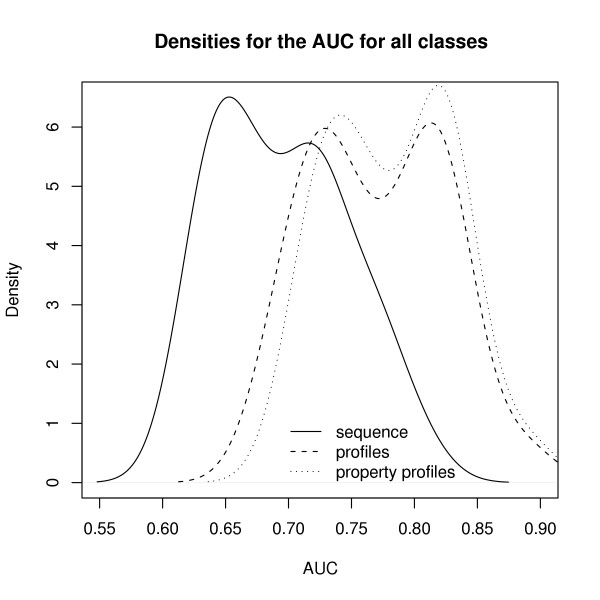
AUC densities for sequence only, amino acid profiles, and physico-chemical property profiles (RF classifier). Each of the density curves is generated from the area under the curve performance scores for each of the 27 classes.

In Figure 12 we compare the impact of different sequence representations on classification performance. For a single sequence representation the classification performance for each of the classes is computed as the area under the ROC curve (AUC). Thus, the 27 AUC measures can be illustrated as a density of AUCs. Comparing the density curves shows that input representation by frequency profiles performs better than input based on sequence alone. Furthermore, using amino acid property profiles slightly increases the performance compared to standard amino acid profiles.

**Figure 13:**
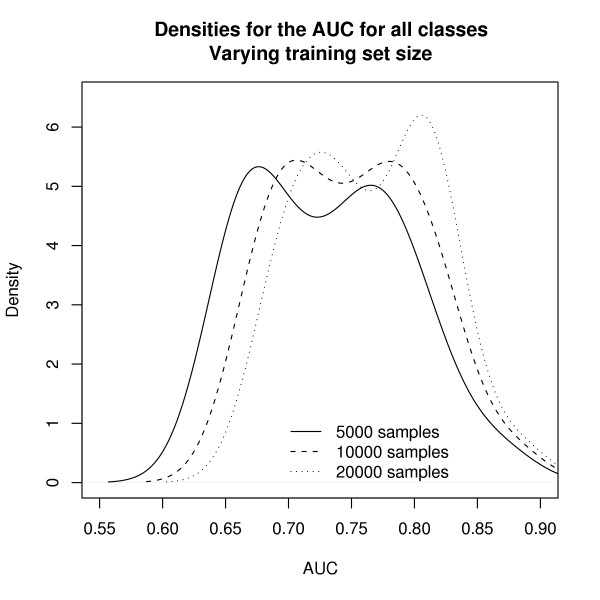
AUC densities for training set sizes 5,000 samples, 10,000 samples, and 20,000 samples (RF classifier). Each of the density curves is generated from the area under the curve performance scores for each of the 27 classes.

Figure 13 shows the effect of the size of the data set on classification performance. Varying the size of the data set from 5,000 over 10,000 to 20,000, the curves move towards the upper right corner, indicating rising performance. As training times are a major bottleneck we did not perform tests on larger data sets. Property-based profiles have more variables than amino-acid profiles (48·*k *compared to 20·*k *for *k *being the length of the fragment), training time is inherently longer.

### Comparability of classifier performance

Comparing the performance of the classification procedure to other methods (e.g. the methods by Baker and Hunter) is difficult. Obstacles for an objective comparison are (1) different prediction protocols (e.g. Baker only predicts in cases of high confidence) and (2) different representation of local structure space leading to a different prior distribution of class labels.

The final prediction rate equals to 36% that is less than the 44% of Hunter's method. One of the reviewers pointed out, that this seemingly lower rate is in fact better because the latter method predicts most local protein structures to be in only 10 of the 28 clusters and also tends to over-predict *β*-structures.

In order to compare our results to Hunter we adjusted our clustering manually, grouping together previously separated extended beta structures. Aside from the large *α*-helical class this leads to a second large class. Performing the classifier analysis with this modified fragment labeling leads to an increased classification accuracy of 44%. This shows that simple performance measures like accuracy are not able to capture the classification result. Ultimately the performance of local structure suggestions should be valued by their usefulness in later stages, e.g. their contribution to fragment assembly approaches.

### A note on fragments of various lengths

In our experiments above we limited the fragment size to seven residues. Hunter and Subramaniam use fragments of length seven, as structural variation of 7-residue fragments can be modeled at accuracies below 1 Å using fewer than 1,000 canonical local shapes. Moreover, current structure databases do not provide enough data for accurate modeling of longer fragments [18]. Bystroff and Baker note that the correlation between sequence and structure increases as the fragment length increases from three to eight, but slowly decreases for longer fragments [3].

Applying our method to fragments longer than 12 residues fails due to the strong increase in structural variation. The clustering procedure that we used requires a structure similarity threshold, specifying a limit of tolerated dissimilarity within a cluster. After setting these thresholds for longer fragments based on visual inspection of fragment pairs, the number of obtained clusters grew dramatically. Many of the clusters contain just one or a few fragments, which have no close neighbors in structure space. The complexity of the leader clustering algorithm is linear in the number of clusters and in the number of data points. However, if the number of clusters grows on the order of the number of samples, this amounts to quadratic complexity, rendering large-scale experiments infeasible. The approach by Hunter and Subramaniam is hampered by the same problem. Baker ignores this problem, as the number of clusters for sequence space partitioning is pre-specified, not considering the increasing structural variation with growing fragment length. Therefore, longer recurring fragments are implicitly discarded, unless they are represented super-proportionally to form a well conserved cluster.

In order to analyze the variation in structure space, we used a *k*d-tree to efficiently retrieve nearest neighbors of all data points. For length 7, only 11 data points had no neighbors closer than a given C_*α *_distance matrix score threshold (which was heuristically set based on visual inspection of fragment pairs, ranging from 420 for length 7 to 1,000 for length 11). For length 9 the number of "lonely data points" increased to 44, roughly 11,000 for length 10 and roughly 50,000 for length 11. However, as for longer fragments the usefulness of rigid comparison becomes questionable, flexible distance measures should be taken into account.

As the length of fragment pairs increases, the use of rigid structure comparison becomes questionable and techniques should be employed that take structural flexibility into account.

Several recent approaches use smaller fragments of 4, 5, or 6 residues [14,25,26]. While this reduces sequence-structure correlations, the advantage is that with a smaller set of representatives accurate modeling is possible. On the other hand chaining smaller fragments leaves more degrees of freedom in a fragment assembly approach. Ideally a fragment assembly procedure either uses long fragments for modeling conserved parts and small fragments to fill the gaps, or the context of small fragments is taken into account, e.g. by studying chains of consecutive fragments (e.g. [12,13]).

## Conclusion

We introduced a new approach to local protein structure prediction. In contrast to Baker's approach [3], we take into account structural information while partitioning sequence space. As sequence diversity is much higher than structural variation, it is expected that unsupervised learning in sequence space is harder than unsupervised learning in structure space. In this case the problem of choosing the correct granularity of the partitioning of sequence space takes the shape of the classifier regularization problem. Therefore, standard methods like cross-validation can be used to determine a partitioning with high predictive power. In contrast to Baker we can provide estimates of conservation even for less conserved fragments.

In contrast to Hunter's approach [9], we incorporate protein family information by using profiles instead of sequences. For secondary structure prediction this provision yielded a significant increase in performance [27]. The same could be observed in our case. The accuracy was further improved by using profiles based on amino-acid properties.

We see the main contributions of this work in (1) proposing a model for partitioning sequence space in dependence on the corresponding structures, (2) suggesting a representation of sequences through features based on amino-acid properties which can easily be used in classifiers and (3) introducing a distance metric for structural fragments which can be used in vector-space based methods.

There is room for further development of our method. The clustering procedure used for identifying recurrent local structure patterns is simple and can be enhanced in many ways. An important step is the removal of structural outliers. For increasing fragment sizes the number of expected outliers grows significantly. Procedures for iterative pattern expansion are an interesting approach to reducing the size of the search space for longer fragments. Also approximate nearest neighbor algorithms can be used to prefilter fragments. Another way of getting around the algorithmic restriction to smaller fragments is provided by methods working on sub-samples of the data set. We suggest that the quantization error quality criterion is a suitable measure for assessing the quality of the representative fragment set and thus can be used to evaluate further methodical improvements.

On the long run, there are numerous ways to push forward the understanding of local sequence structure relationships. Studying the evolutionary and physical role of protein fragments can lead to deep insights into the development of protein structures and function, as well as details of the folding process. Interesting questions include the automatic detection of candidates for evolutionarily conserved fragments [28], detection of patterns in the topology connecting smaller conserved fragments (e.g. [29] and integration of information about long-range interactions to discriminate between local structure ambiguities (e.g. [30]).

Some of these questions are so fundamental that finding a comprehensive answer and understanding their relationships might be of similar difficulty as the protein structure prediction problem itself.

## Methods

Our local structure prediction method predicts probability estimates for a local sequence to attain particular local structures from a set of predefined local structure candidates. The first step is to define a set of local structure representatives. In the second step a classifier is trained to predict the local structure representative given local sequence information.

### Datasets

We base our method development and validation on the nonredundant subset pdb_select25 [31] of the Protein Data Bank (PDB) [32]. In this subset all pairwise sequence similarities are below 25%. Representatives are chosen based on structure quality in terms of resolution and R-factor. Resolution and R-factor are guaranteed to be below 3.0 Å and 30%, respectively. The version of April 2003 contains 1999 protein chains with 324,426 residues in total. The corresponding structures are retrieved from the PDB.

For sequence representation we use profiles from the HSSP database [33]. Profiles in the HSSP database were constructed for a given protein by aligning the sequence of the protein with homologous sequences and calculating an amino acid frequency profile. To extend the approach to new sequences not covered by the HSSP database, profiles for the new sequences had to be built with an approach similar to the method of HSSP, i.e. an iterative SWISS-PROT search [34].

By sliding a window of fixed length over proteins we obtain fragments consisting of a sequence profile fragment and the respective structural representation in Cartesian coordinates. Removing fragments with missing profiles in HSSP or missing coordinates in the PDB we obtain 295,411 fragments of length 7.

### Discretization of local structures

Local structure fragments are obtained by collecting overlapping windows with a length of 7 residues from a set of non-redundant proteins (pdb_select25). Tests showed that excluding test structures was not necessary, i.e. no overfitting occurred if the local structure candidates were defined using the full set of local structures. This could be expected, as the number of selected representative fragments (27) is very low compared to the total number of fragments (295,411).

Using a clustering method with a suitable structural distance measure the fragments are grouped into disjoint sets. Subsequently centroid structures of each of these clusters are regarded as structural representatives.

As clustering method we chose a combination of the leader algorithm and *k*-means clustering. The leader algorithm [35] is used to find an initial clustering. All fragments are traversed sequentially and assigned to clusters. If an object is similar enough to the founding object of an existing cluster it is assigned to that cluster, otherwise it becomes the founding object of a new cluster.

Afterwards *k*-means clustering is used for iterative refinement. Data points are assigned to their closest cluster centers and the cluster centers are recomputed as the means of the data points in the clusters. This procedure is repeated until convergence of the clusters.

The runtime efficiency of this approach makes it applicable to our data set (in contrast to hierarchical clustering algorithms, which exhibit quadratic runtimes).

The clustering procedure requires a vectorial representation of data points. The commonly used root-mean-square-deviation (RMSD) is not suitable, as it requires the pairwise superposition of structures before comparison (e.g. [19]).

Instead, we chose a structural distance measure based on C_*α*_-C_*α *_distance matrix comparison. The efficacy of this structural distance measure is affirmed by the high Pearson correlation of 0.7962 between this distance measure and the root mean square deviation (RMSD).

The C_*α*_-C_*α *_distance matrix is defined as

D=(dij)=(d11d12…d21……)
 MathType@MTEF@5@5@+=feaafiart1ev1aaatCvAUfKttLearuWrP9MDH5MBPbIqV92AaeXatLxBI9gBaebbnrfifHhDYfgasaacH8akY=wiFfYdH8Gipec8Eeeu0xXdbba9frFj0=OqFfea0dXdd9vqai=hGuQ8kuc9pgc9s8qqaq=dirpe0xb9q8qiLsFr0=vr0=vr0dc8meaabaqaciaacaGaaeqabaqabeGadaaakeaacqWGebarcqGH9aqpdaqadaqaaiabdsgaKnaaBaaaleaacqWGPbqAcqWGQbGAaeqaaaGccaGLOaGaayzkaaGaeyypa0ZaaeWaaeaafaqabeWadaaabaGaemizaq2aaSbaaSqaaiabigdaXiabigdaXaqabaaakeaacqWGKbazdaWgaaWcbaGaeGymaeJaeGOmaidabeaaaOqaaiablAcilbqaaiabdsgaKnaaBaaaleaacqaIYaGmcqaIXaqmaeqaaaGcbaGaeSOjGSeabaaabaGaeSOjGSeabaaabaaaaaGaayjkaiaawMcaaaaa@44D0@

where *d*_*ij *_is the Euclidean distance between residue *i *and residue *j *(their C_*α*_-atoms). To compare two distance matrices *A *and *B *of the same size *L *× *L *we use:

∑i=1L∑j=1L|dijA−dijB|
 MathType@MTEF@5@5@+=feaafiart1ev1aaatCvAUfKttLearuWrP9MDH5MBPbIqV92AaeXatLxBI9gBaebbnrfifHhDYfgasaacH8akY=wiFfYdH8Gipec8Eeeu0xXdbba9frFj0=OqFfea0dXdd9vqai=hGuQ8kuc9pgc9s8qqaq=dirpe0xb9q8qiLsFr0=vr0=vr0dc8meaabaqaciaacaGaaeqabaqabeGadaaakeaadaaeWbqaamaaqahabaWaaqWaaeaacqWGKbazdaqhaaWcbaGaemyAaKMaemOAaOgabaGaemyqaeeaaOGaeyOeI0Iaemizaq2aa0baaSqaaiabdMgaPjabdQgaQbqaaiabdkeacbaaaOGaay5bSlaawIa7aaWcbaGaemOAaOMaeyypa0JaeGymaedabaGaemitaWeaniabggHiLdaaleaacqWGPbqAcqGH9aqpcqaIXaqmaeaacqWGmbata0GaeyyeIuoaaaa@48BD@

Thus, we define the structural distance between two protein fragments of the same length as the sum over the absolute differences of corresponding entries in the C_*α*_-C_*α *_distance matrices of the two fragments. In this way, each fragment is represented by the vector containing all entries of the C_*α*_-C_*α *_distance matrix and the *L*_1 _norm is used to compare two fragments.

### Mapping from local sequence to local structure

The second step uses a classifier to map sequence information to structural representatives. The classifier is trained with sequence information based on fragments spanning seven residues and on the corresponding structure labels obtained in the previous clustering step. All predictions were evaluated in a 5-fold cross-validation, i.e. in five iterations four fifth of the whole data set were available as training data, the remaining fifth was used as test data. Thus, each sample in the data set is used once for testing a model that was trained without the fold containing this sample. When the number of training samples was restricted for the evaluation, a randomly drawn subsample of the available training set per fold was used; testing was still performed on the whole data set as described above.

For the representation of local sequence information (a window of seven residues), we considered three different approaches. First, we used a categorical encoding of the amino acid sequences. This can be regarded as a degenerate case of amino acid profiles, in which at each sequence position exactly one amino acid occurs with relative frequency 1. Second, we used standard sequence profiles from the HSSP database [33], which were constructed from multiple alignments of homologous proteins. Thus the sequence description consists of a 20 × 7 matrix.

A third approach uses profiles of amino acid properties (e.g. polarity, hydrophobicity, size). Thus, a position in the property profile represents relative frequencies of 48 physico-chemical properties [36] instead of relative amino acid frequencies. Using a table of properties for amino-acids the sequence profiles from HSSP can be mapped to the according property profiles. The resulting relative frequencies do not sum to 1, as the physico-chemical properties are not mutually exclusive.

As classifiers we used decision trees (C5.0) as a simple classification scheme with possibly interpretable output, as well as support vector machines (SVM) and random forests (RF) as more sophisticated classifiers.

For decision trees [37] we used the commercially available program C5.0 by Quinlan [38]. As decision trees exhibited poor classification accuracy of 23.3% in initial tests, we did not include them in further experiments.

Support vector machines (SVM) are a statistical learning method, that can be trained to separate labelled data points into their respective classes. A separating hyperplane that yields the best expected separation on new data is fitted. By using an implicit transformation into a high-dimensional feature space data sets which are not linearly separable can be tackled. For an introduction to SVMs see [39,40]. We used the support vector machine implementation libSVM by Chang and Lin [41]. To tackle the multiclass problem we used the one-against-one strategy as suggested in [42]. In cases of limited overall classification accuracy a measure of reliability is crucial. Therefore, the methods of Platt [43] and Wu [23] are used to obtain a vector of probability estimates for the local structures. However, our experiments have shown that transforming discrete predictions into probability estimates reduced the overall classification accuracy significantly. Another drawback is that computing probability estimates requires the fitting of a function mapping from hyperplane distance to probabilities. This can be compute-intensive if many training samples have to be used.

Random forests are classifiers that are constructed from a combination of decision trees. The single trees are trained on randomly drawn sample of the training data and the split variables during tree construction are chosen from a drawn subset of all variables. Averaging over these varying trees a better generalization is achieved compared to single decision trees. For details about random forests see [44]. Random forests have been reported to be competitive with support vector machines regarding classification performance [45]. A significant benefit for our application is that random forests naturally provide probability estimates for classes. An additional step which can potentially decrease performance as necessary for support vector machines is not needed. In our experiments we used the randomForest implementation by Liaw [46] for the statistical language R.

## Authors' contributions

IS, TL, and OS conceptualized the project. OS developed the software, performed the experiments and drafted the paper. OS and IS analyzed the experimental results. IS and TL contributed to writing the paper. All authors read and approved the final manuscript.

## Supplementary Material

Additional File 1 Supplementary material, Detailed analysis and description of the 27 local structure clusters. Click here for file

## Figures and Tables

**Table 1:** Prediction accuracies for decision trees (C5.0), SVMs, and Random Forests.

Classifier accuracy	Single Seq (5000 samples)	HSSP Profiles (5000 samples)	Property profiles (5000 samples)	Property profiles (20000 samples)
C5.0	-	0.2320	-	-
SVM	0.2955	0.3038	0.3426	0.3615
SVM (prob. estim.)	-	-	0.2900	-
Random Forest	0.3024	0.3001	0.3102	0.3409
