# Supplementary material for: Local protein structure prediction using discriminative models
Source: BMC Bioinformatics. 2006 Jan 11;7:14. doi: 10.1186/1471-2105-7-14 (PMC1368994; doi:10.1186/1471-2105-7-14)
Supplement: Additional File 1 — Supplementary material, Detailed analysis and description of the 27 local structure clusters. [file 1471-2105-7-14-S1.pdf]

Supplementary Material for
Local Protein Structure Prediction Using Discriminative Models

Oliver Sander[1,*], Ingolf Sommer[1], and Thomas Lengauer[1]
(1) Max-Planck-Institute for Informatics,
Department of Computational Biology and Applied Algorithmics,
Stuhlsatzenhausweg 85, D-66123 Saarbrücken, Germany

Contents

0 Introduction **3**

1 Structural variation in cluster number 1 **5**

2 Structural variation in cluster number 2 **8**

3 Structural variation in cluster number 3 **11**

4 Structural variation in cluster number 4 **14**

5 Structural variation in cluster number 5 **17**

6 Structural variation in cluster number 6 **20**

7 Structural variation in cluster number 7 **23**

8 Structural variation in cluster number 8 **26**

9 Structural variation in cluster number 9 **29**

10 Structural variation in cluster number 10 **32**

11 Structural variation in cluster number 11 **35**

12 Structural variation in cluster number 12 **38**

13 Structural variation in cluster number 13 **41**

14 Structural variation in cluster number 14 **44**

*Corresponding author: osander@mpi-sb.mpg.de

15 Structural variation in cluster number 15 **47**

16 Structural variation in cluster number 16 **50**

17 Structural variation in cluster number 17 **53**

18 Structural variation in cluster number 18 **56**

19 Structural variation in cluster number 19 **59**

20 Structural variation in cluster number 20 **62**

21 Structural variation in cluster number 21 **65**

22 Structural variation in cluster number 22 **68**

23 Structural variation in cluster number 23 **71**

24 Structural variation in cluster number 24 **74**

25 Structural variation in cluster number 25 **77**

26 Structural variation in cluster number 26 **80**

27 Structural variation in cluster number 27 **83**

0 Introduction

The following serves to illustrate the structural variability in the clusters. The clusters were derived on all 295 411 fragments as described in the manuscript. For each cluster three plots are given:

- The secondary structure content of each cluster is depicted by a position-specific secondary structure matrix. The relative frequencies for the secondary structure classes H, E, and C are listed for all residue positions in the 7-residue fragments.
- On a random sample of 100 fragments per cluster pairwise RMSD dissimilarities were computed and the fragments were subgrouped according to their pairwise dissimilarity. Some of the pairwise RMSD distances appear to be relatively high. Optimizing for good representatives for each cluster does not necessarily imply small pairwise RMSDs between all fragments in the clusters. This phenomenon is inherent to clustering tasks in high dimensional spaces, where clusters often constitute subspaces with substantial lower dimensionality.
- Similarly, on a random sample of 100 fragments per cluster pairwise secondary structure dissimilarities were computed and the fragments were subgrouped according to their pairwise dissimilarity.

Informal description of the 27 clusters

1	coil, some beta
2	coil, with some helical tendency (around 50 %) in the second half
3	clearly helical, slight coil tendency (below 20%) at the ends
4	coil, slight helical tendency at first 2 and coil/helical at last residue
5	two coil residues, followed by five beta
6	beta with some coil tendency towards both ends
7	coil, beta in the first half, stronger coil tendency in the second half
8	helix, coil in the first half, coil in the middle, coil, beta in the end
9	coil with some beta in the beginning and beta and helix in the end
10	coil and helical (almost 70 % coil in the middle)
11	helix and coil at the beginning and end, pronounced helical in the middle
12	coil with beta towards the end
13	coil/beta at the beginning, coil/helix at the end
14	clearly beta in the middle, beta/coil towards the ends
15	coil with beta towards the end
16	coil with some beta and helix towards the ends
17	coil/beta with coil dominance in the first half, beta dominance in the second half
18	helix at the beginning, coil at the end, also some beta (around 30 %) at beginning and end
19	pronounced coil with some helix at the beginning and beta at the end
20	coil with some beta at beginning and end and helix at the end
21	five mostly beta residues followed by two with strong coil tendency
22	coil/helix mixed at first four residues, rather coil towards the end
23	coil with some helix at beginning and end
24	balanced beta and coil
25	beta/coil with more beta in the middle, coil stronger at the beginning and end
26	coil with some beta and helix at beginning and end
27	coil with some beta and helix at beginning and end

1 Structural variation in cluster number 1

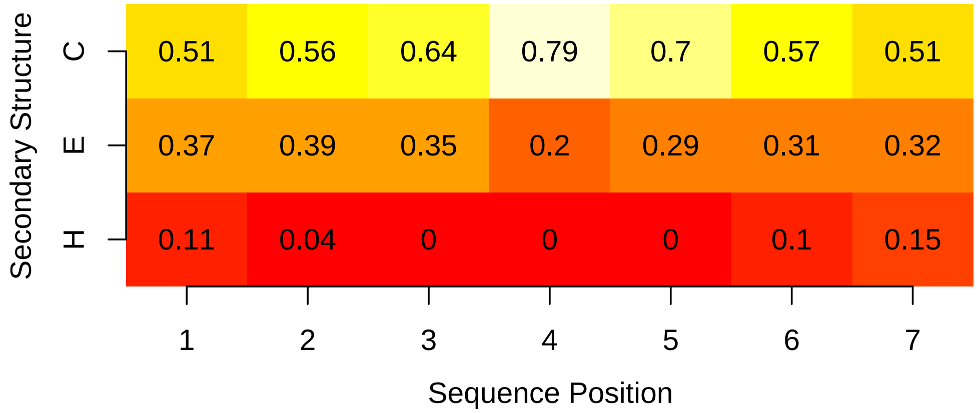

Figure 1: Position-specific secondary structure (STRIDE) in cluster 1.

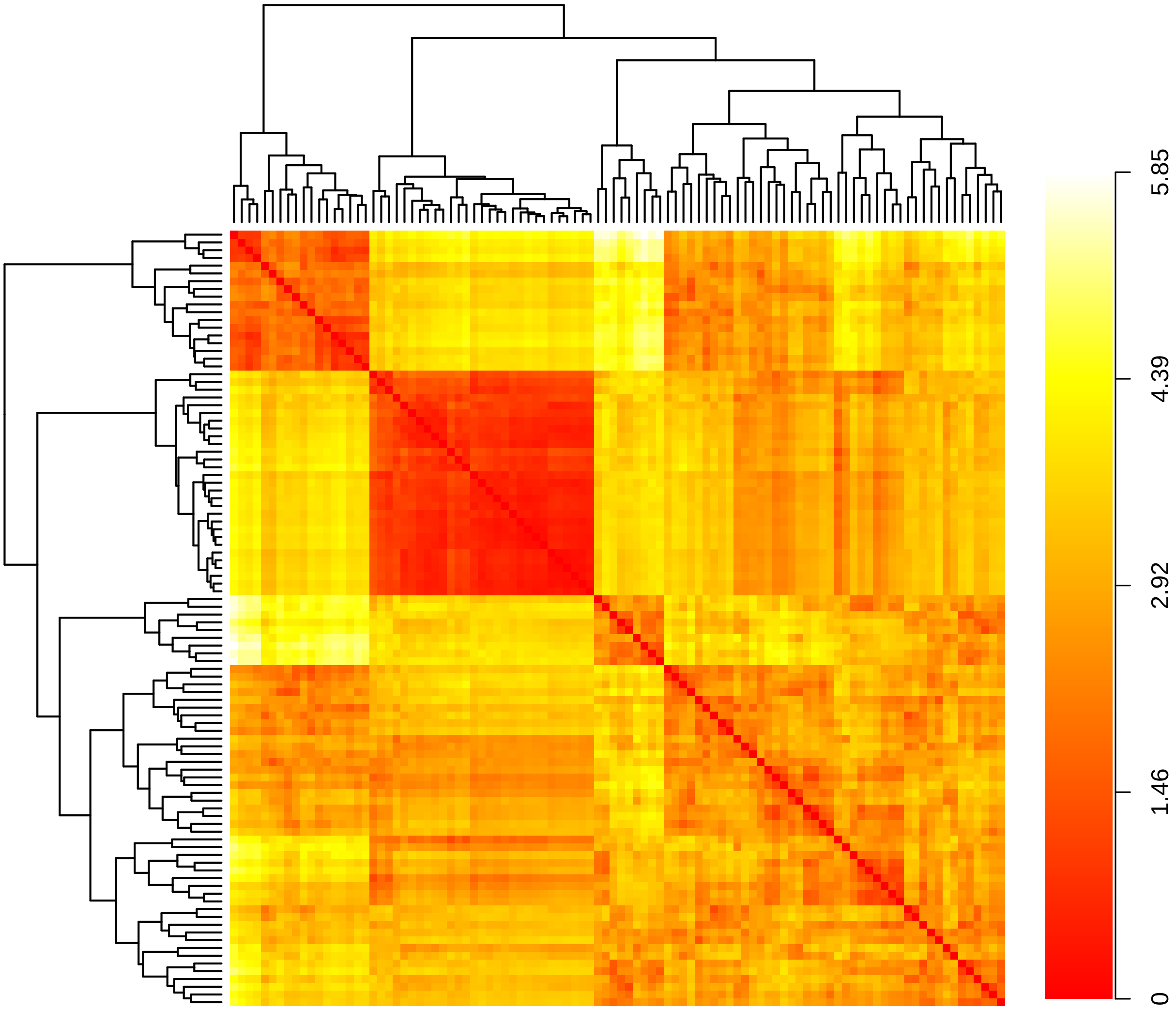

Figure 2: Pairwise RMSD in Cluster 1 (random sample of 100 fragments).

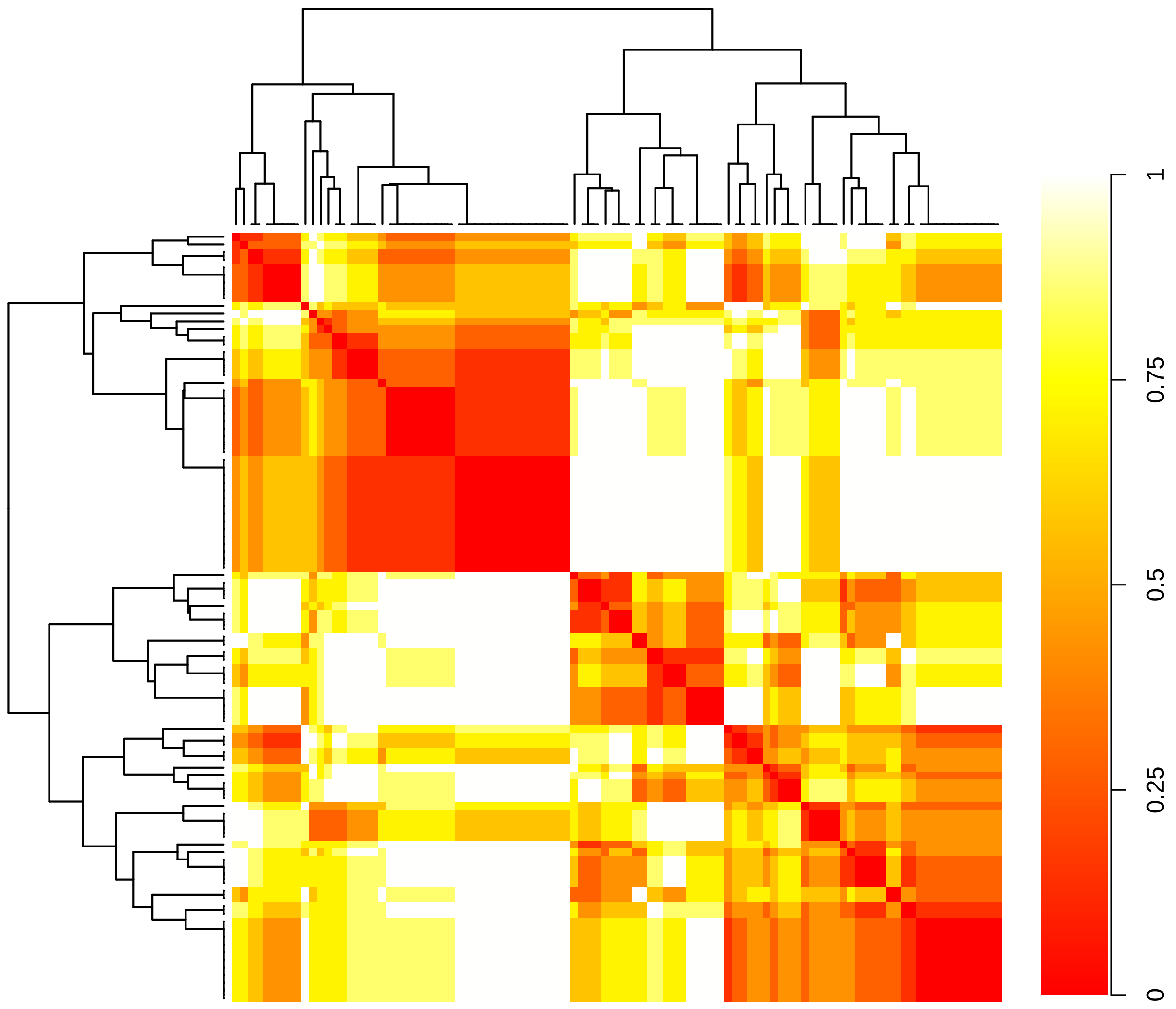

Figure 3: Pairwise secondary structure dissimilarity (1-Q3) in Cluster 1 (random sample of 100 fragments).

2 Structural variation in cluster number 2

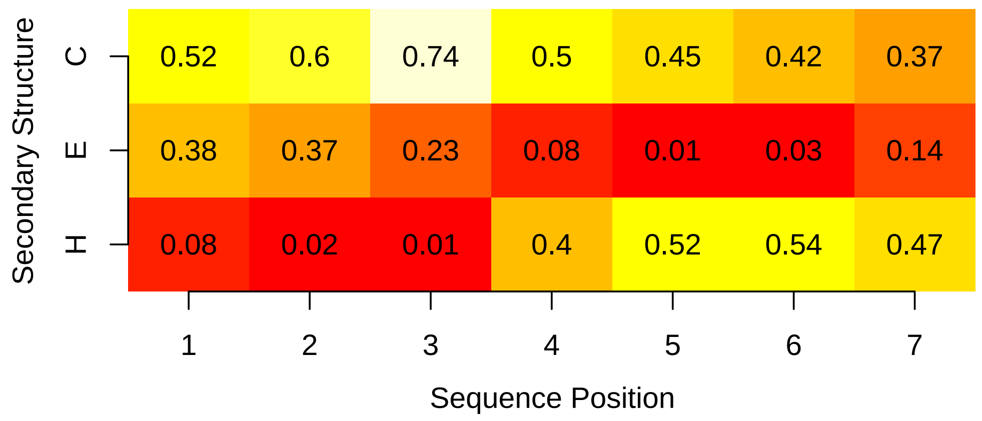

Figure 4: Position-specific secondary structure (STRIDE) in cluster 2.

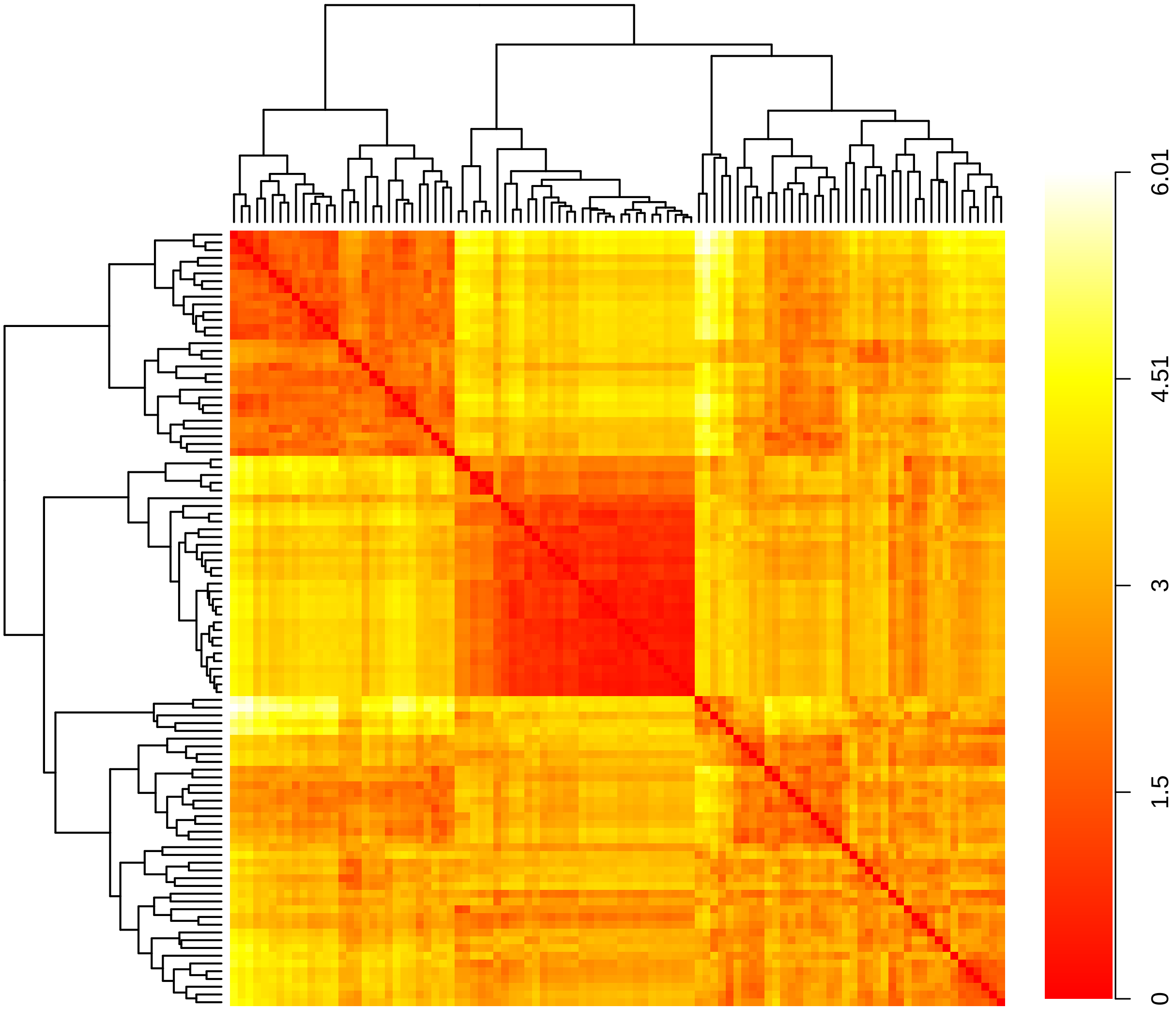

Figure 5: Pairwise RMSD in Cluster 2 (random sample of 100 fragments).

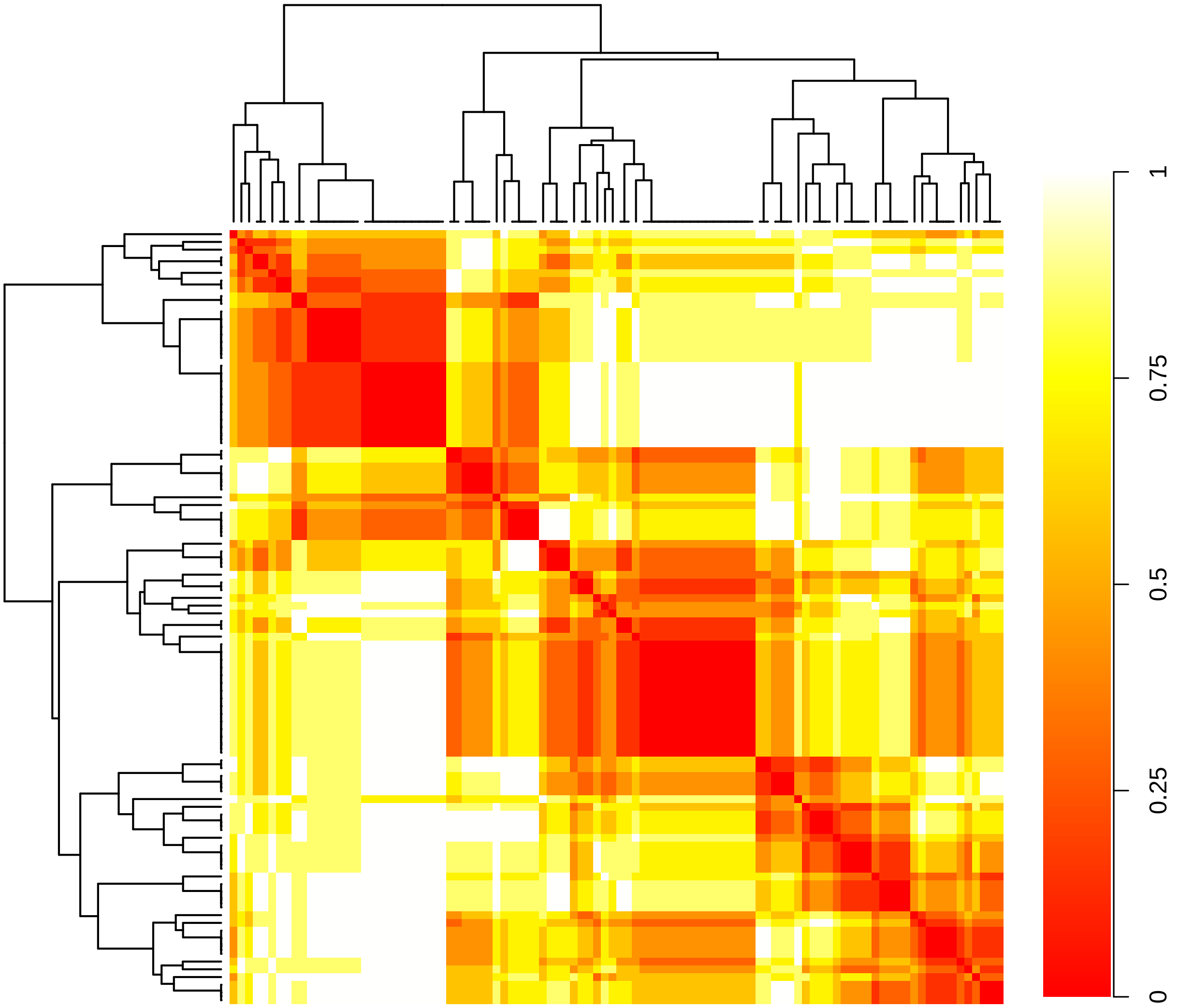

Figure 6: Pairwise secondary structure dissimilarity (1-Q3) in Cluster 2 (random sample of 100 fragments).

3 Structural variation in cluster number 3

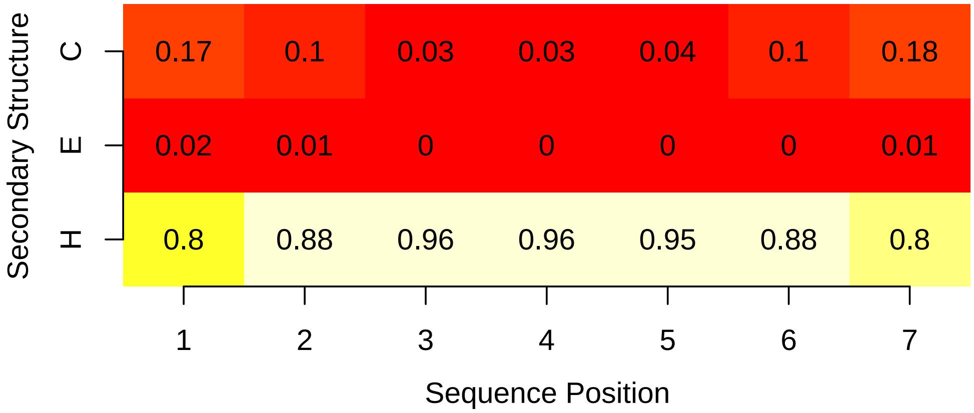

Figure 7: Position-specific secondary structure (STRIDE) in cluster 3.

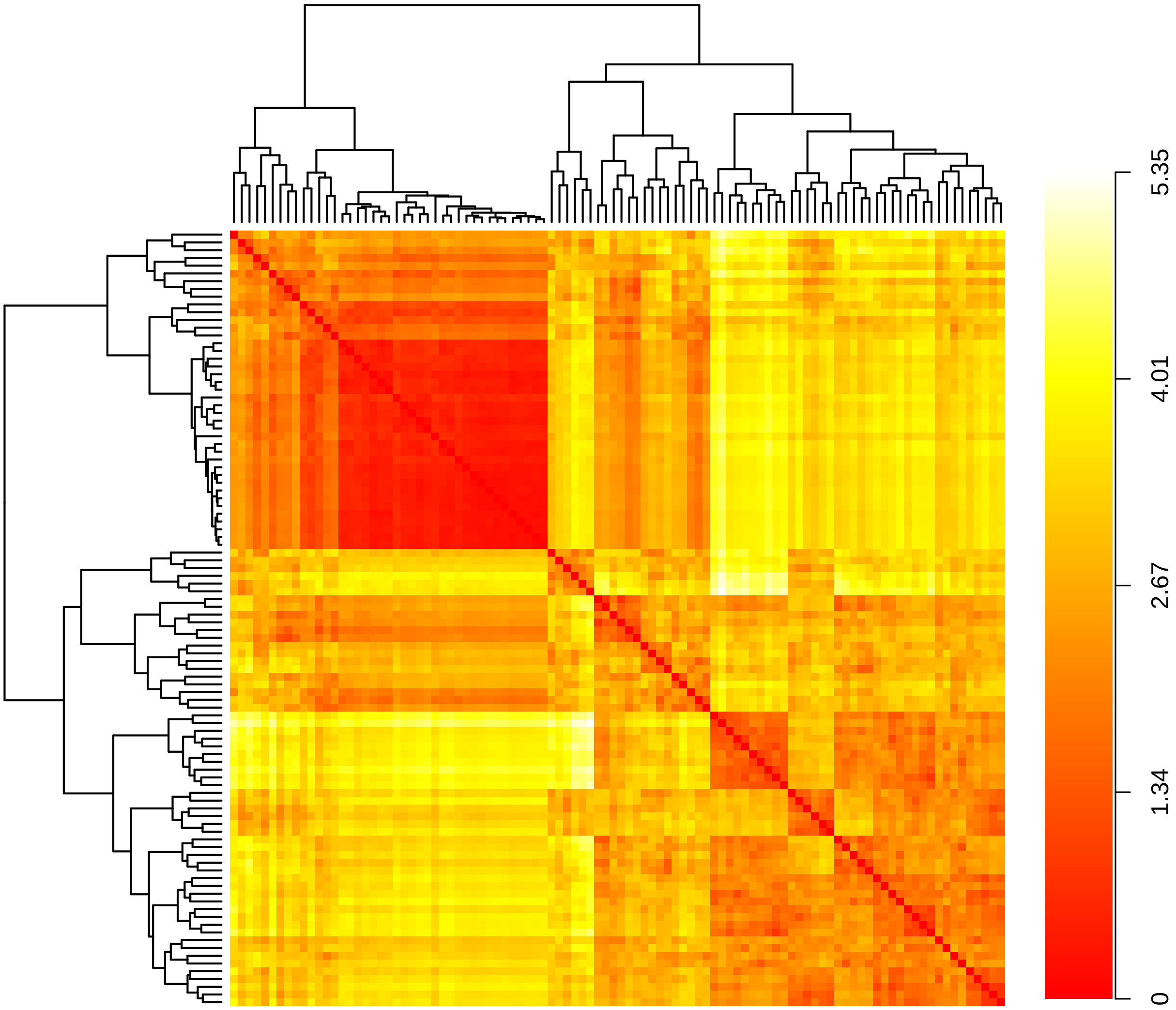

Figure 8: Pairwise RMSD in Cluster 3 (random sample of 100 fragments).

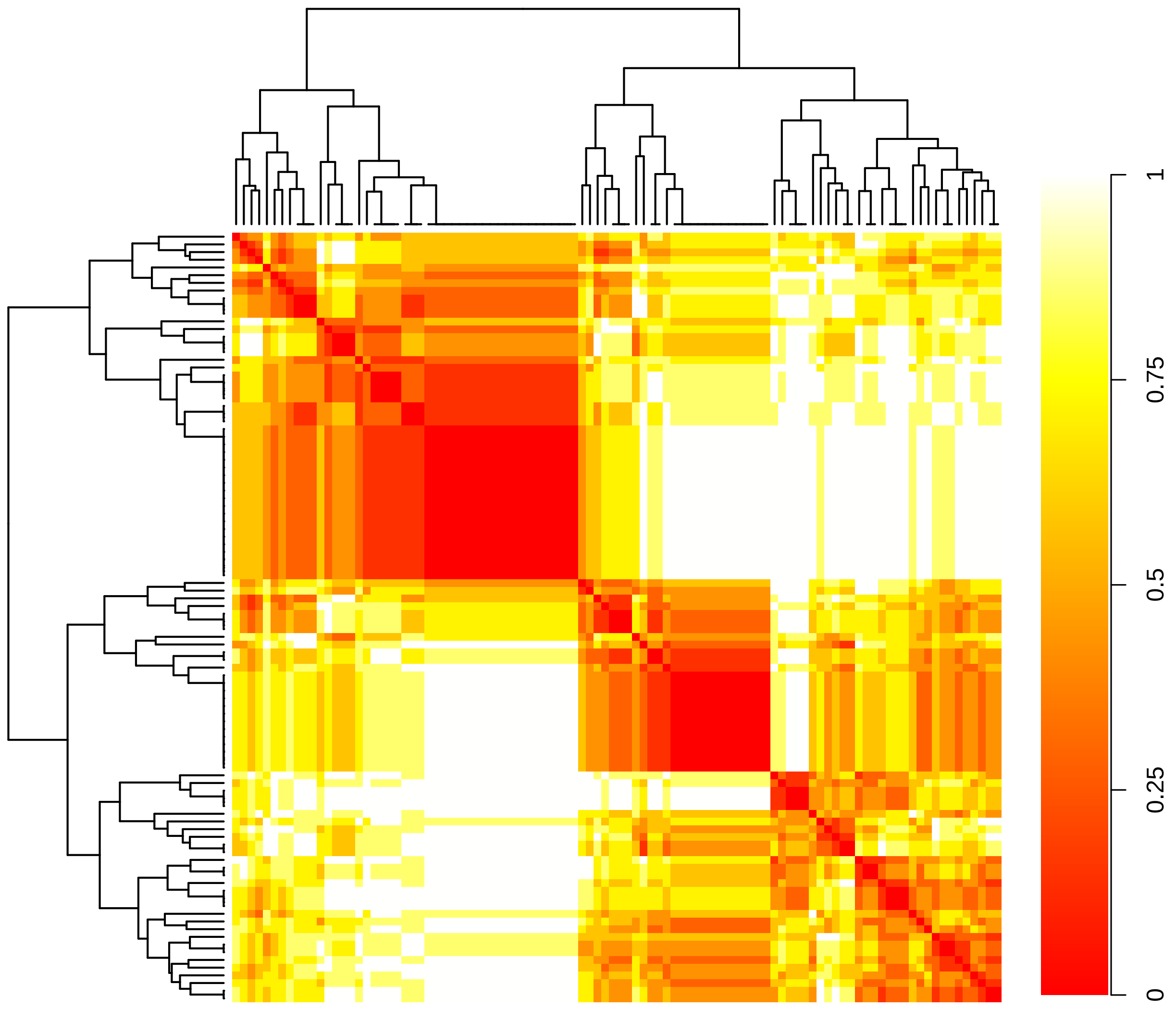

Figure 9: Pairwise secondary structure dissimilarity (1-Q3) in Cluster 3 (random sample of 100 fragments).

4 Structural variation in cluster number 4

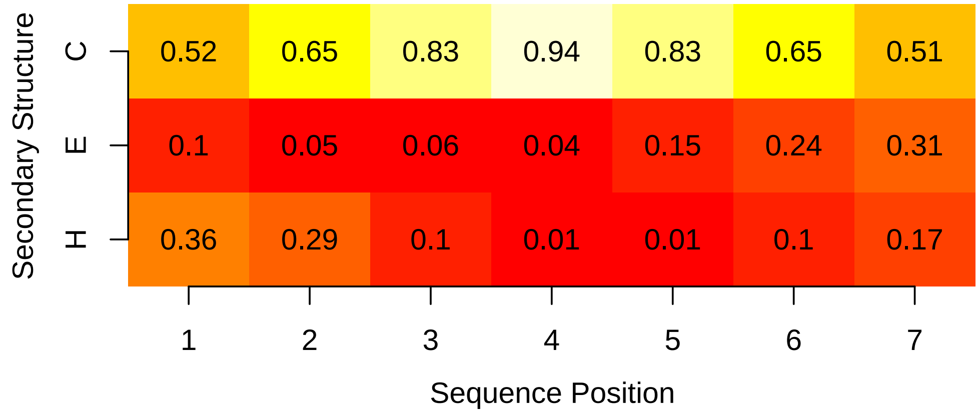

Figure 10: Position-specific secondary structure (STRIDE) in cluster 4.

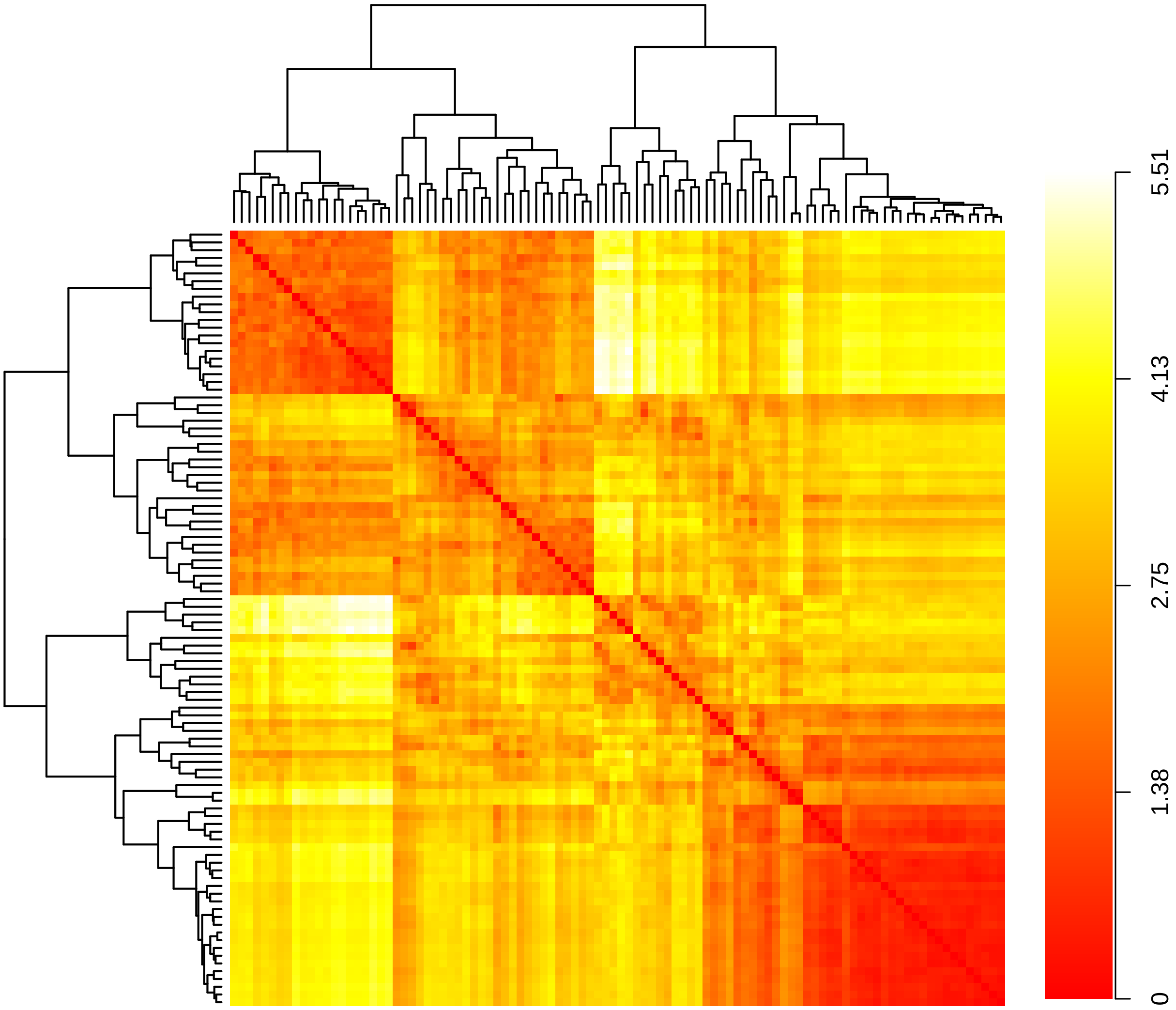

Figure 11: Pairwise RMSD in Cluster 4 (random sample of 100 fragments).

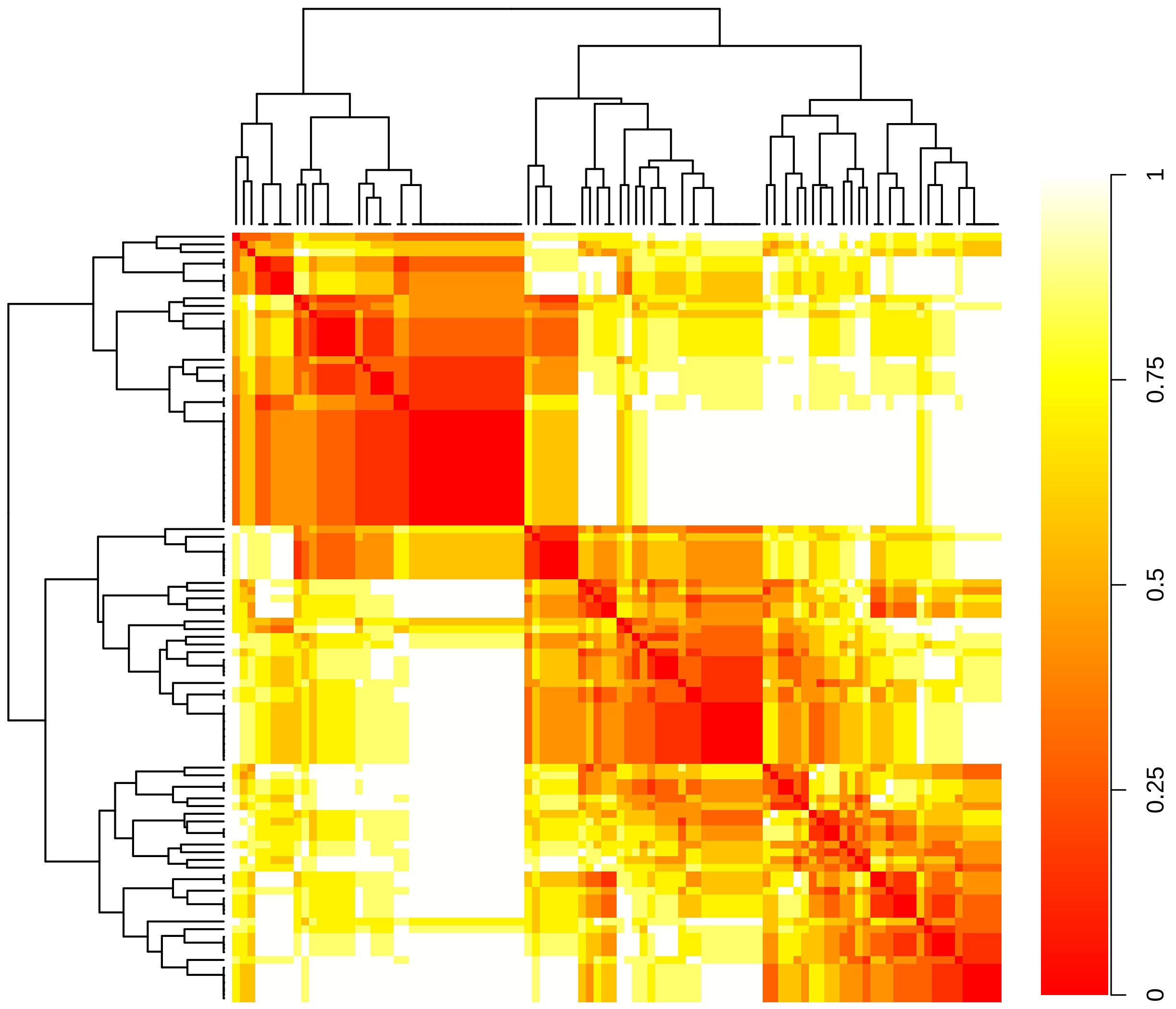

Figure 12: Pairwise secondary structure dissimilarity (1-Q3) in Cluster 4 (random sample of 100 fragments).

5 Structural variation in cluster number 5

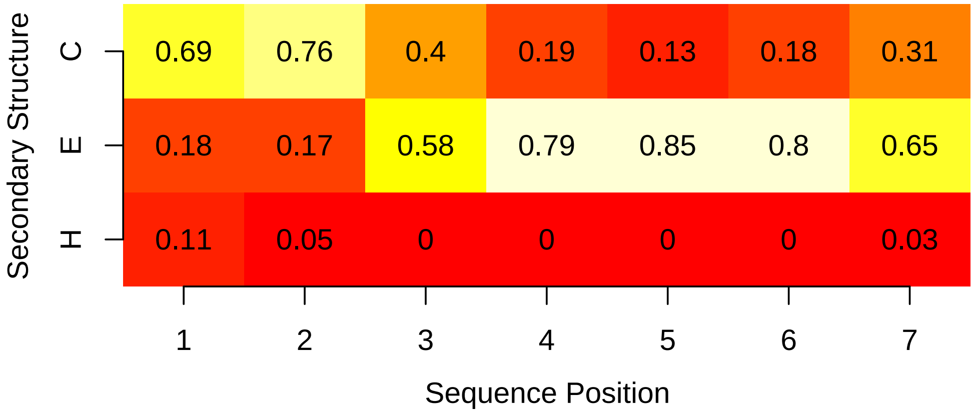

Figure 13: Position-specific secondary structure (STRIDE) in cluster 5.

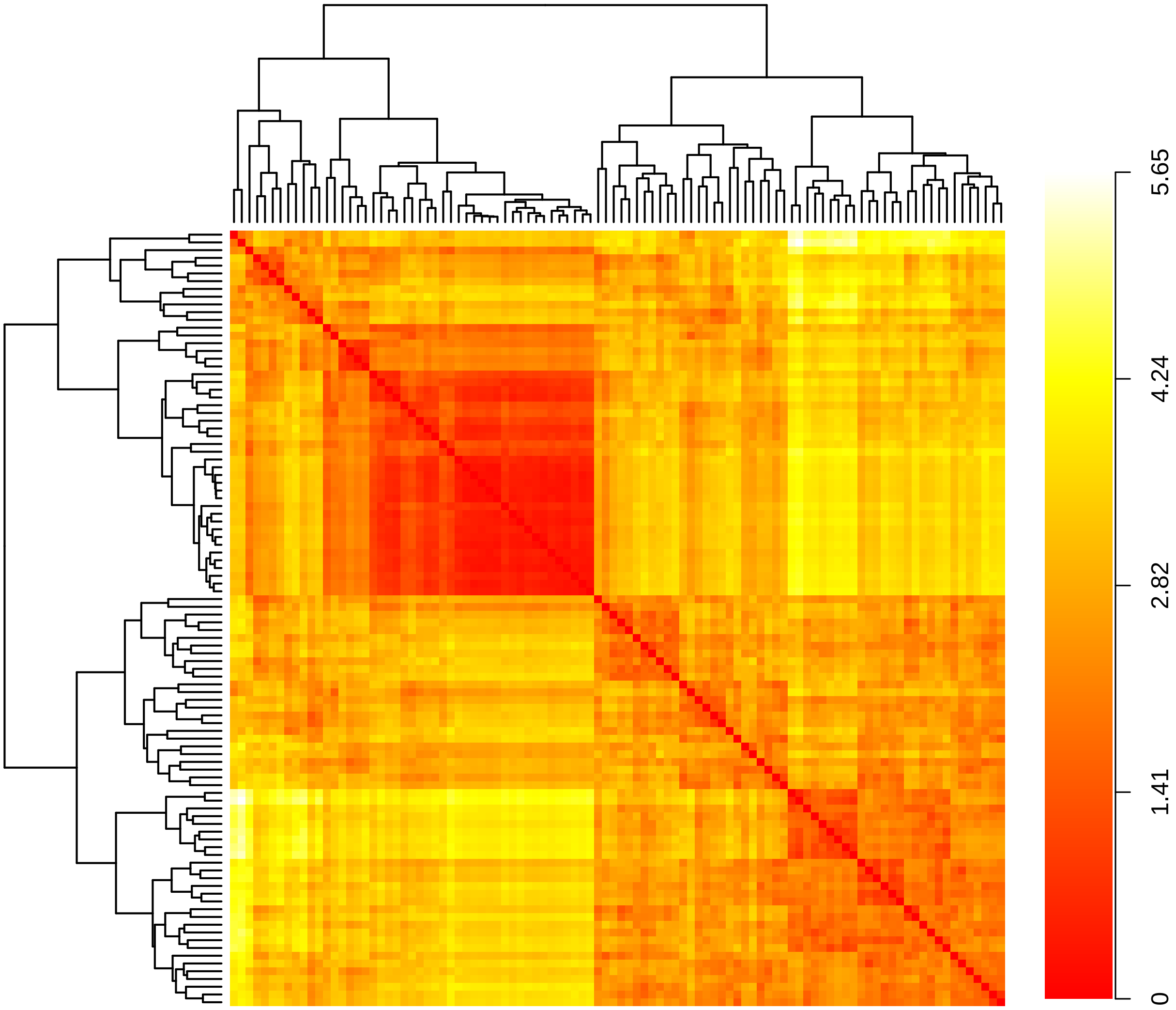

Figure 14: Pairwise RMSD in Cluster 5 (random sample of 100 fragments).

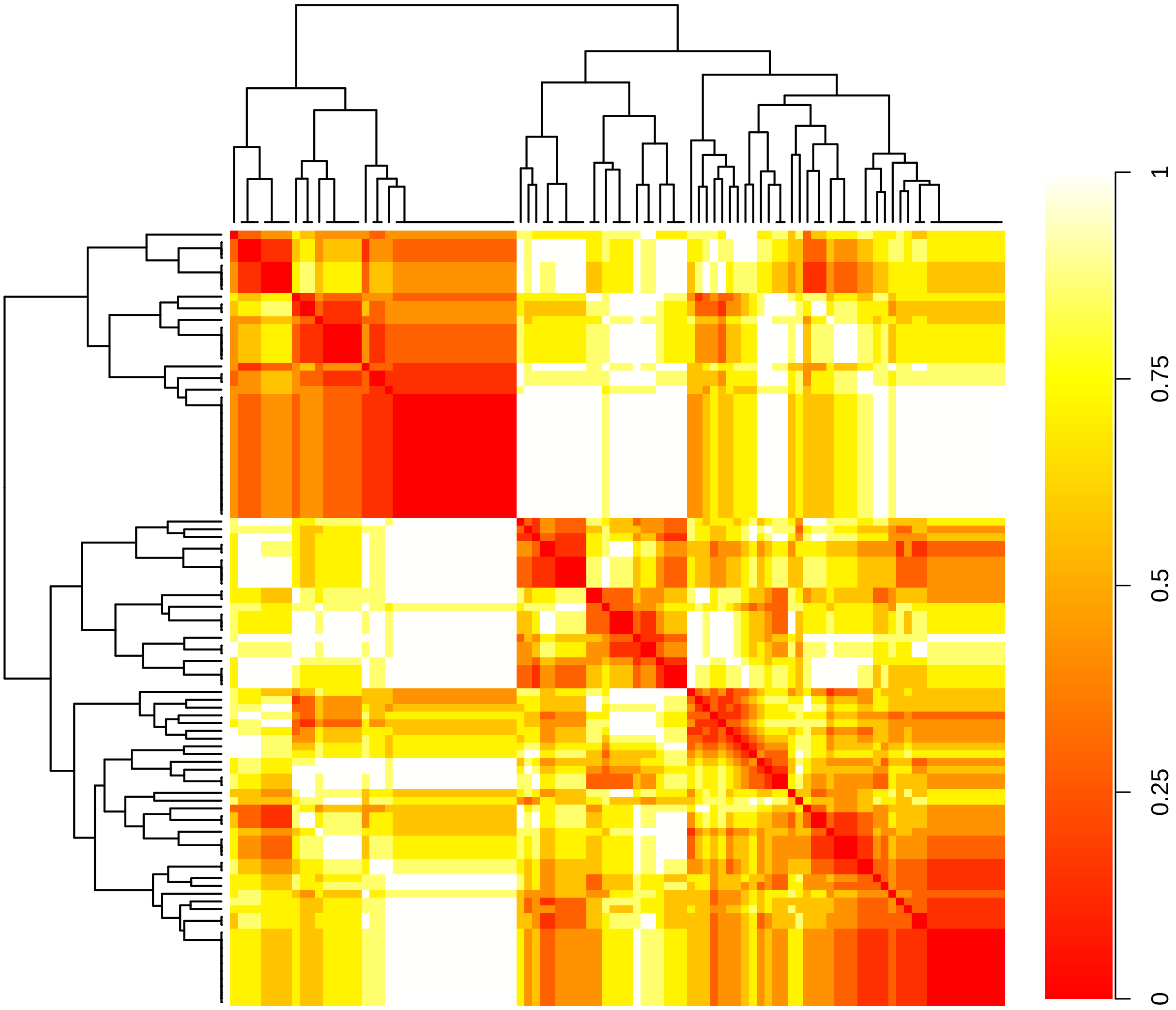

Figure 15: Pairwise secondary structure dissimilarity (1-Q3) in Cluster 5 (random sample of 100 fragments).

6 Structural variation in cluster number 6

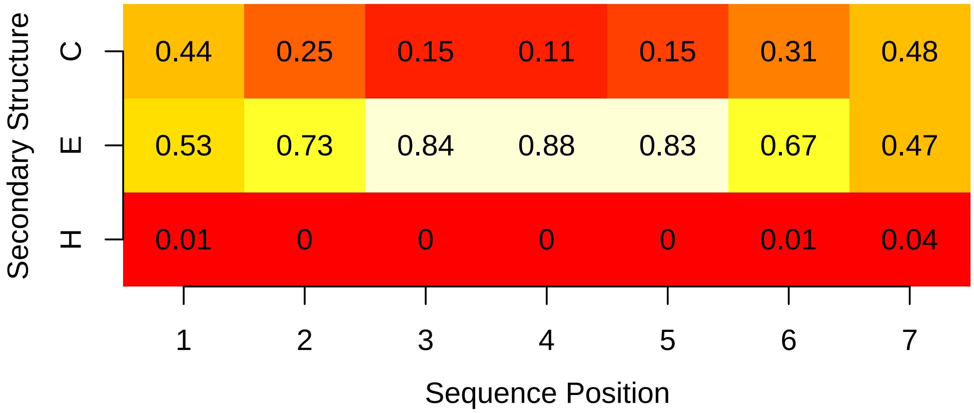

Figure 16: Position-specific secondary structure (STRIDE) in cluster 6.

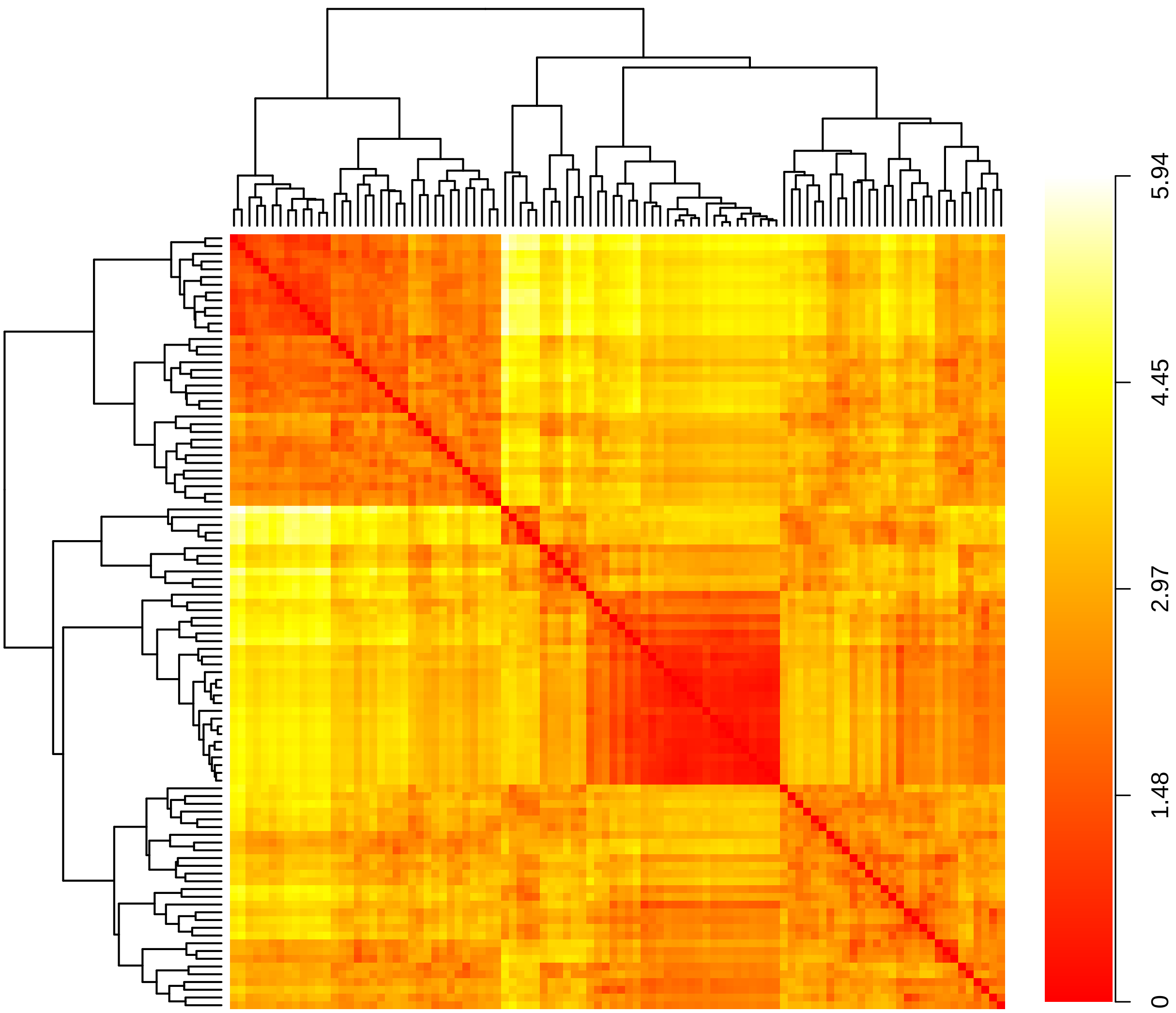

Figure 17: Pairwise RMSD in Cluster 6 (random sample of 100 fragments).

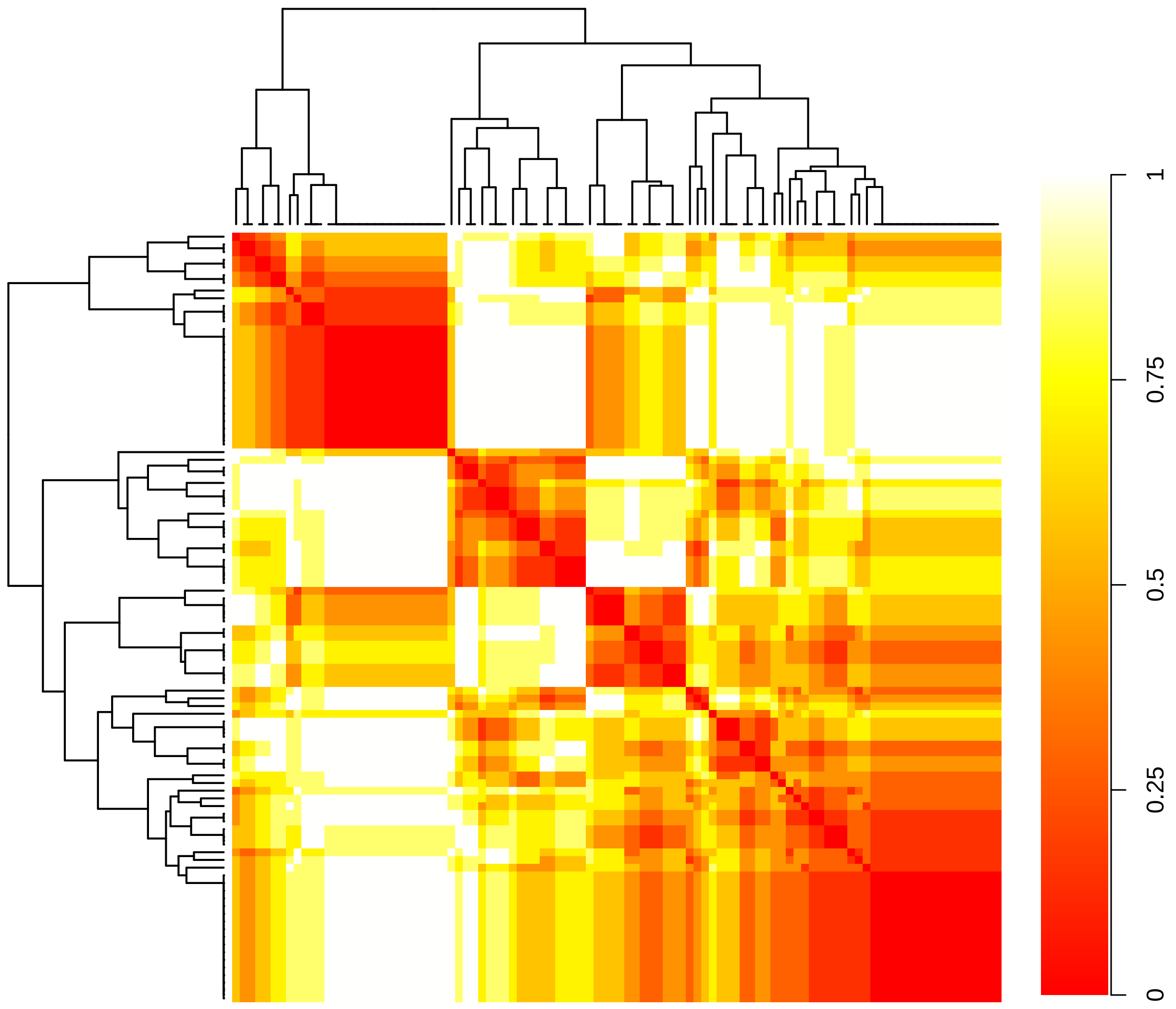

Figure 18: Pairwise secondary structure dissimilarity (1-Q3) in Cluster 6 (random sample of 100 fragments).

7 Structural variation in cluster number 7

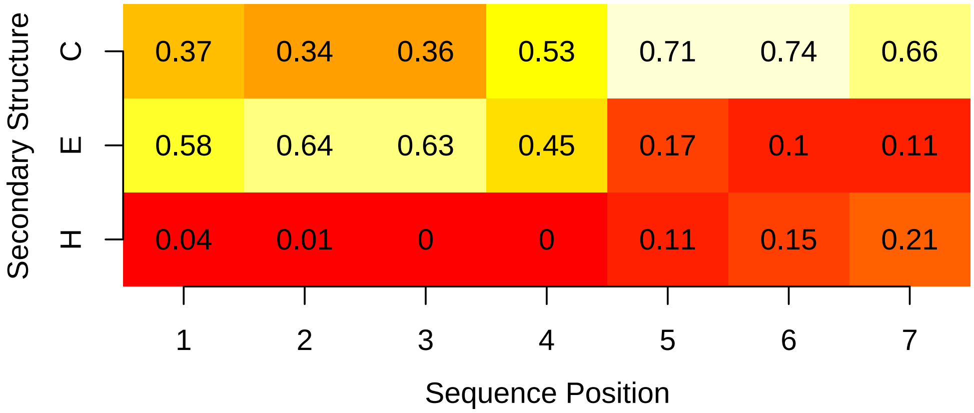

Figure 19: Position-specific secondary structure (STRIDE) in cluster 7.

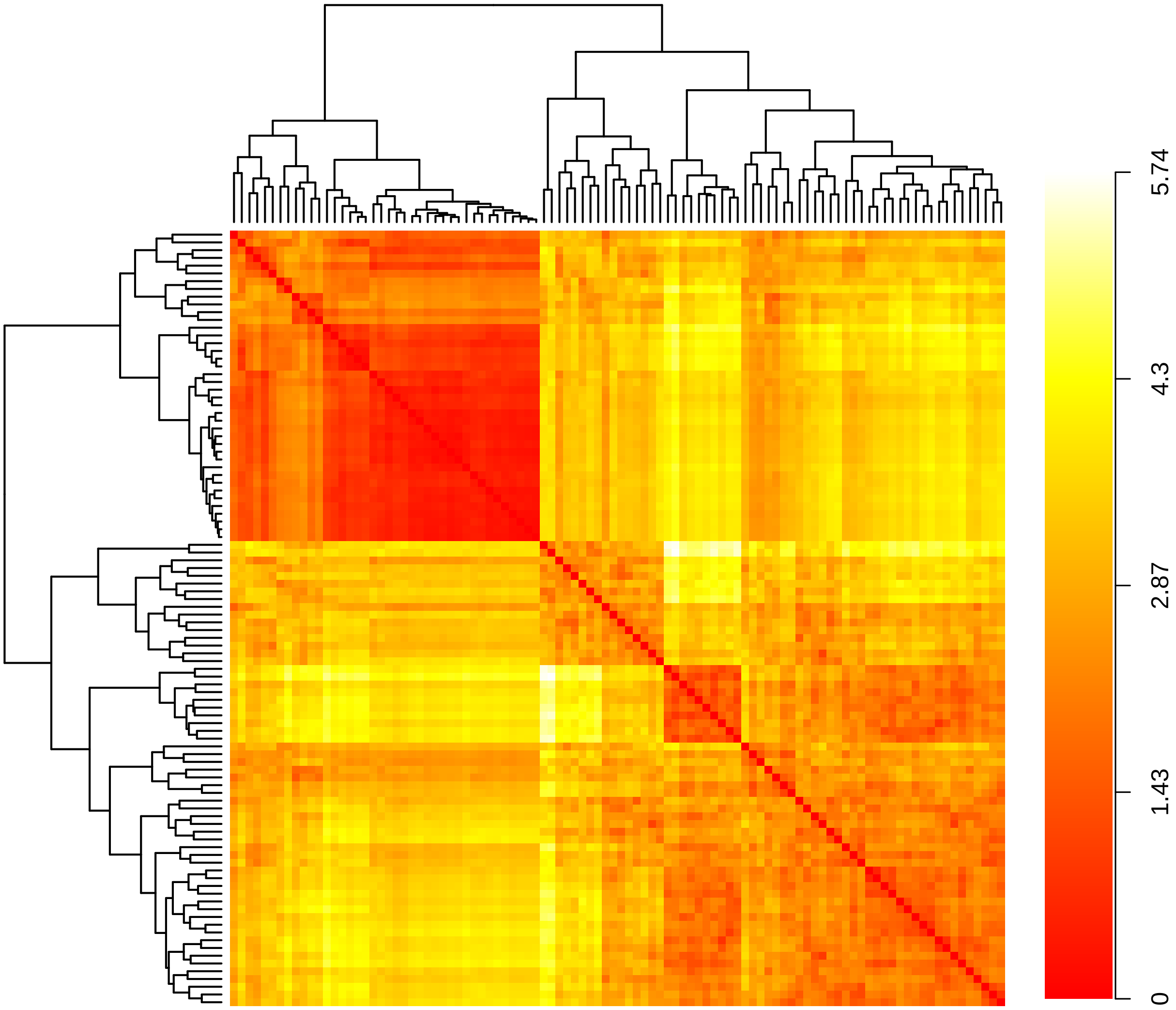

Figure 20: Pairwise RMSD in Cluster 7 (random sample of 100 fragments).

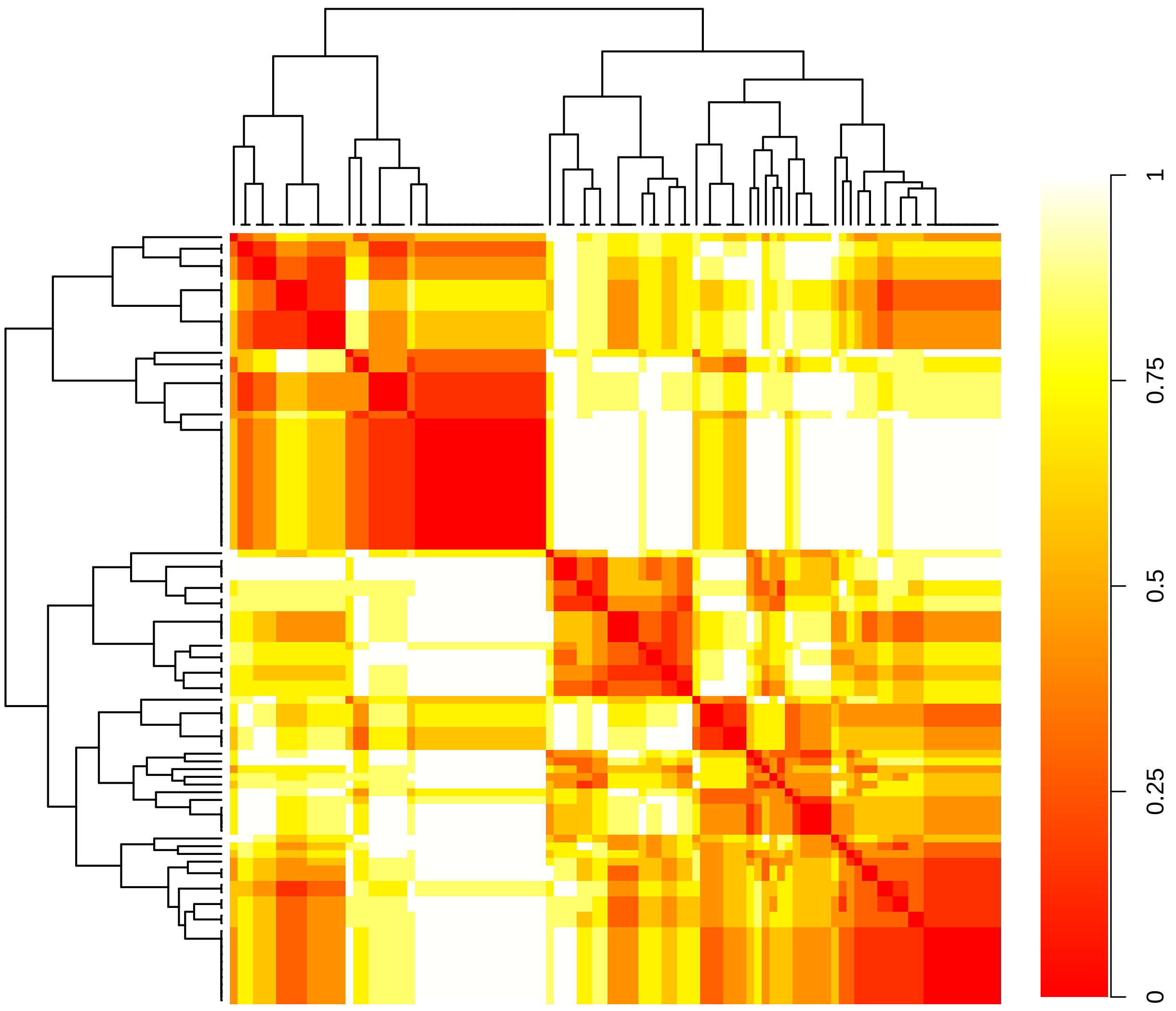

Figure 21: Pairwise secondary structure dissimilarity (1-Q3) in Cluster 7 (random sample of 100 fragments).

8 Structural variation in cluster number 8

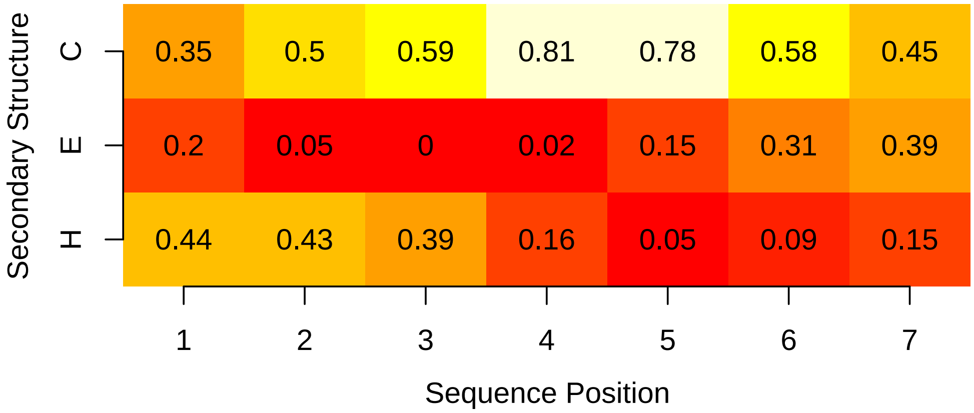

Figure 22: Position-specific secondary structure (STRIDE) in cluster 8.

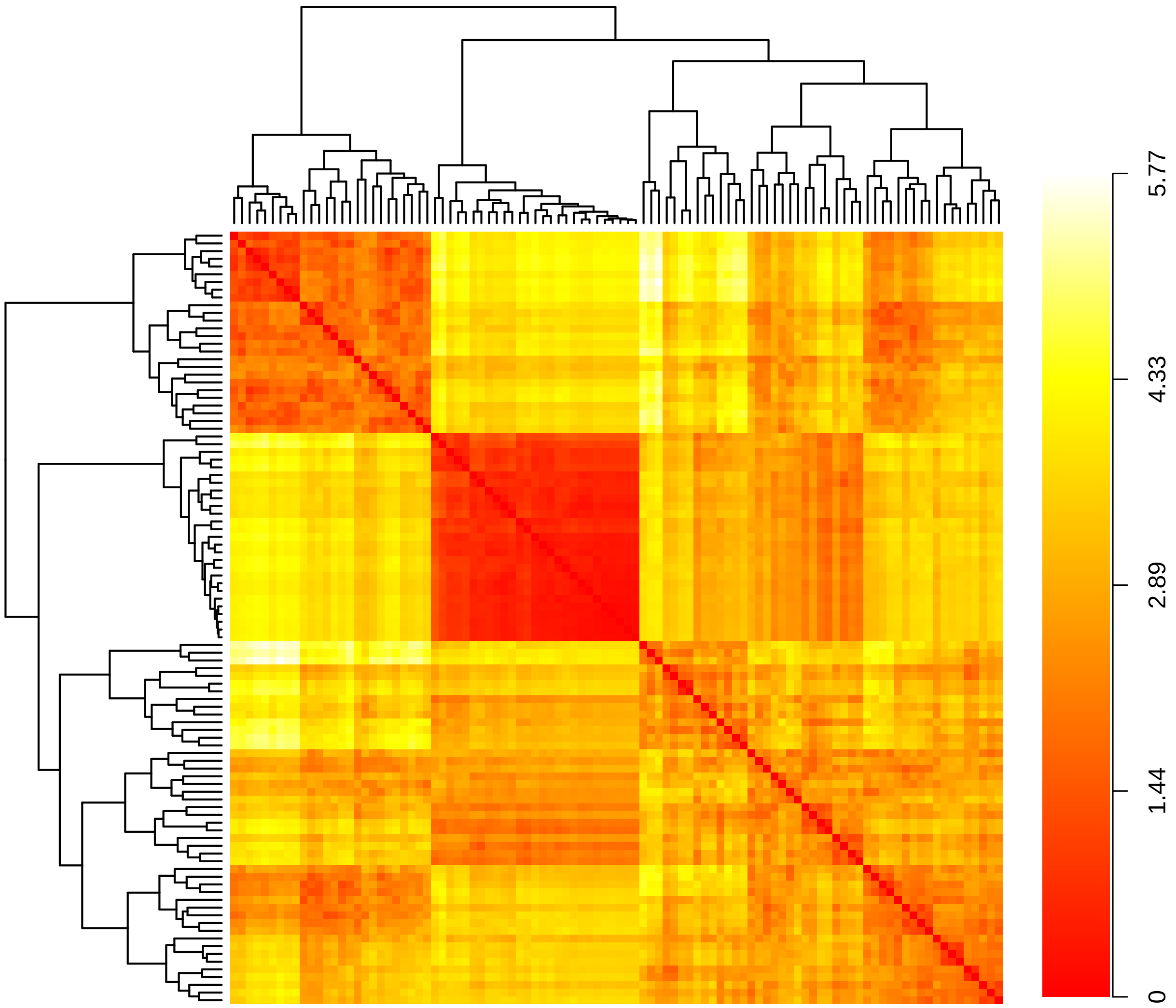

Figure 23: Pairwise RMSD in Cluster 8 (random sample of 100 fragments).

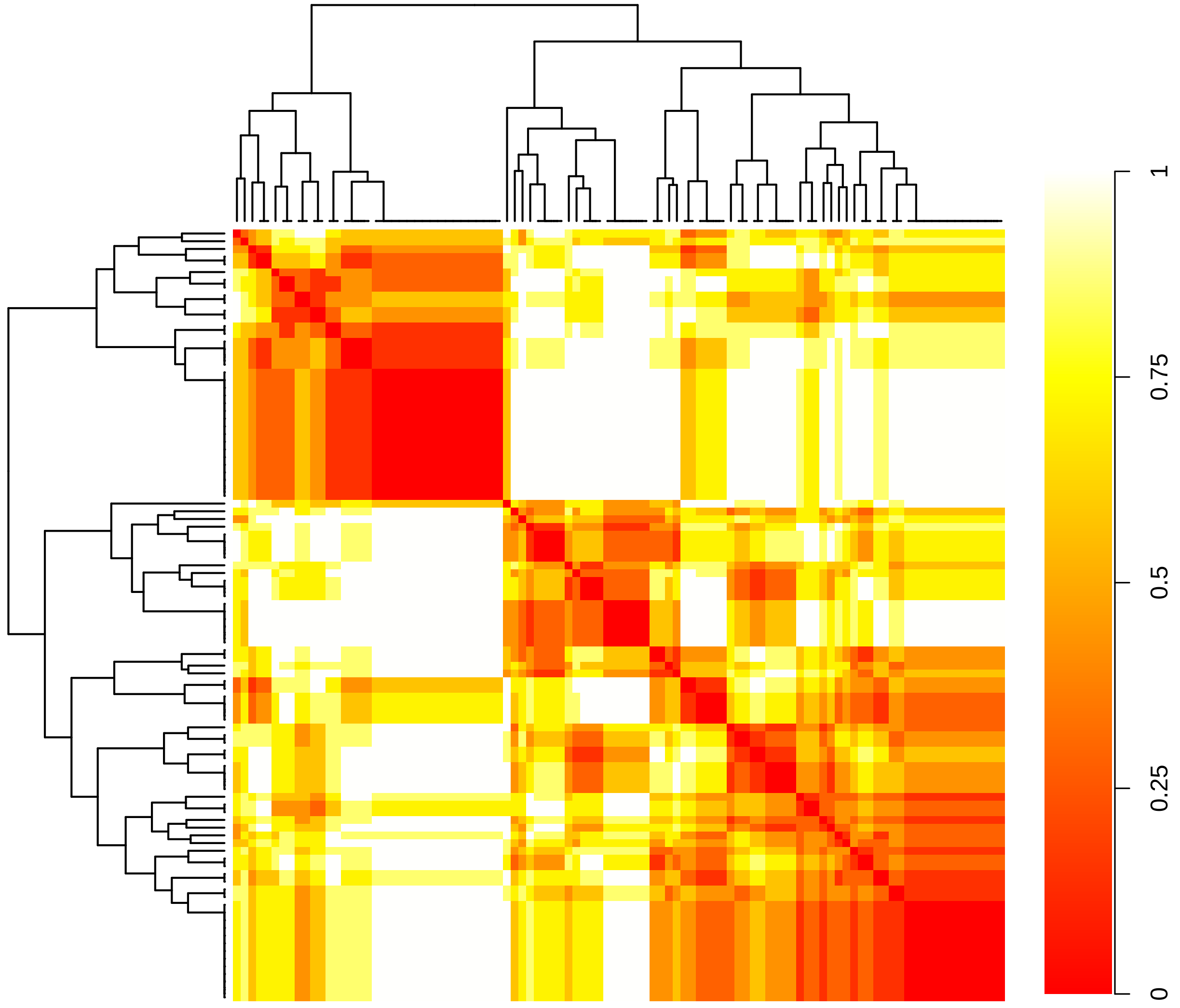

Figure 24: Pairwise secondary structure dissimilarity (1-Q3) in Cluster 8 (random sample of 100 fragments).

9 Structural variation in cluster number 9

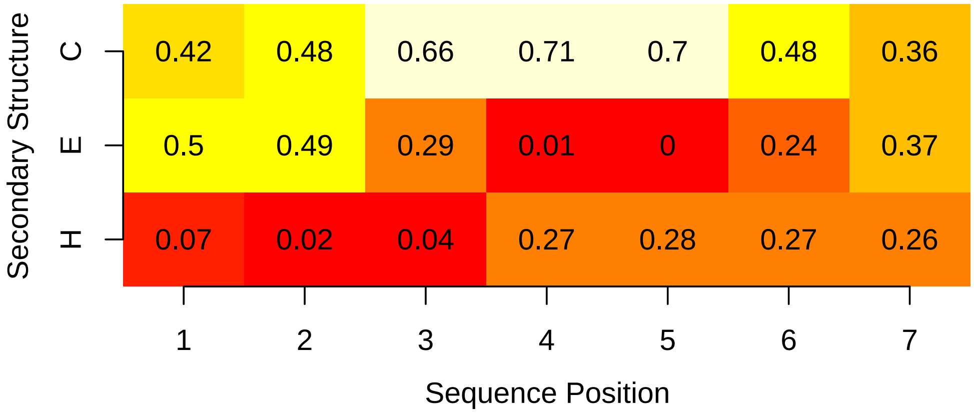

Figure 25: Position-specific secondary structure (STRIDE) in cluster 9.

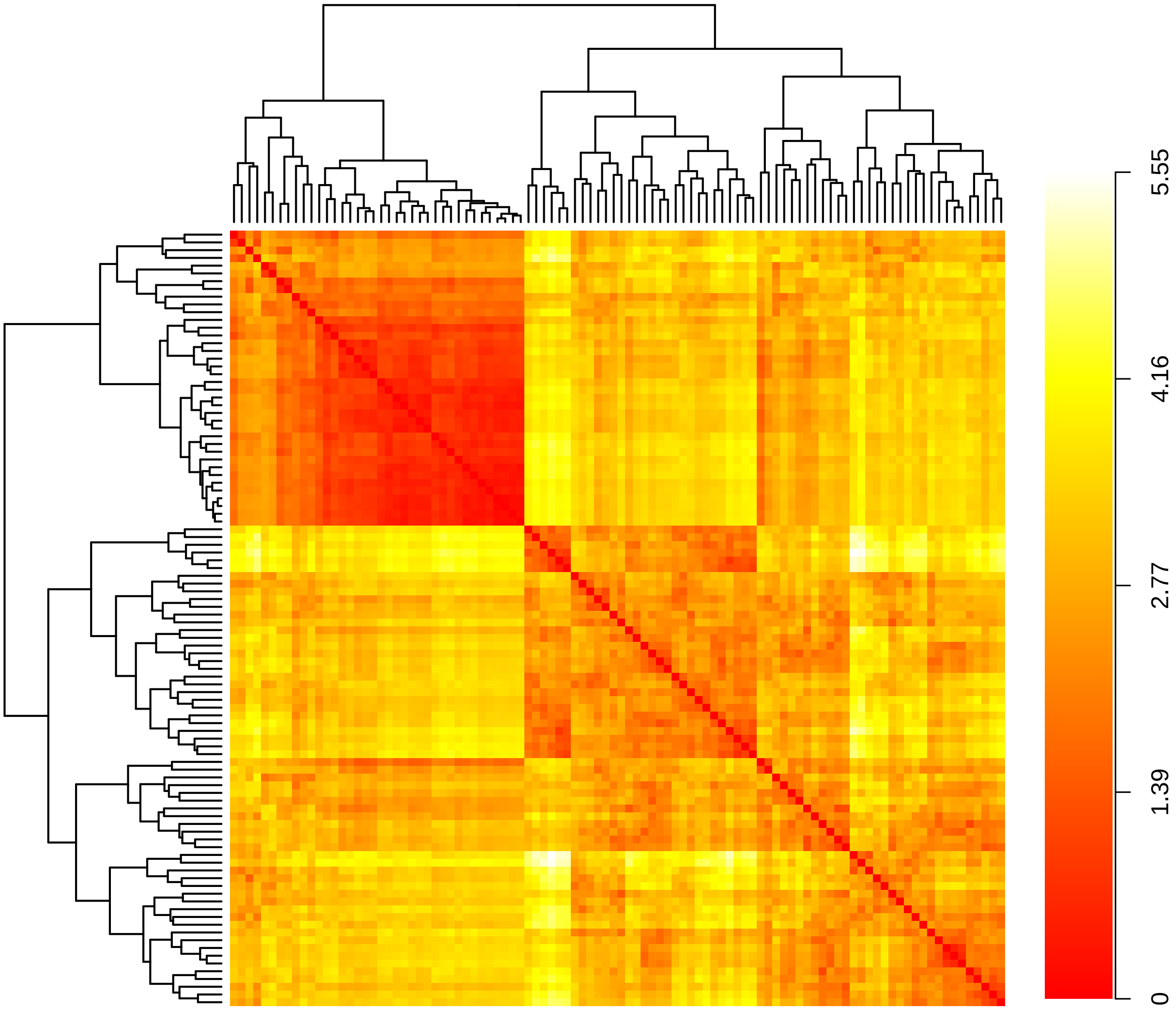

Figure 26: Pairwise RMSD in Cluster 9 (random sample of 100 fragments).

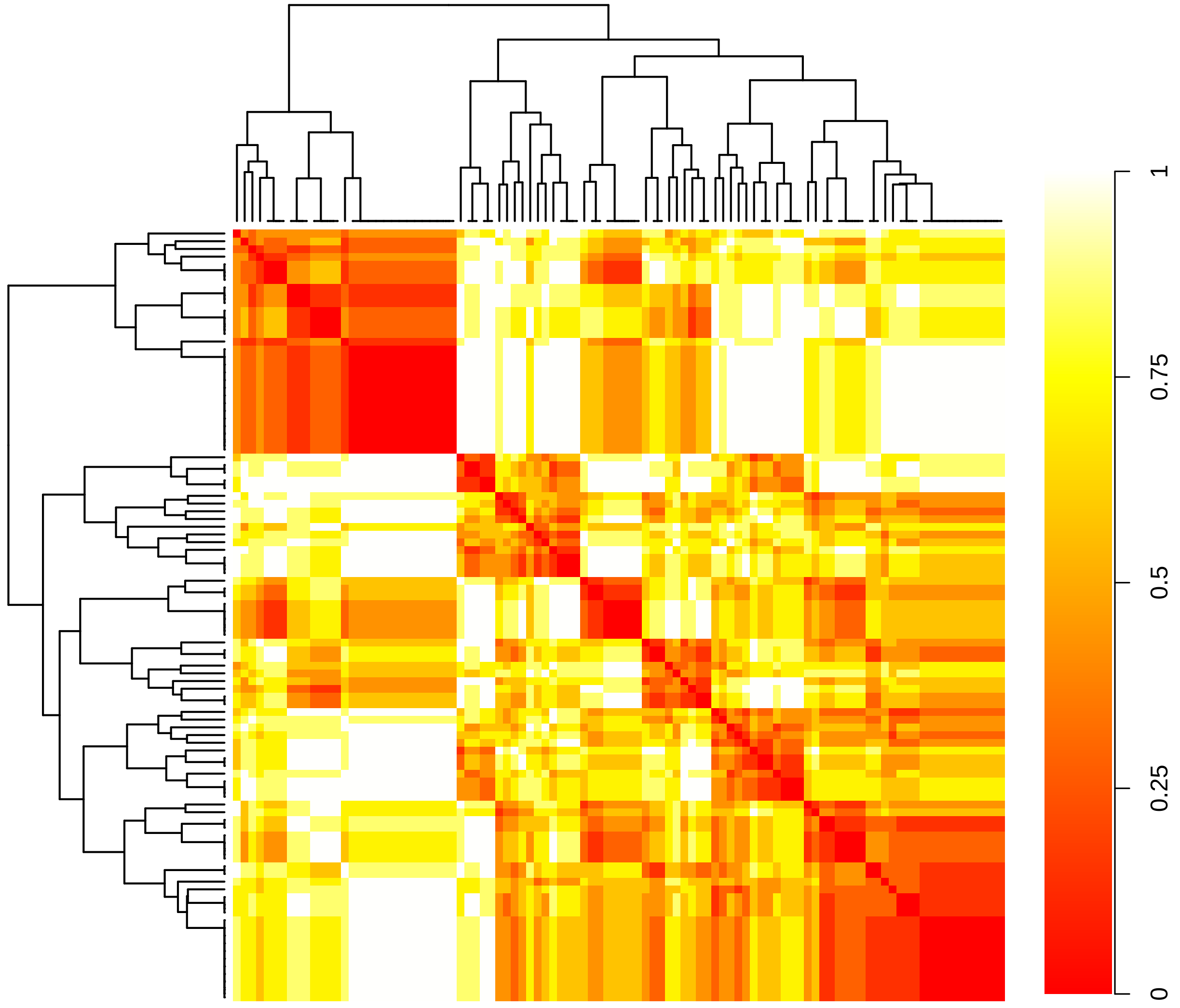

Figure 27: Pairwise secondary structure dissimilarity (1-Q3) in Cluster 9 (random sample of 100 fragments).

10 Structural variation in cluster number 10

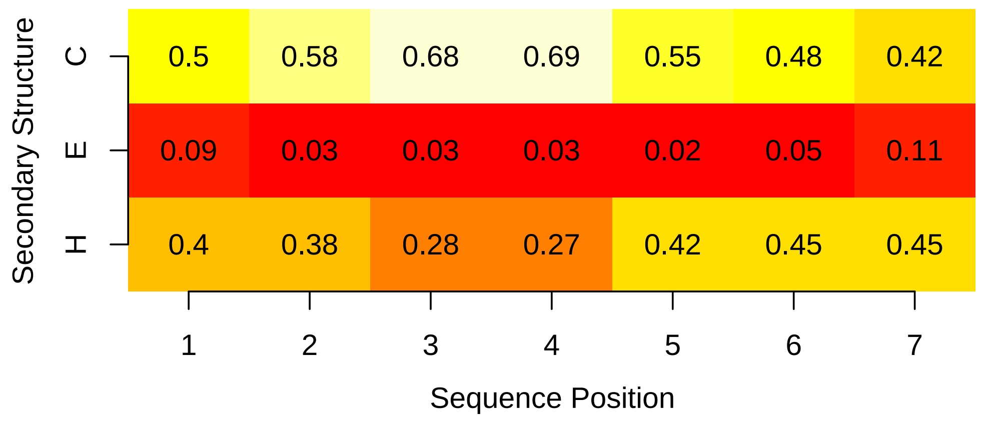

Figure 28: Position-specific secondary structure (STRIDE) in cluster 10.

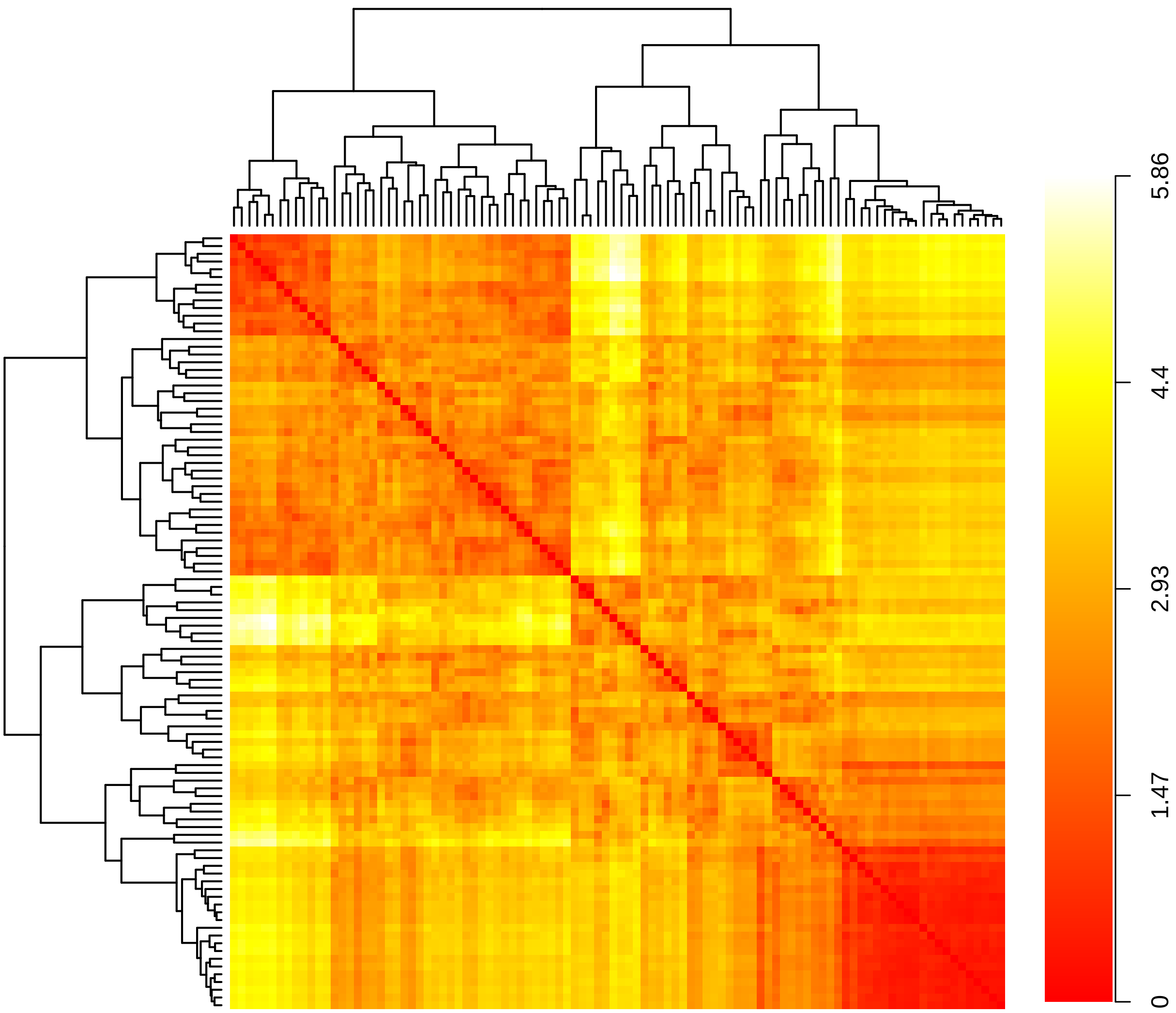

Figure 29: Pairwise RMSD in Cluster 10 (random sample of 100 fragments).

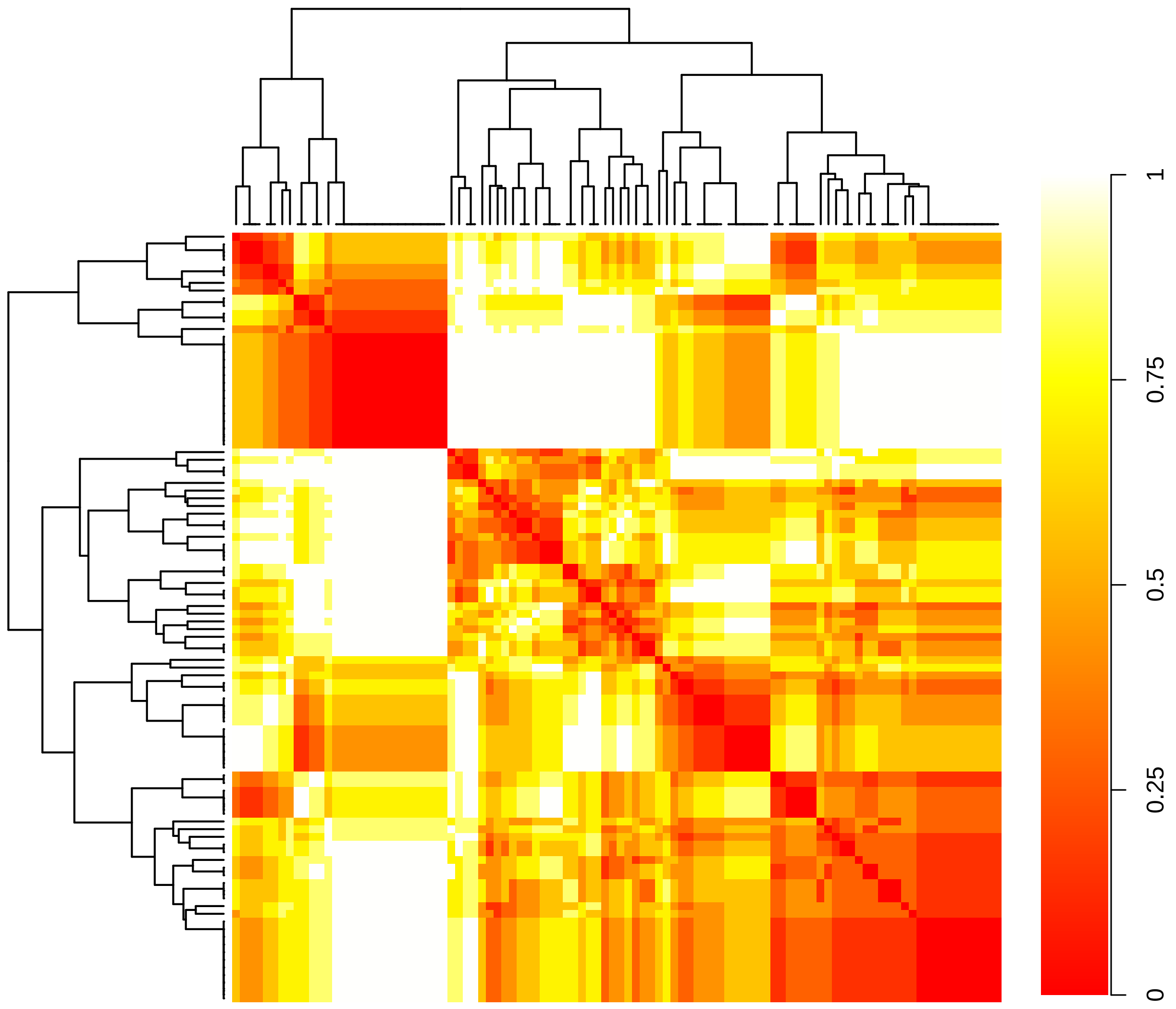

Figure 30: Pairwise secondary structure dissimilarity (1-Q3) in Cluster 10 (random sample of 100 fragments).

11 Structural variation in cluster number 11

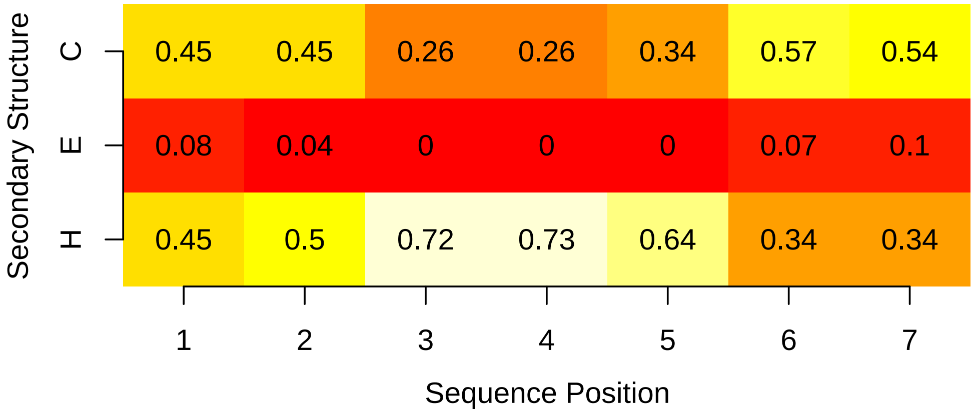

Figure 31: Position-specific secondary structure (STRIDE) in cluster 11.

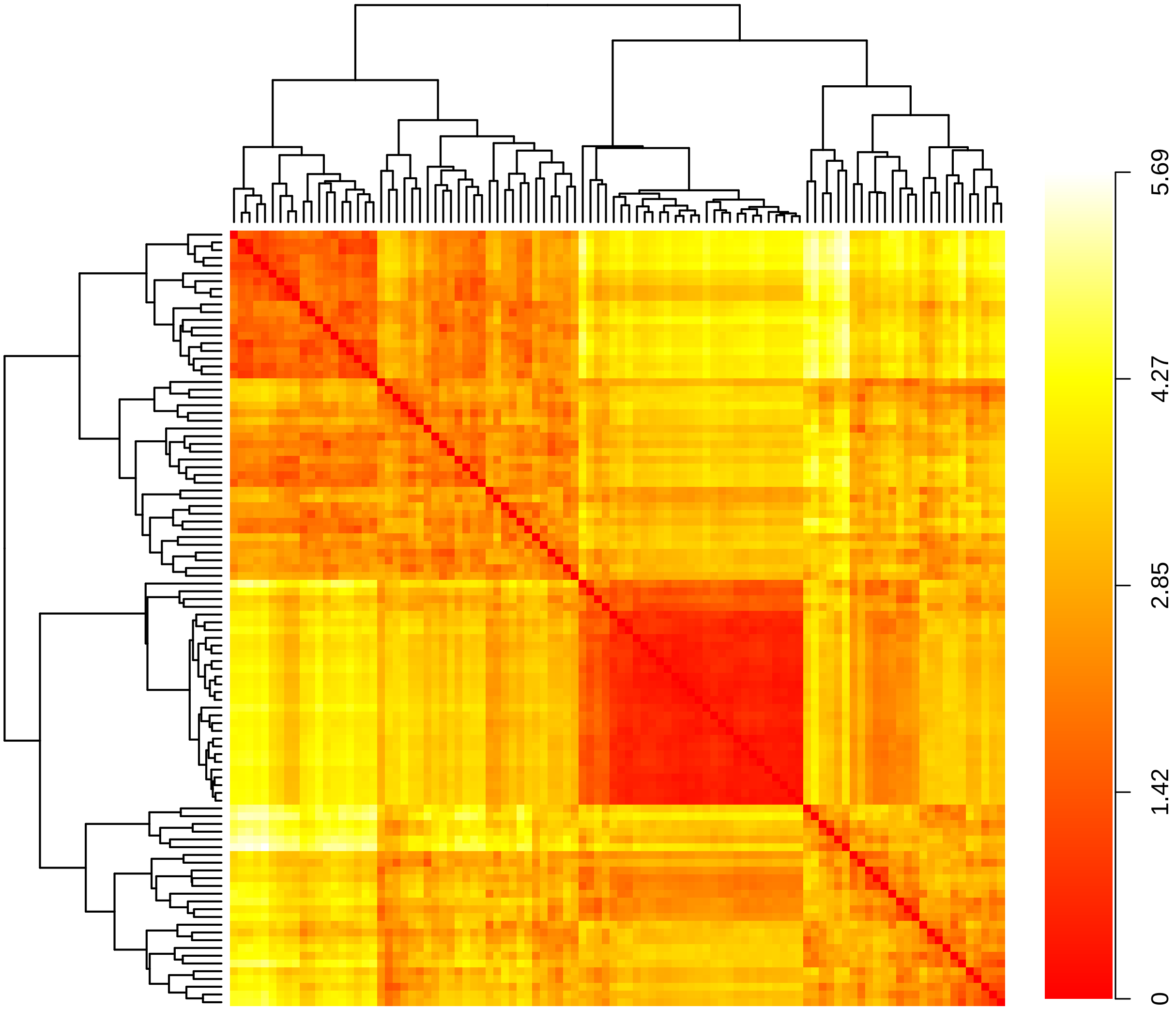

Figure 32: Pairwise RMSD in Cluster 11 (random sample of 100 fragments).

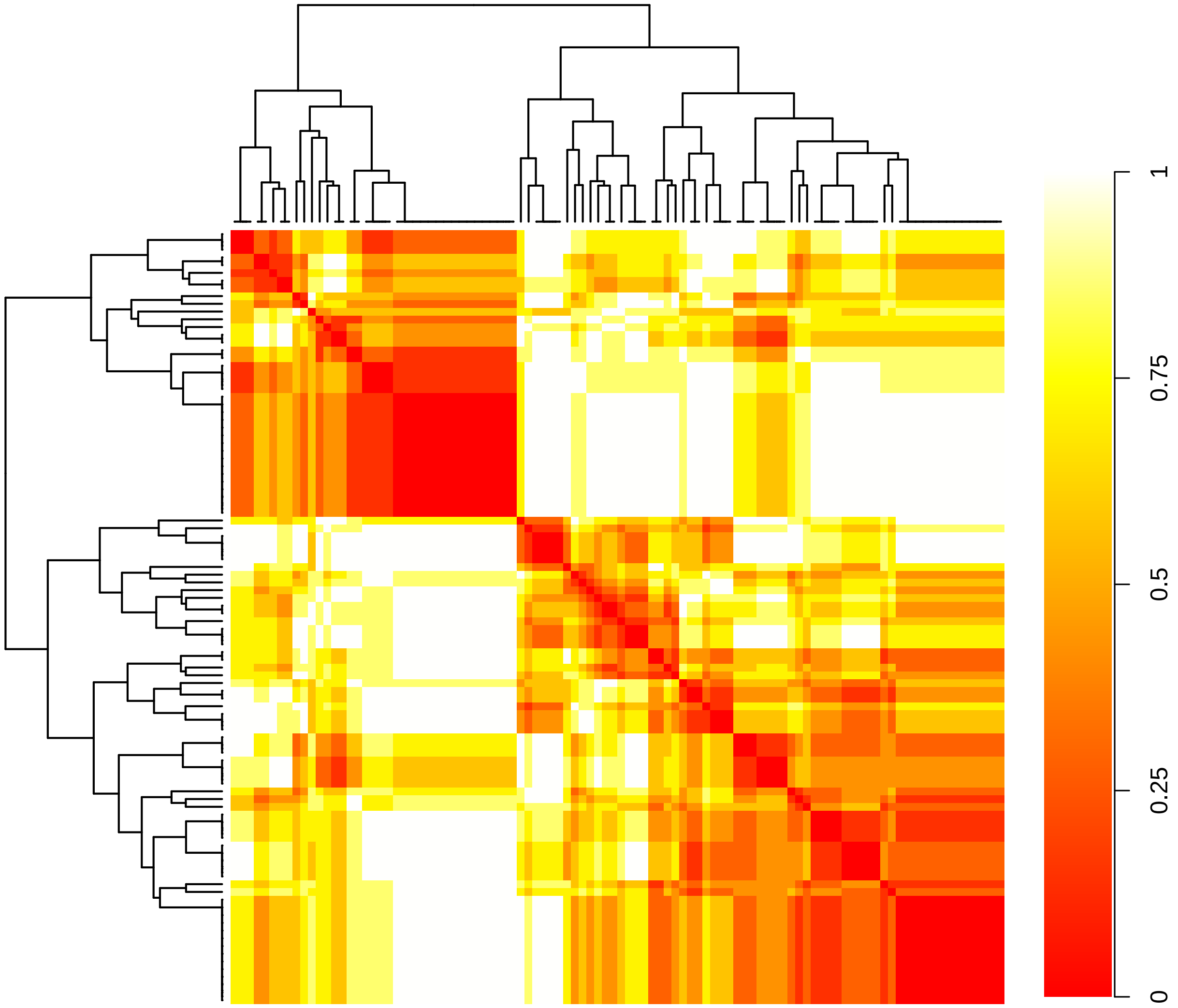

Figure 33: Pairwise secondary structure dissimilarity (1-Q3) in Cluster 11 (random sample of 100 fragments).

12 Structural variation in cluster number 12

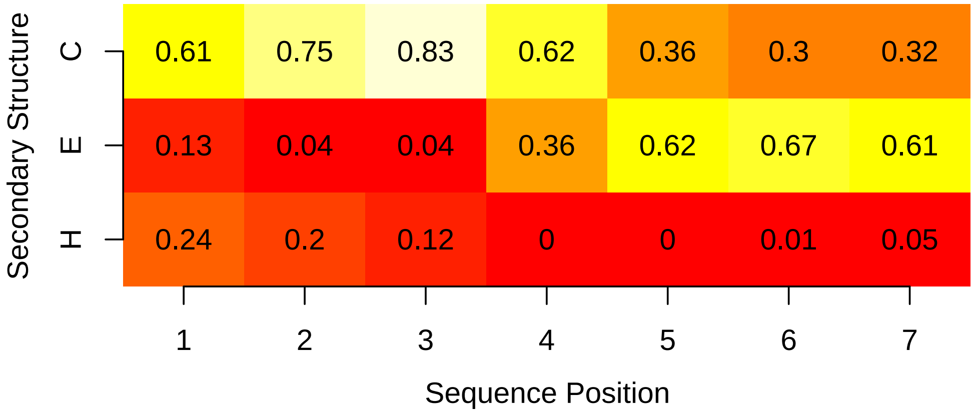

Figure 34: Position-specific secondary structure (STRIDE) in cluster 12.

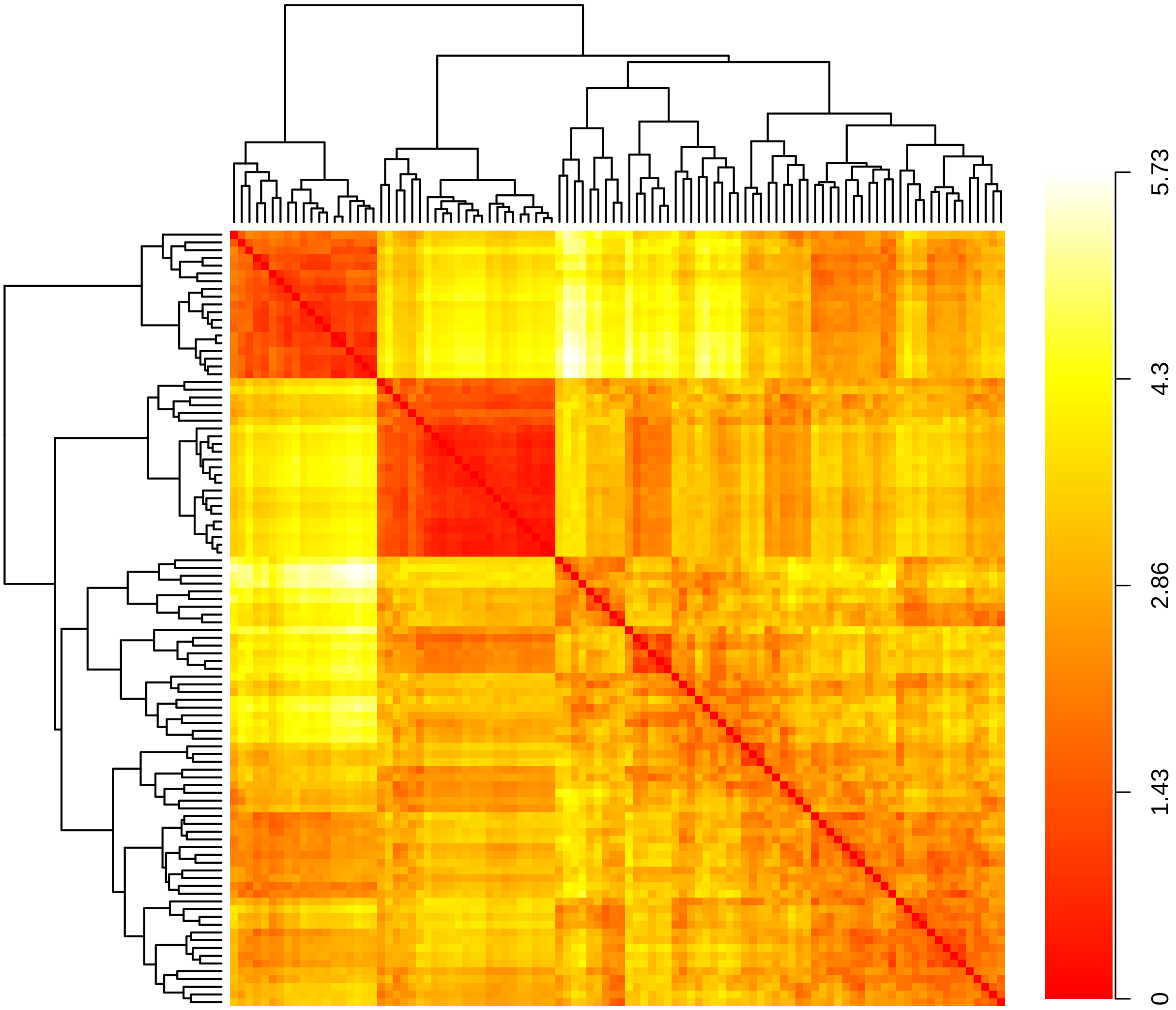

Figure 35: Pairwise RMSD in Cluster 12 (random sample of 100 fragments).

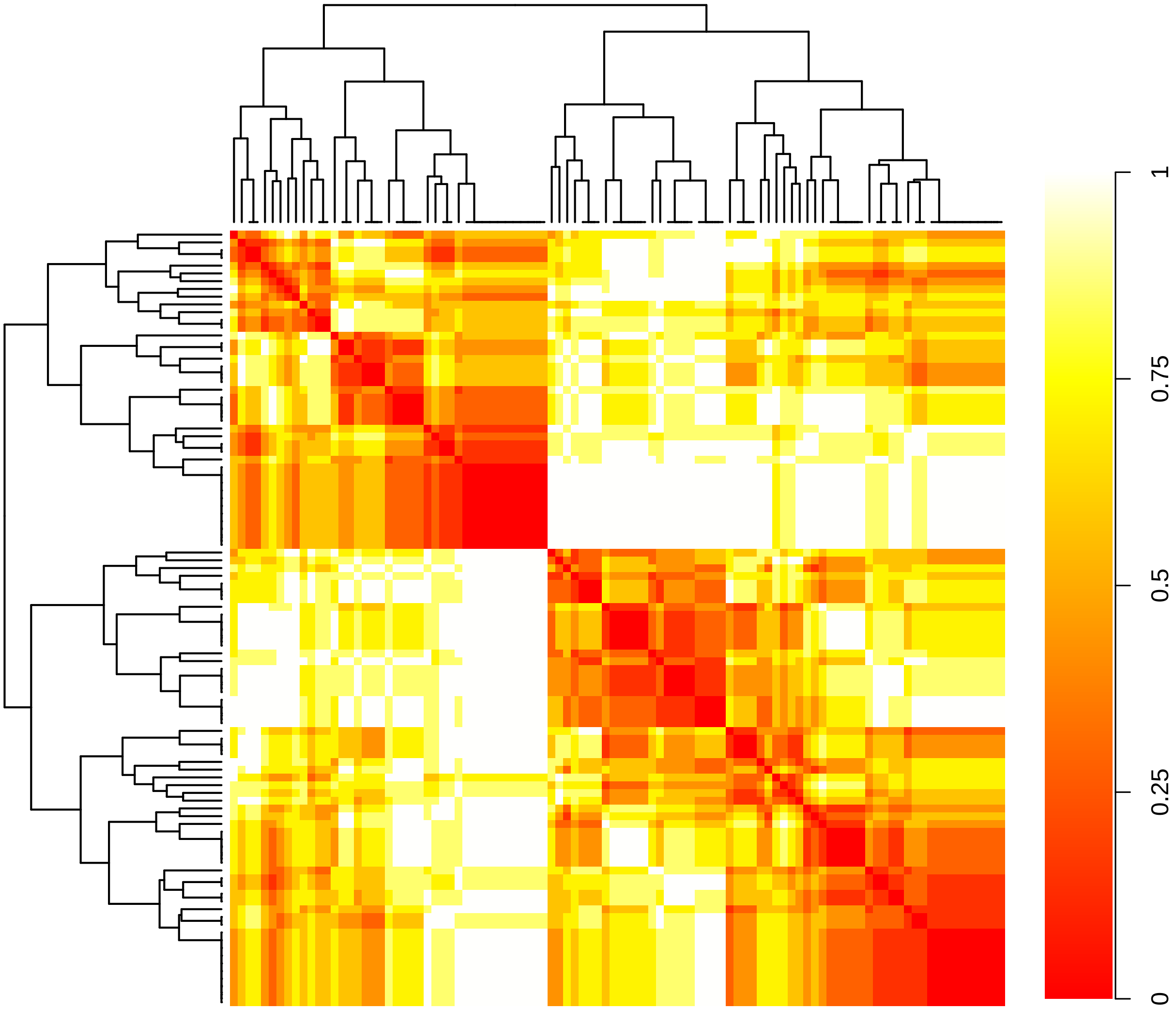

Figure 36: Pairwise secondary structure dissimilarity (1-Q3) in Cluster 12 (random sample of 100 fragments).

13 Structural variation in cluster number 13

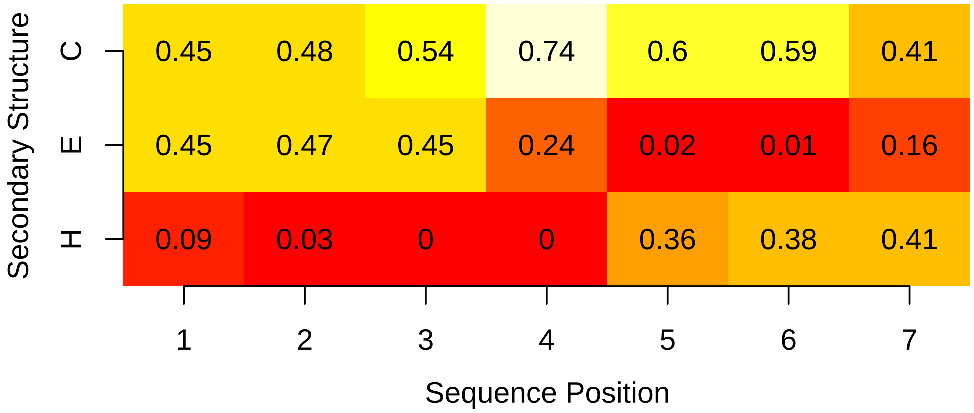

Figure 37: Position-specific secondary structure (STRIDE) in cluster 13.

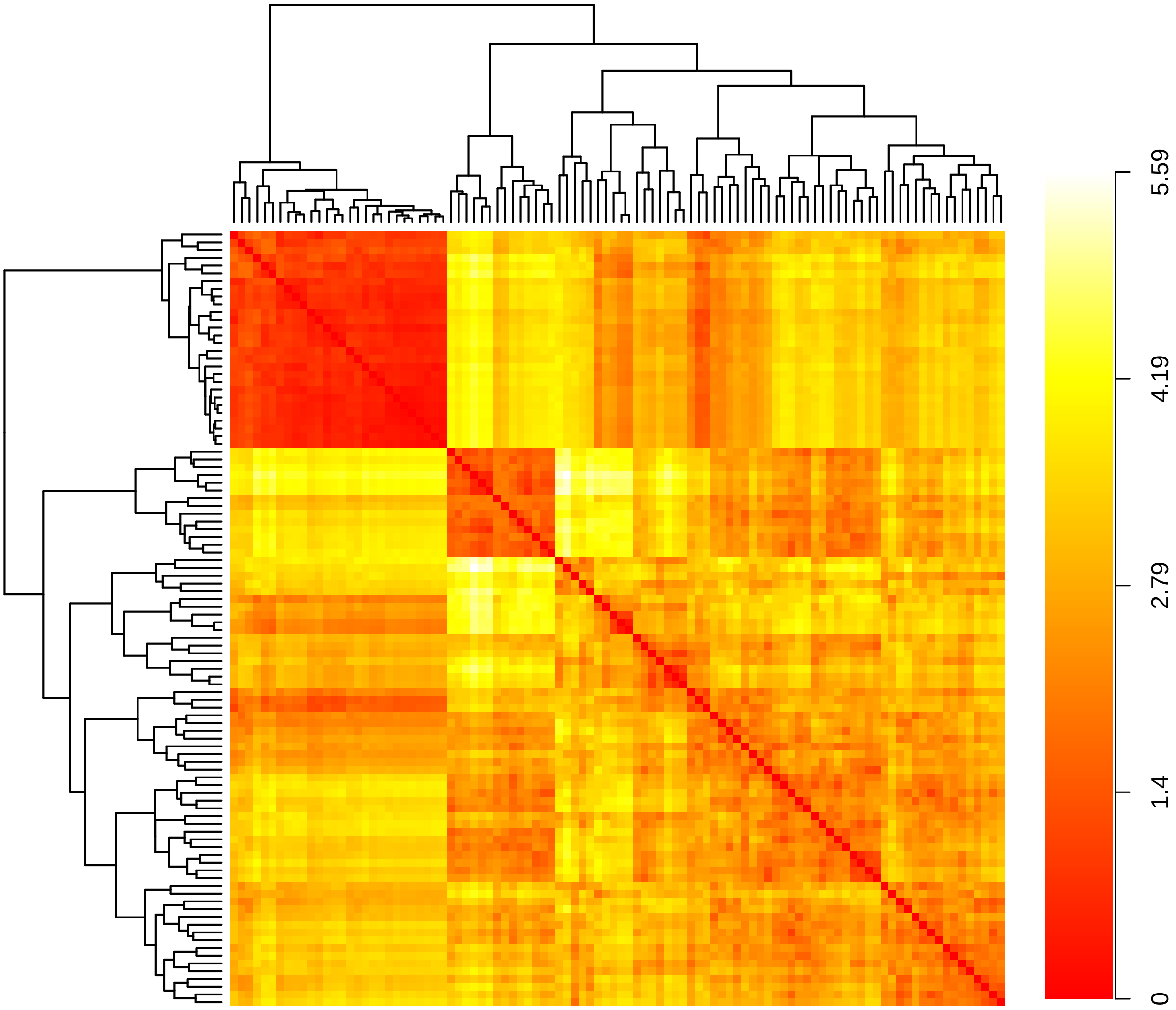

Figure 38: Pairwise RMSD in Cluster 13 (random sample of 100 fragments).

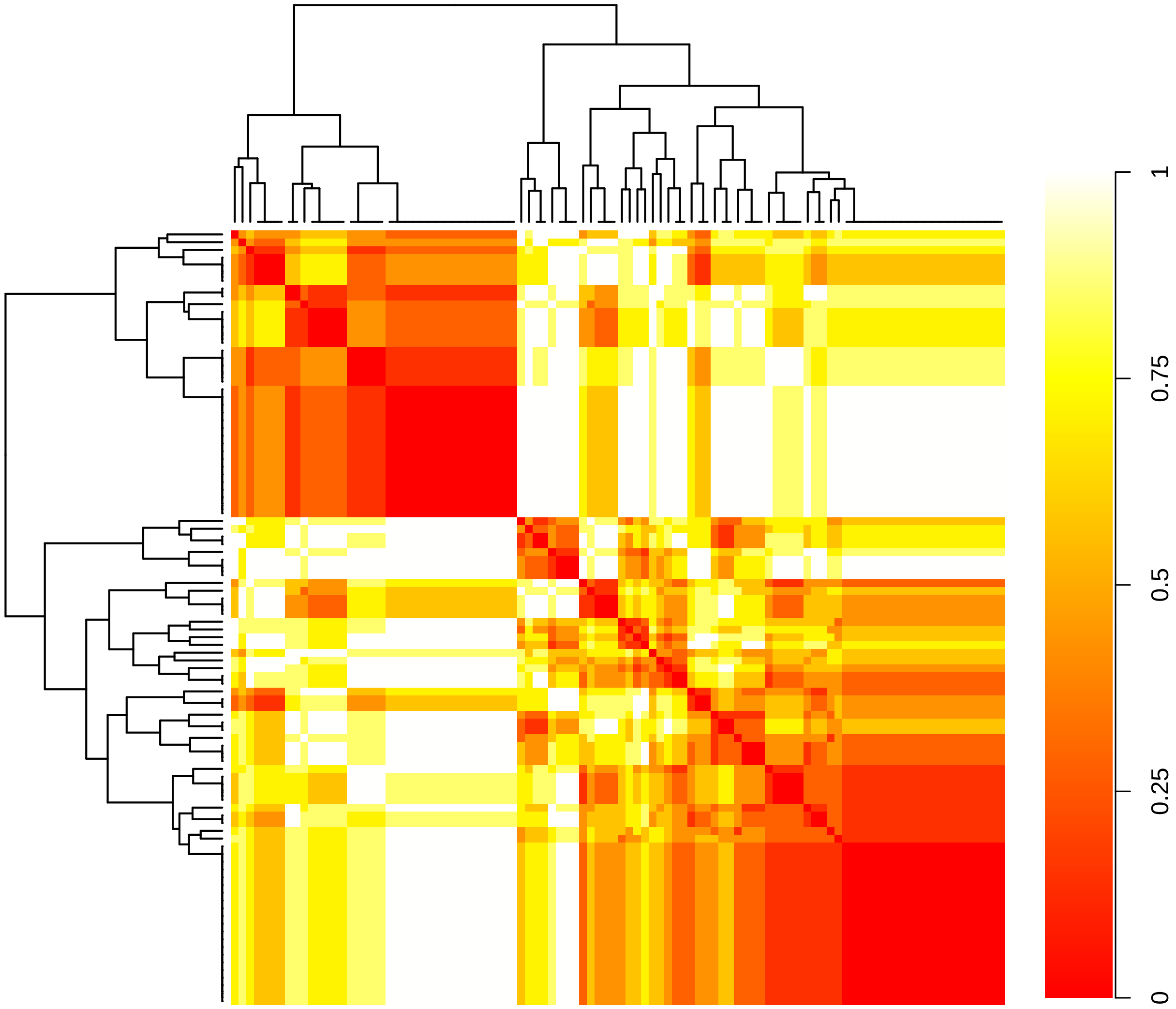

Figure 39: Pairwise secondary structure dissimilarity (1-Q3) in Cluster 13 (random sample of 100 fragments).

14 Structural variation in cluster number 14

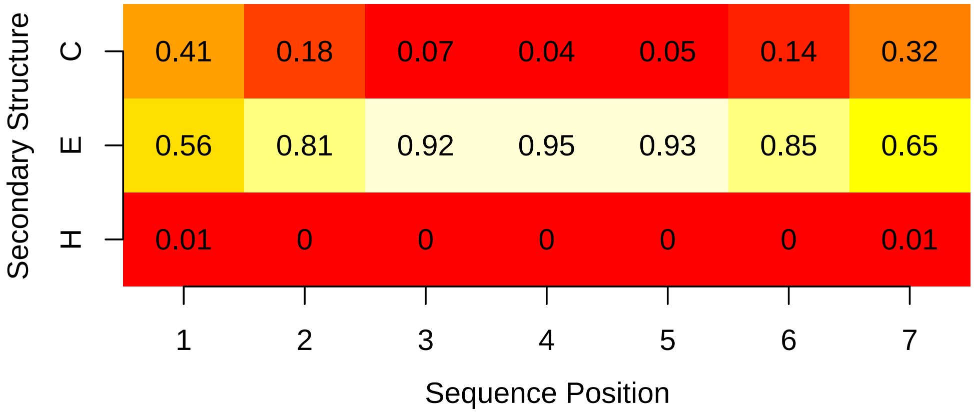

Figure 40: Position-specific secondary structure (STRIDE) in cluster 14.

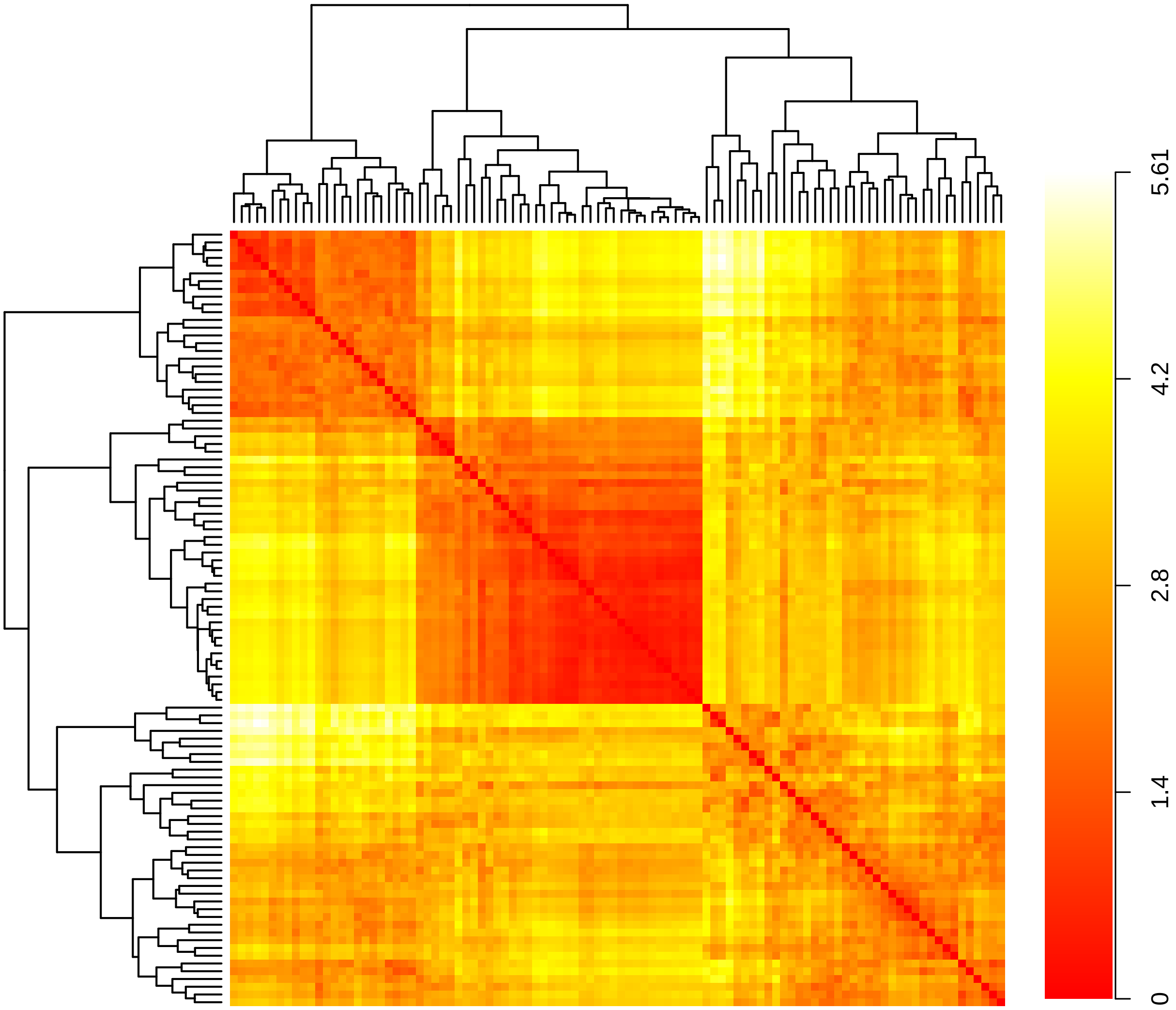

Figure 41: Pairwise RMSD in Cluster 14 (random sample of 100 fragments).

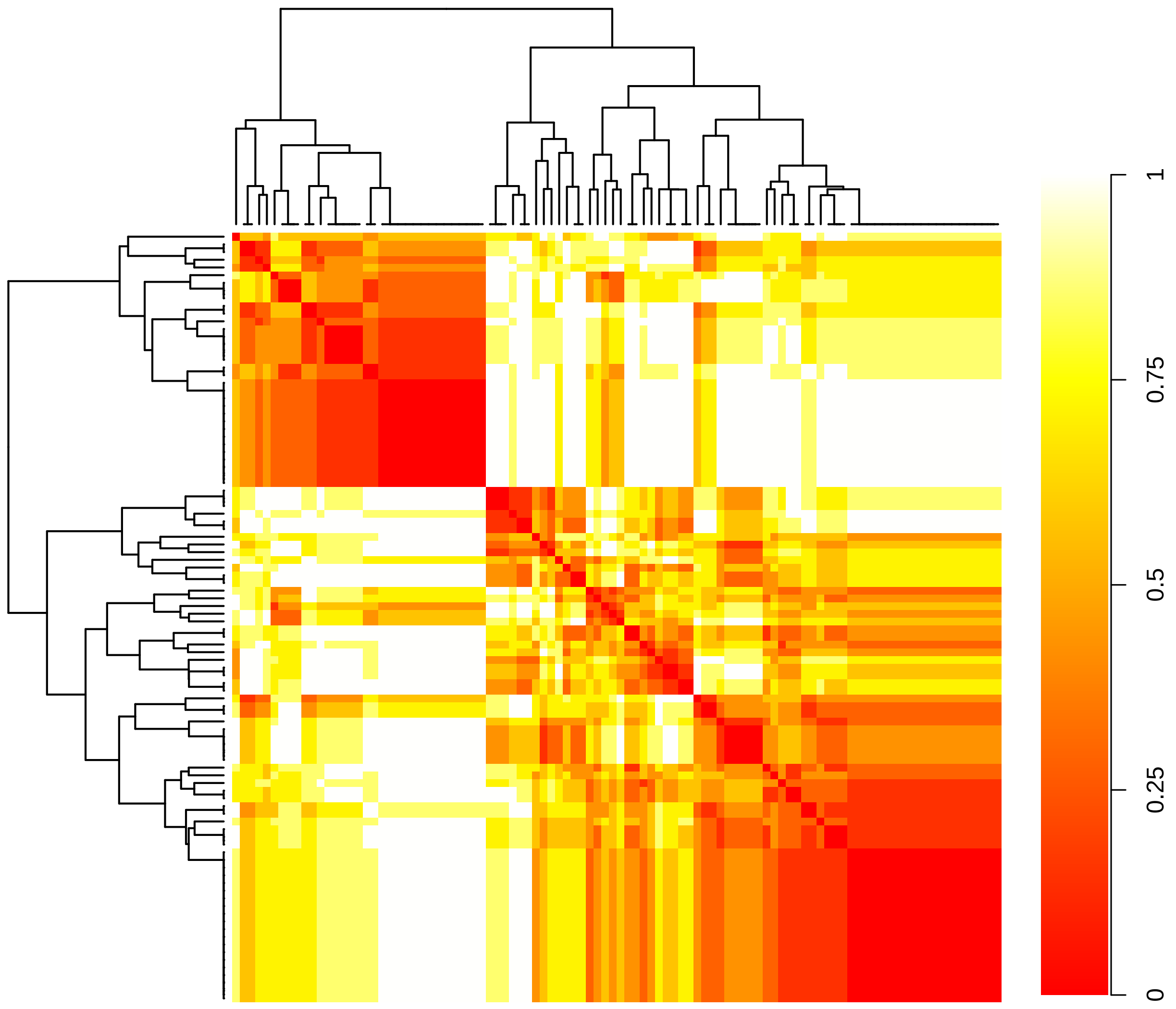

Figure 42: Pairwise secondary structure dissimilarity (1-Q3) in Cluster 14 (random sample of 100 fragments).

15 Structural variation in cluster number 15

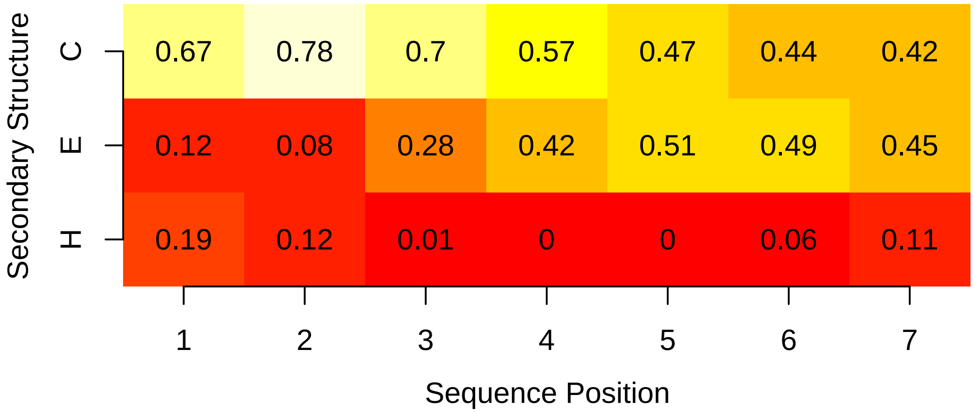

Figure 43: Position-specific secondary structure (STRIDE) in cluster 15.

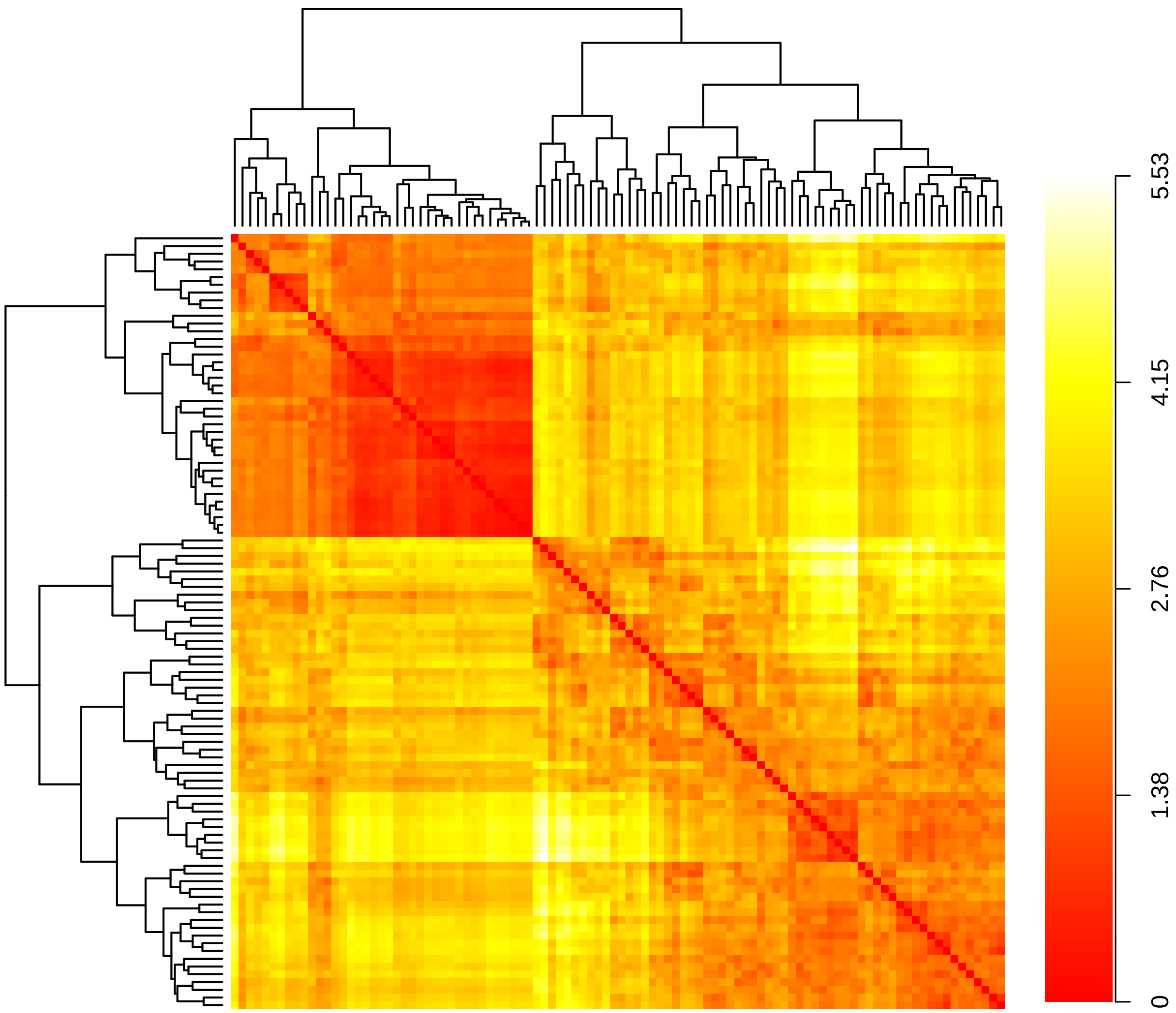

Figure 44: Pairwise RMSD in Cluster 15 (random sample of 100 fragments).

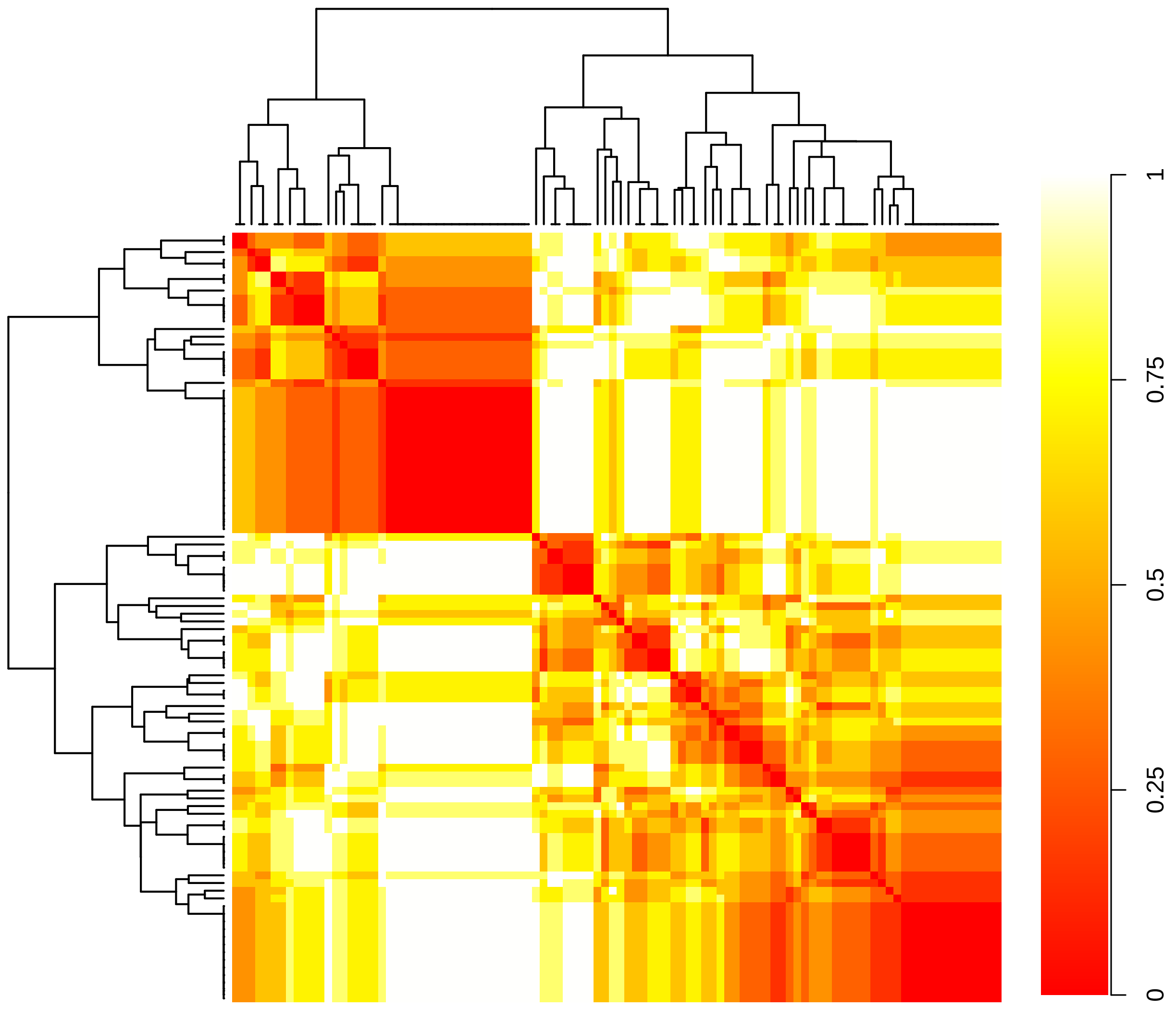

Figure 45: Pairwise secondary structure dissimilarity (1-Q3) in Cluster 15 (random sample of 100 fragments).

16 Structural variation in cluster number 16

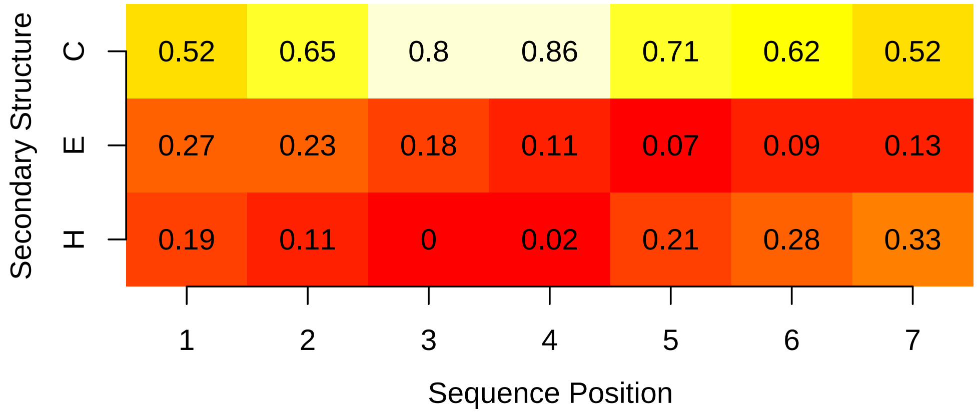

Figure 46: Position-specific secondary structure (STRIDE) in cluster 16.

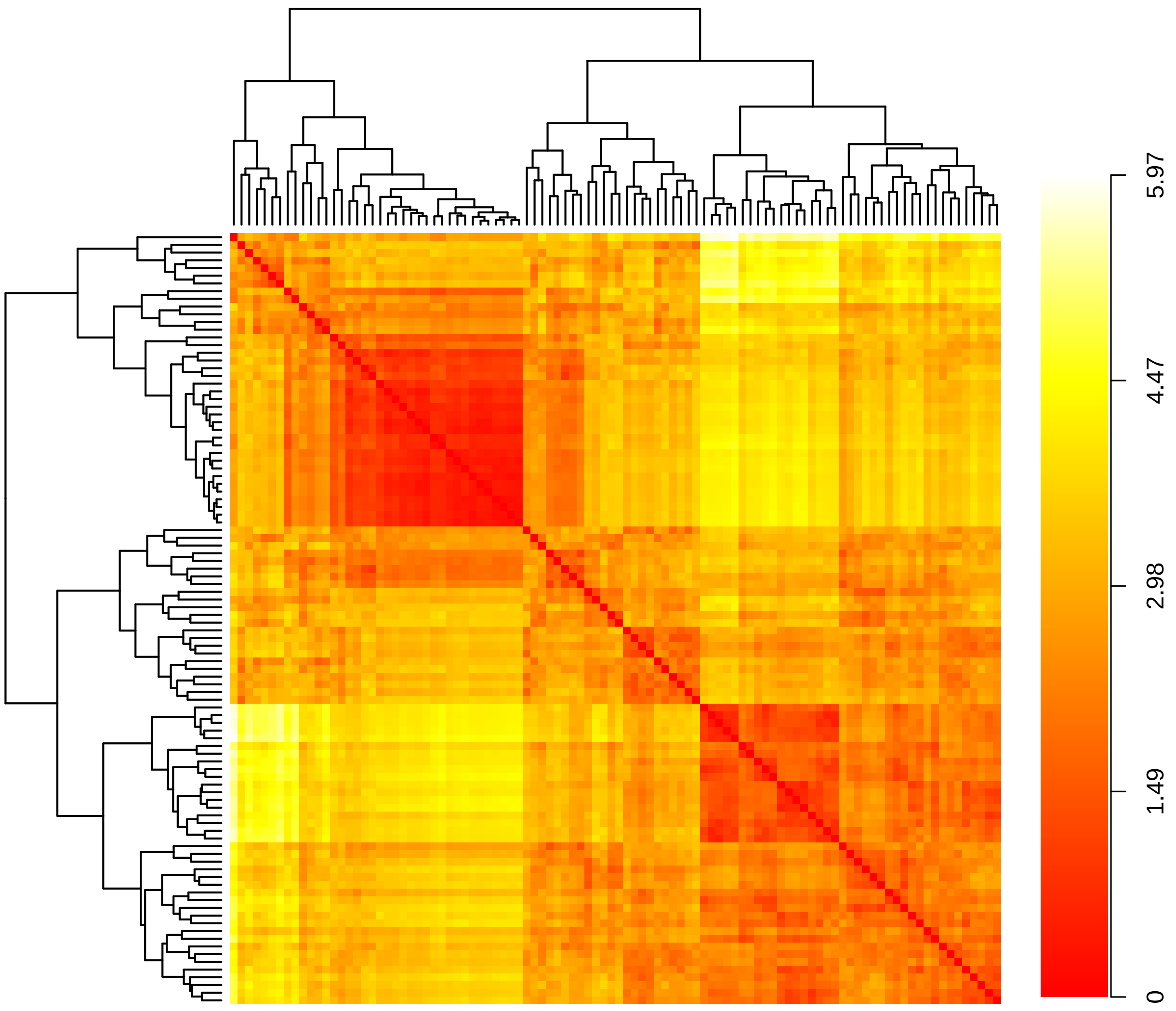

Figure 47: Pairwise RMSD in Cluster 16 (random sample of 100 fragments).

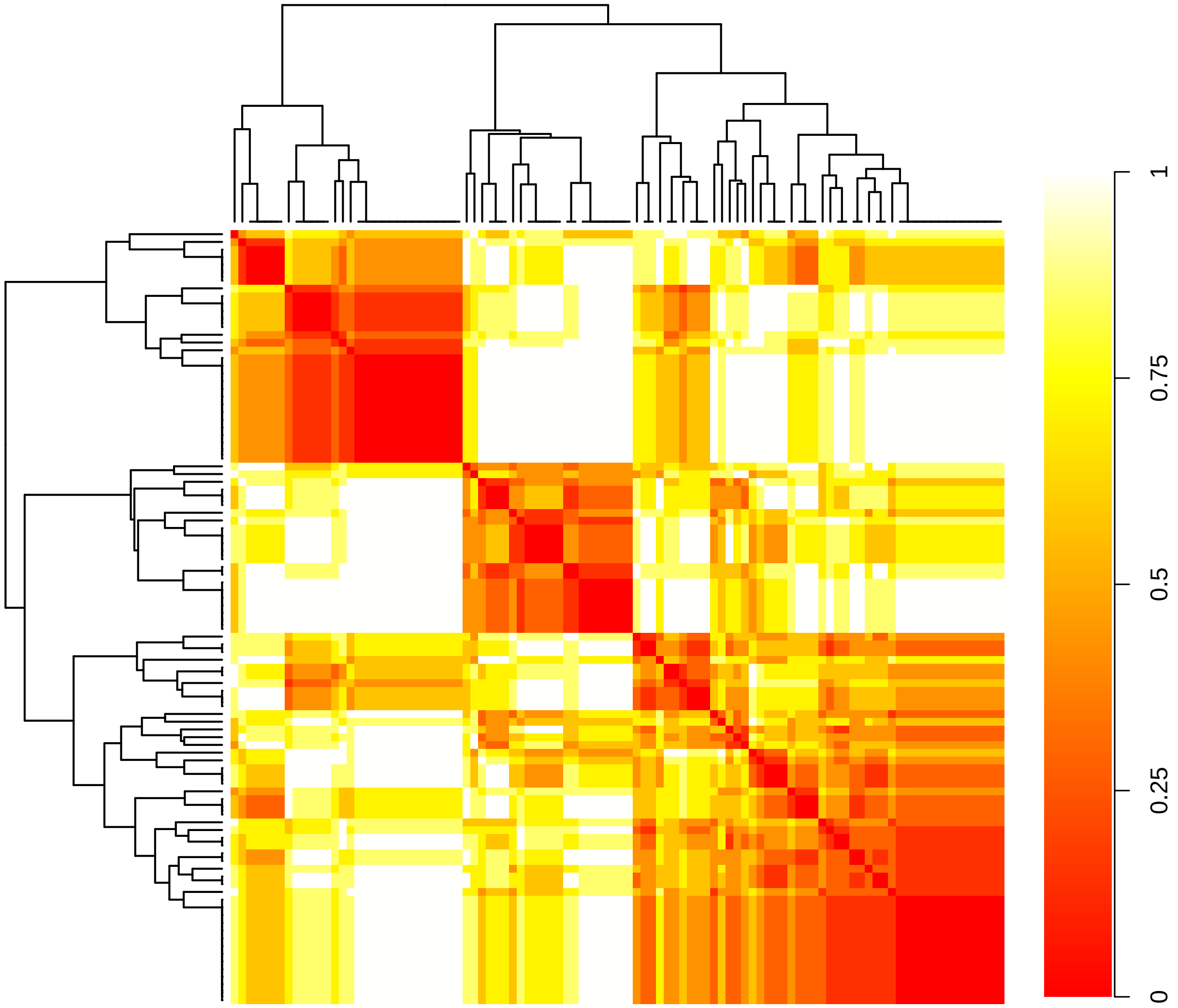

Figure 48: Pairwise secondary structure dissimilarity (1-Q3) in Cluster 16 (random sample of 100 fragments).

17 Structural variation in cluster number 17

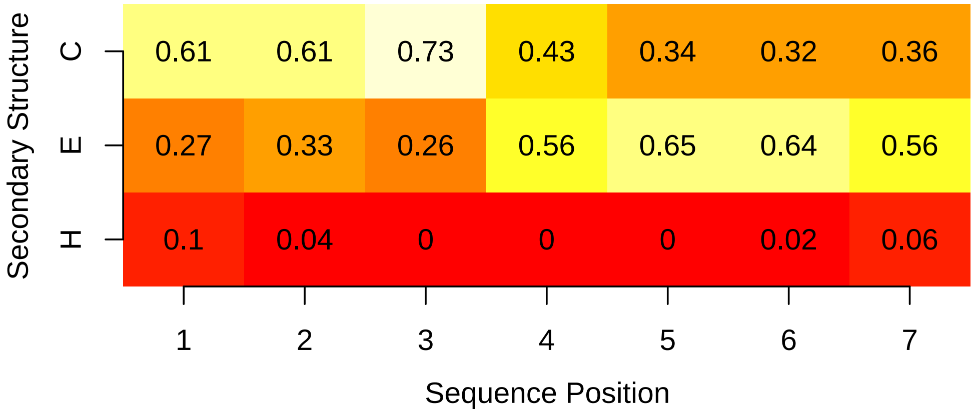

Figure 49: Position-specific secondary structure (STRIDE) in cluster 17.

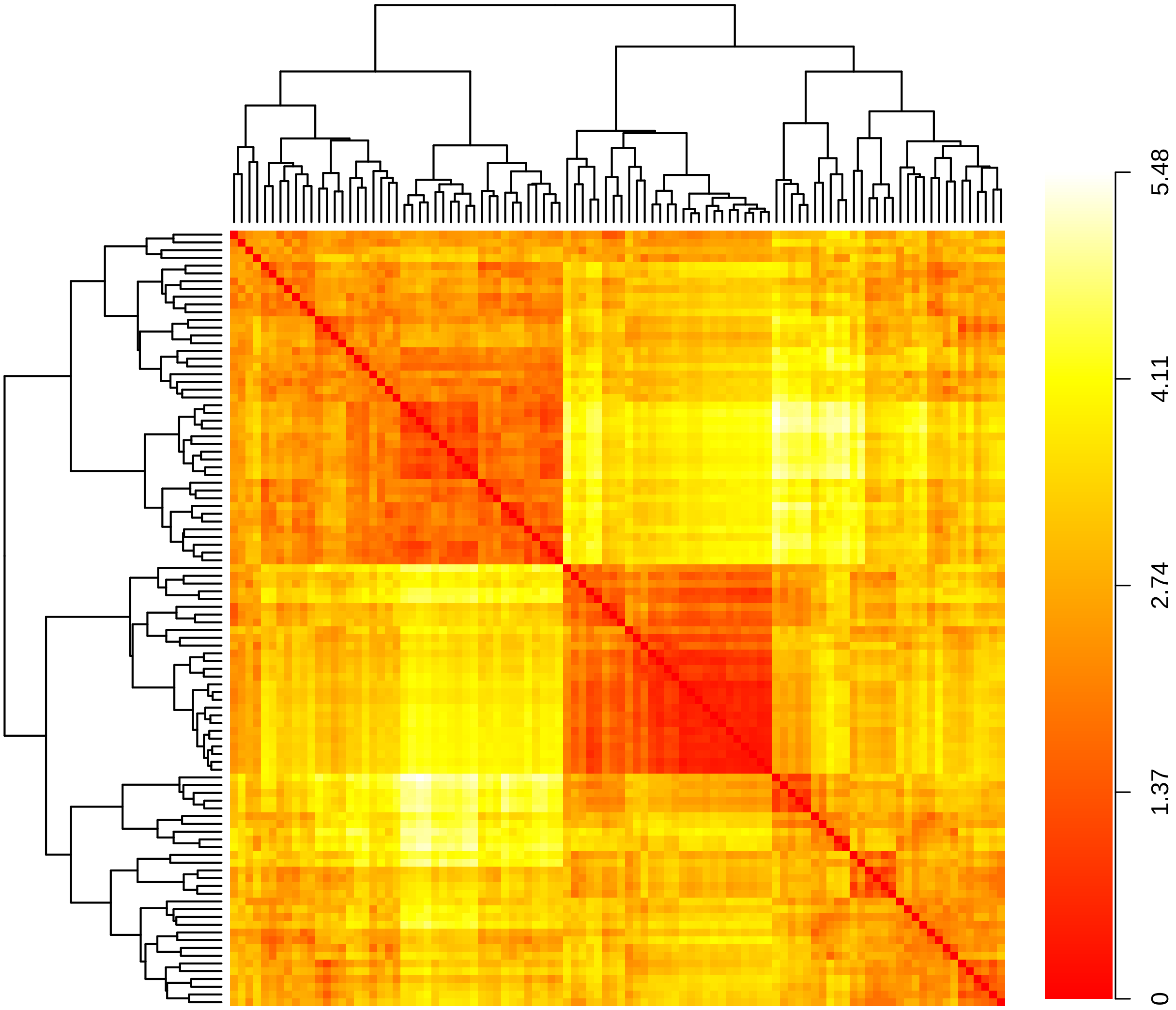

Figure 50: Pairwise RMSD in Cluster 17 (random sample of 100 fragments).

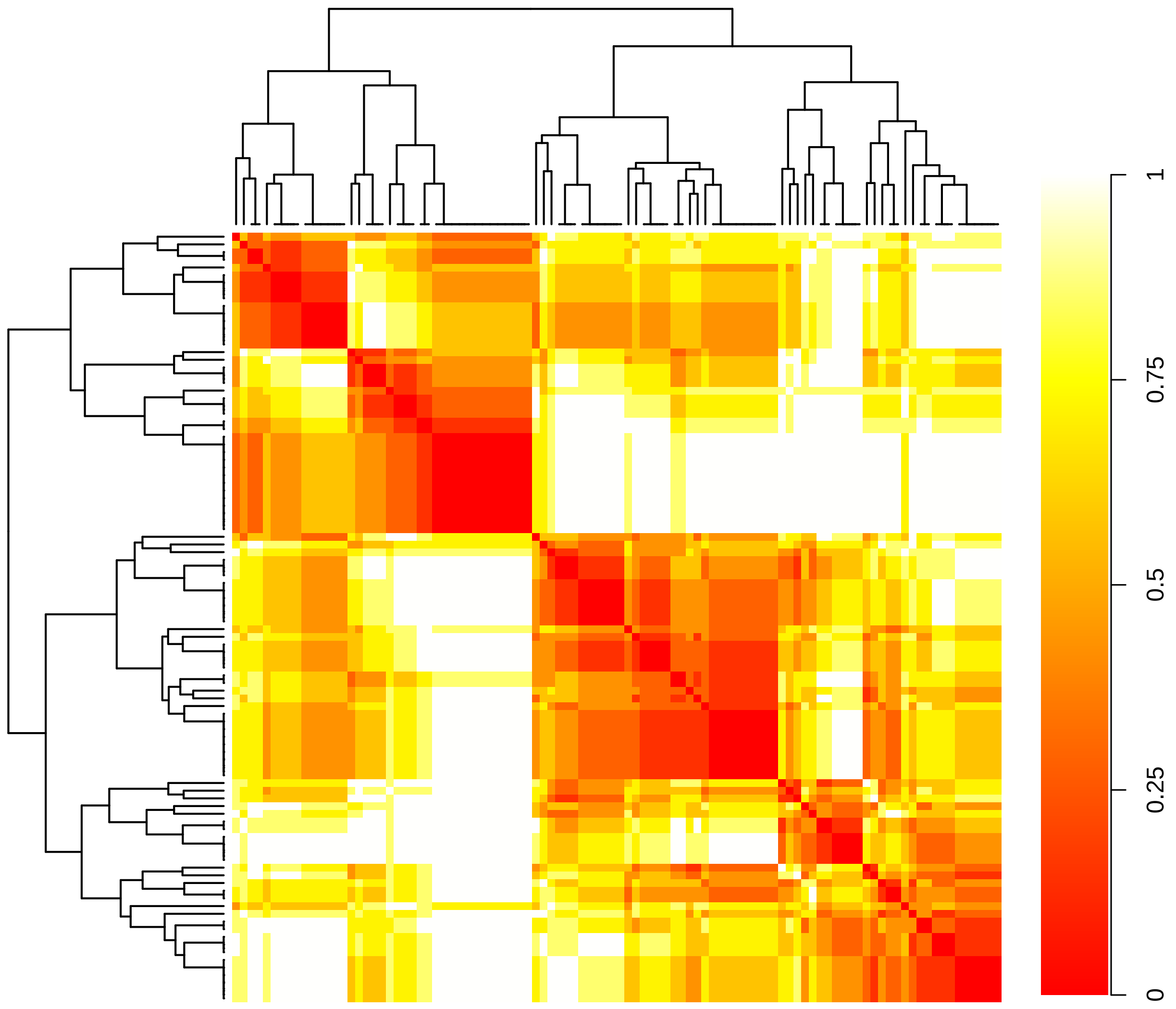

Figure 51: Pairwise secondary structure dissimilarity (1-Q3) in Cluster 17 (random sample of 100 fragments).

18 Structural variation in cluster number 18

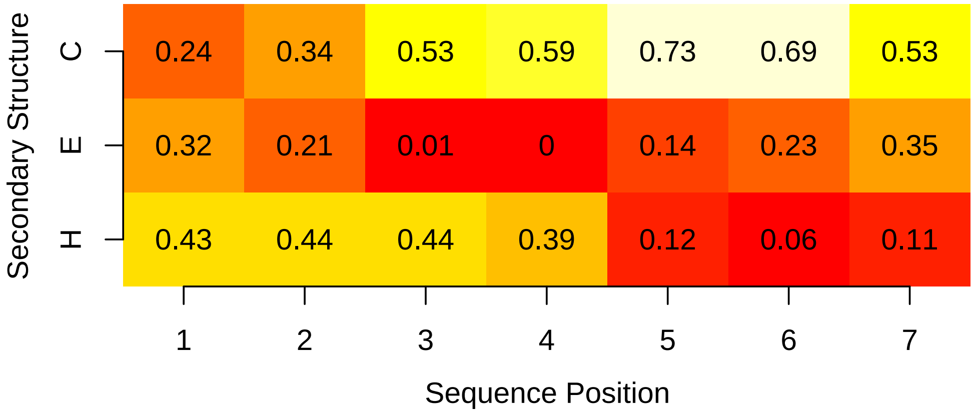

Figure 52: Position-specific secondary structure (STRIDE) in cluster 18.

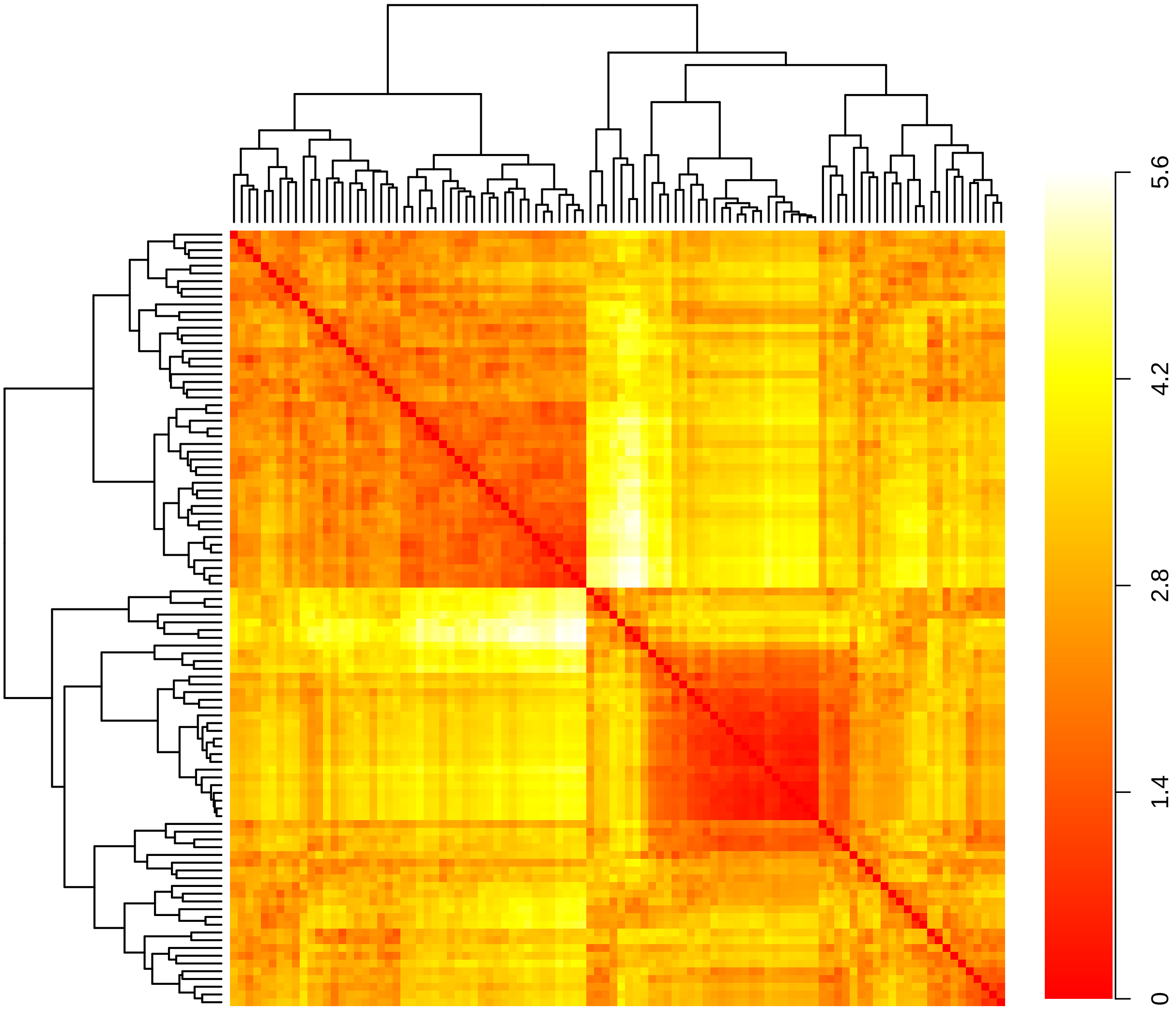

Figure 53: Pairwise RMSD in Cluster 18 (random sample of 100 fragments).

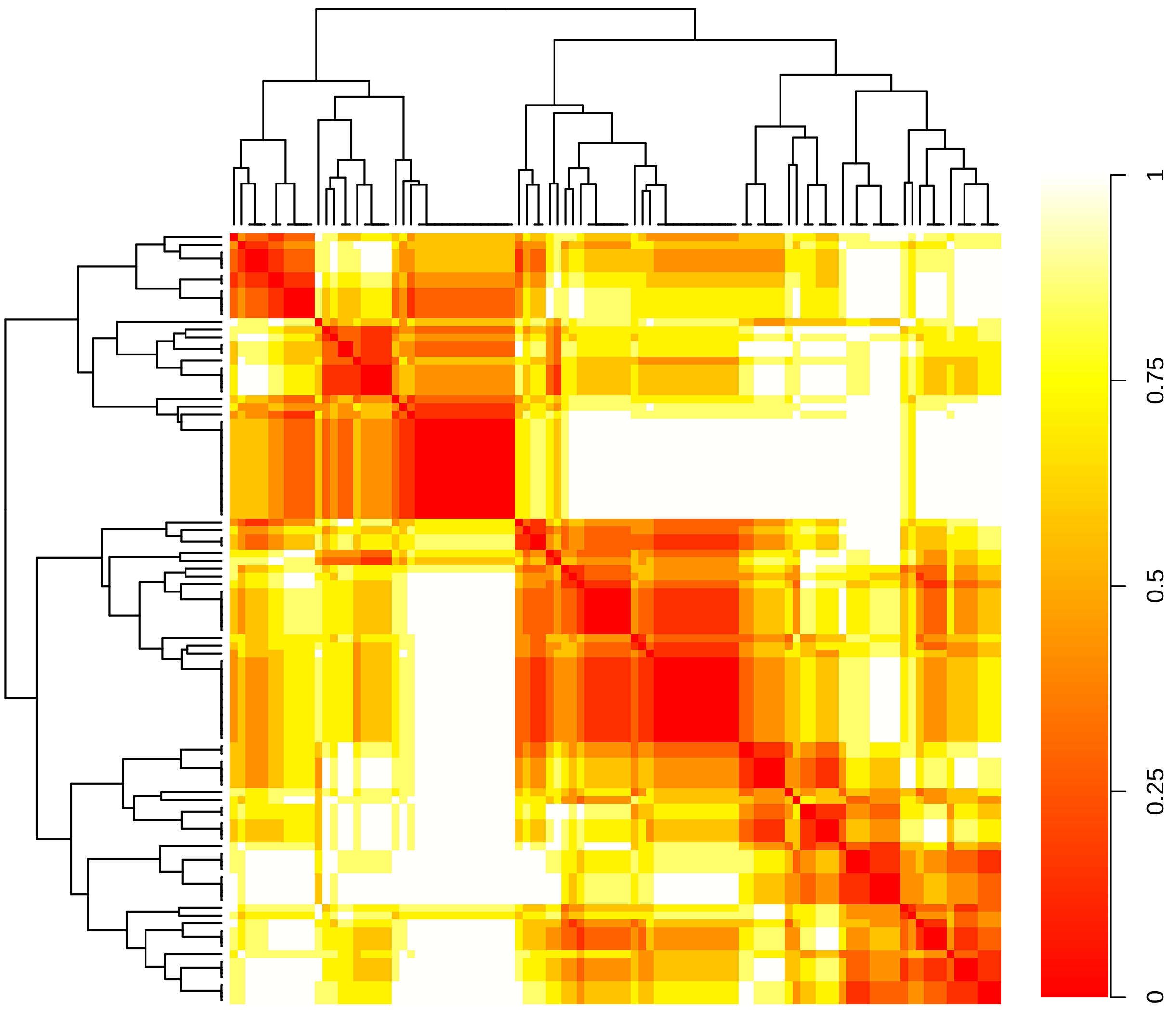

Figure 54: Pairwise secondary structure dissimilarity (1-Q3) in Cluster 18 (random sample of 100 fragments).

19 Structural variation in cluster number 19

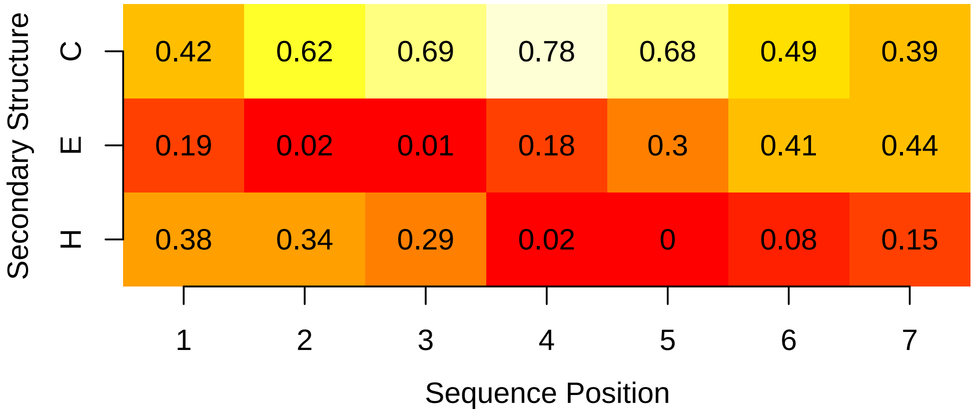

Figure 55: Position-specific secondary structure (STRIDE) in cluster 19.

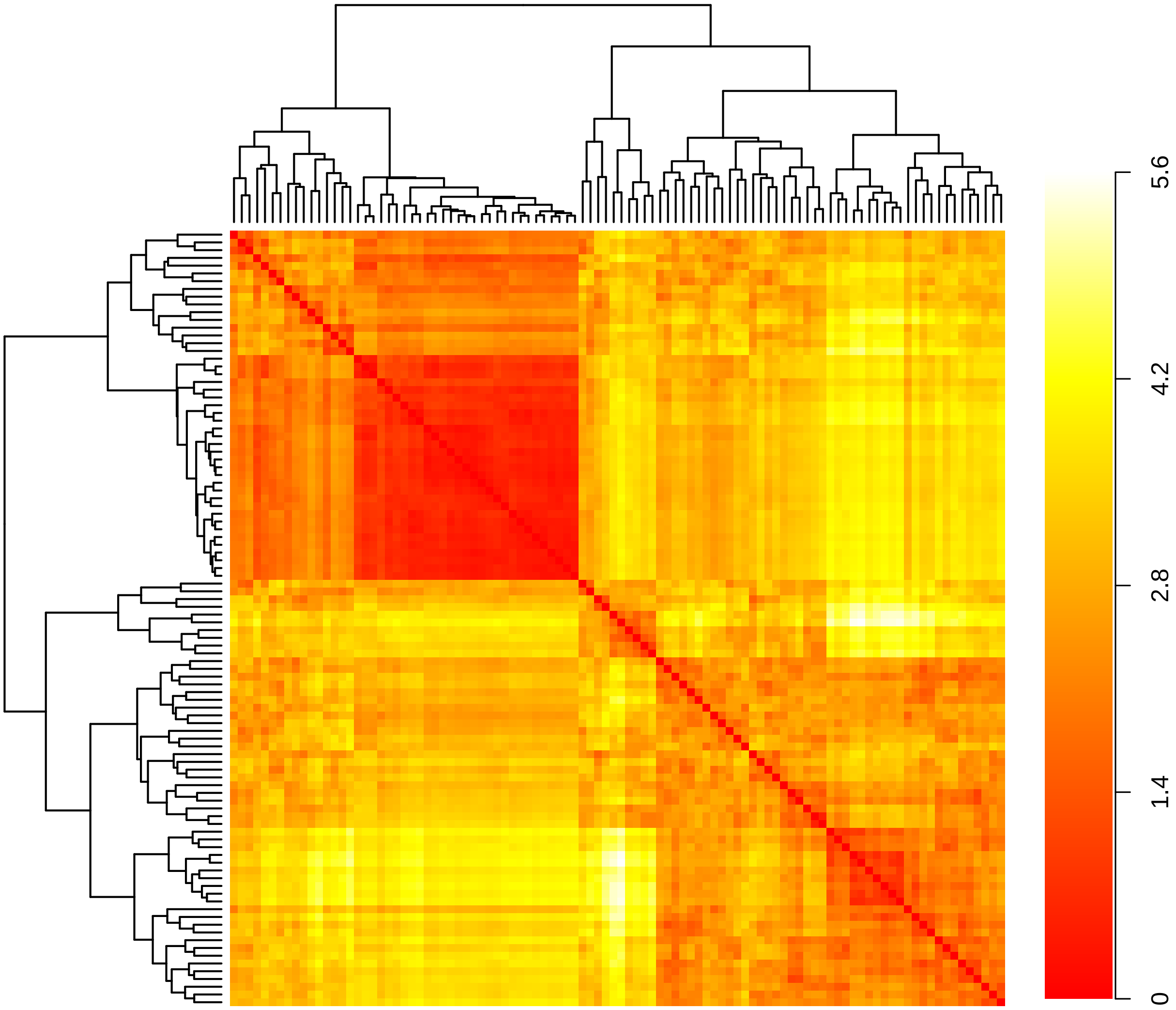

Figure 56: Pairwise RMSD in Cluster 19 (random sample of 100 fragments).

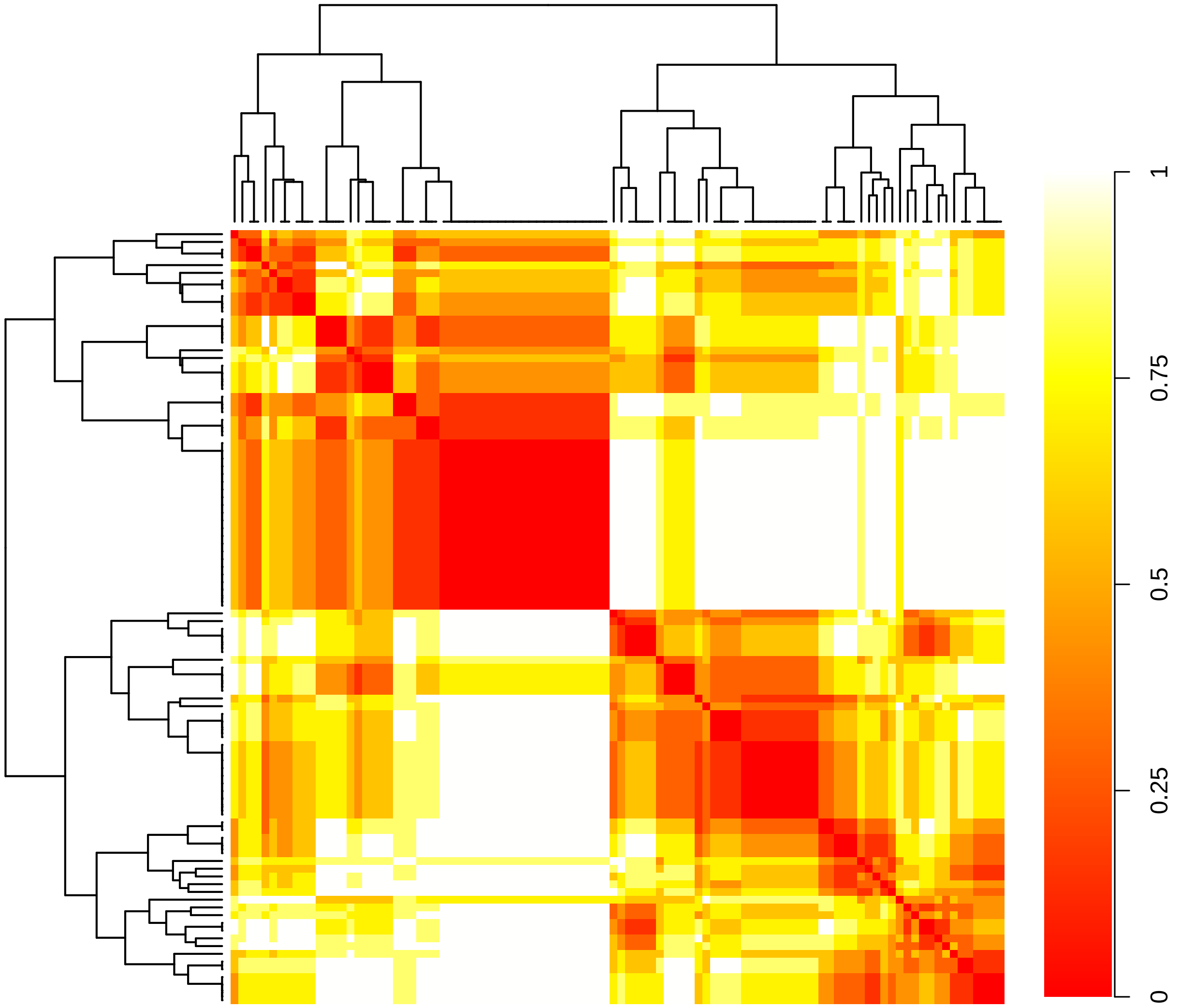

Figure 57: Pairwise secondary structure dissimilarity (1-Q3) in Cluster 19 (random sample of 100 fragments).

20 Structural variation in cluster number 20

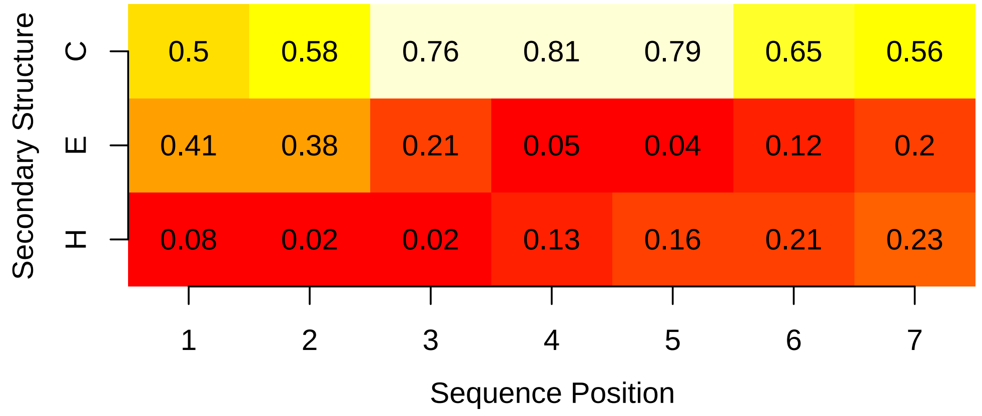

Figure 58: Position-specific secondary structure (STRIDE) in cluster 20.

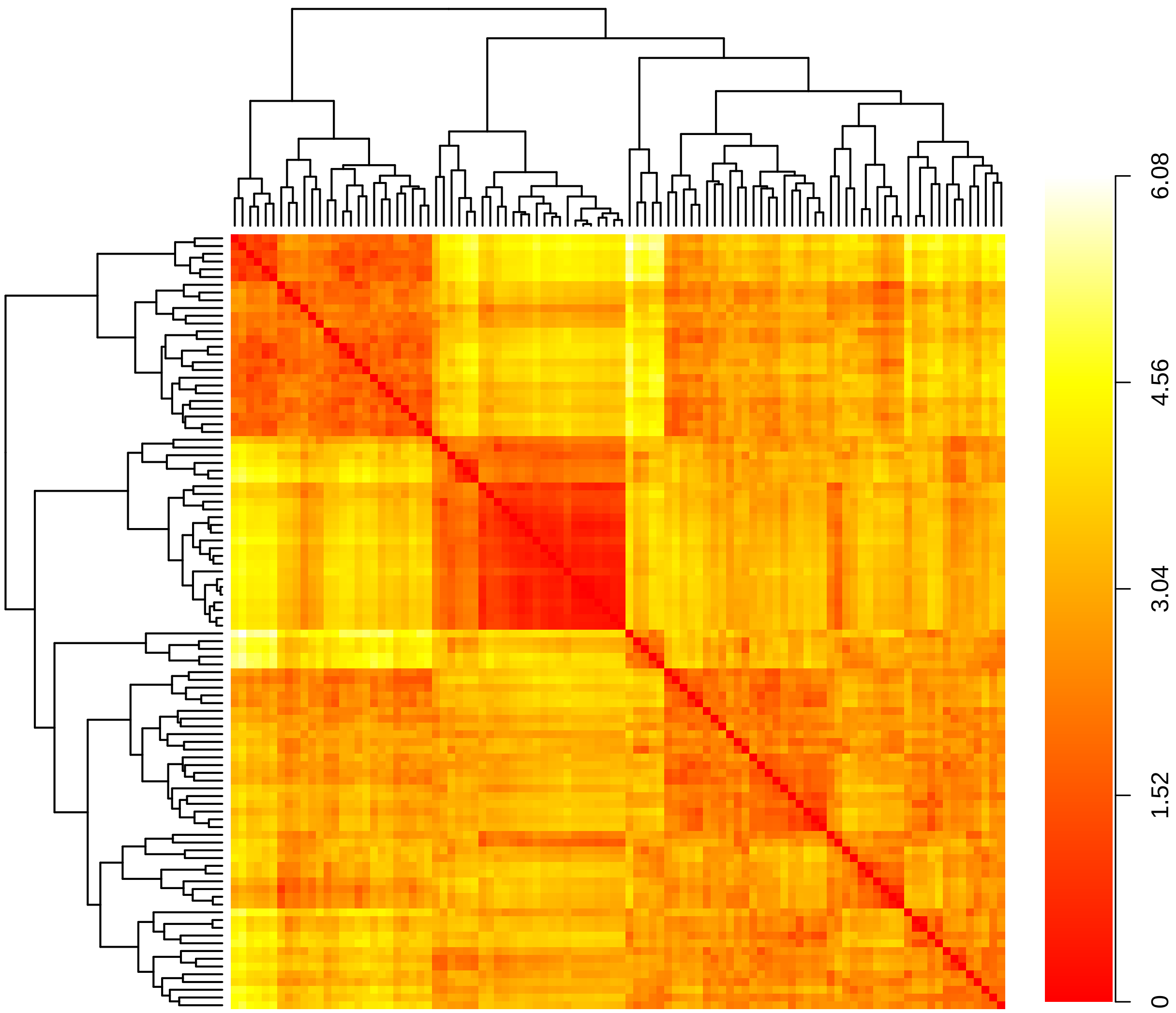

Figure 59: Pairwise RMSD in Cluster 20 (random sample of 100 fragments).

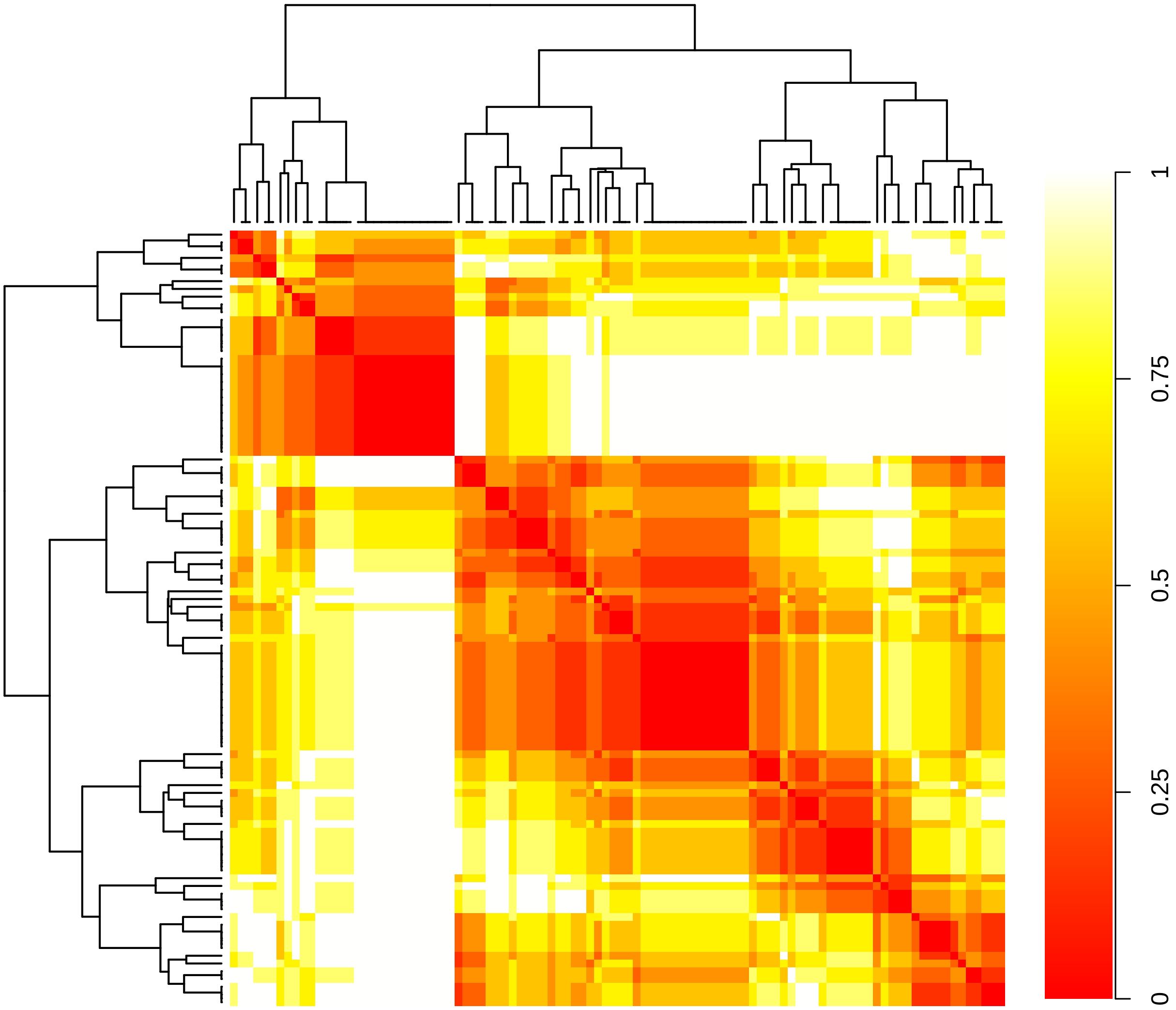

Figure 60: Pairwise secondary structure dissimilarity (1-Q3) in Cluster 20 (random sample of 100 fragments).

21 Structural variation in cluster number 21

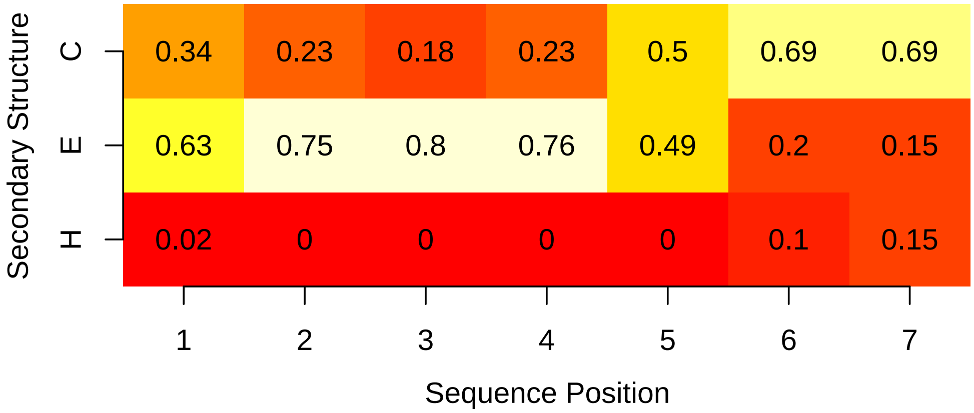

Figure 61: Position-specific secondary structure (STRIDE) in cluster 21.

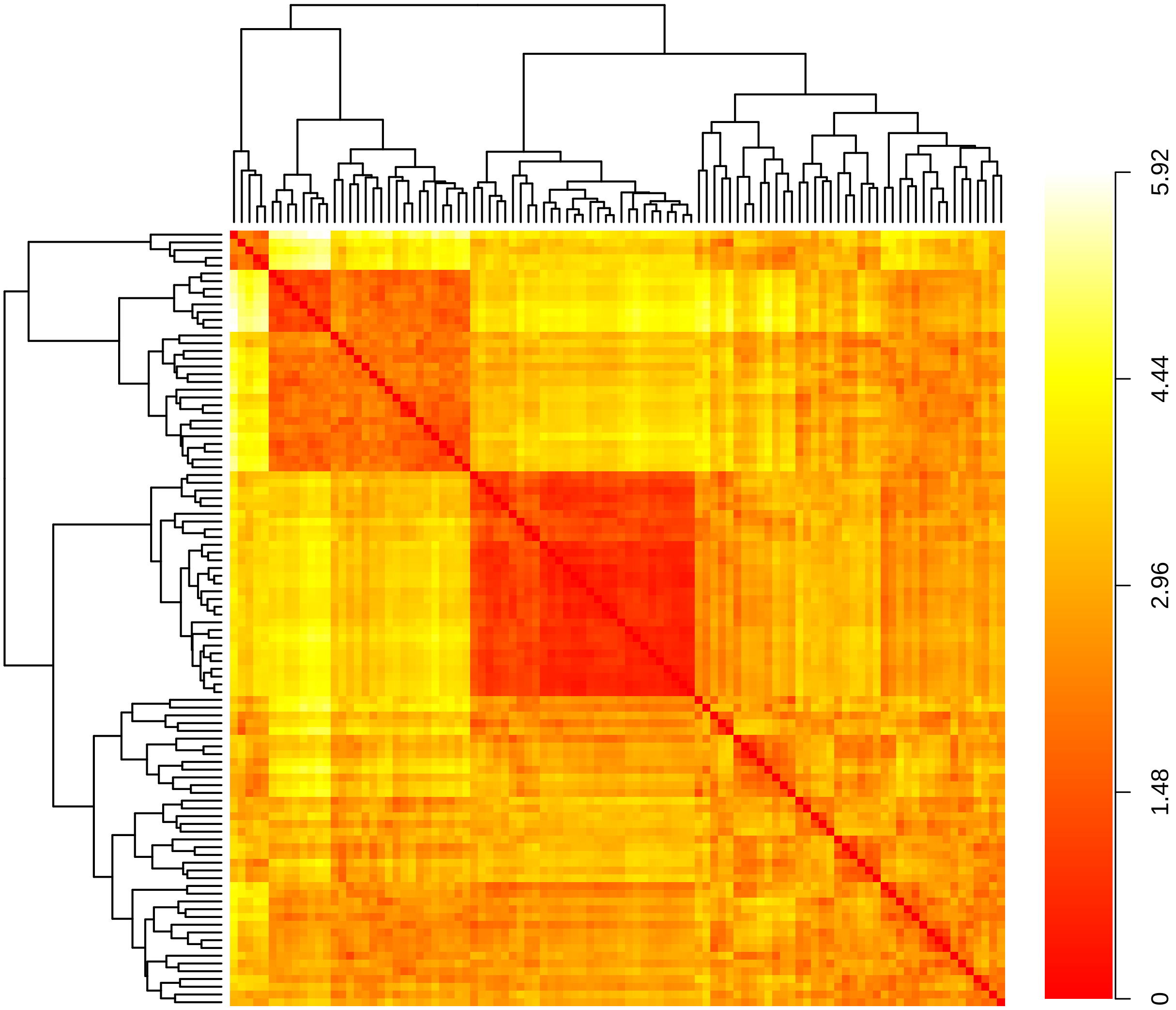

Figure 62: Pairwise RMSD in Cluster 21 (random sample of 100 fragments).

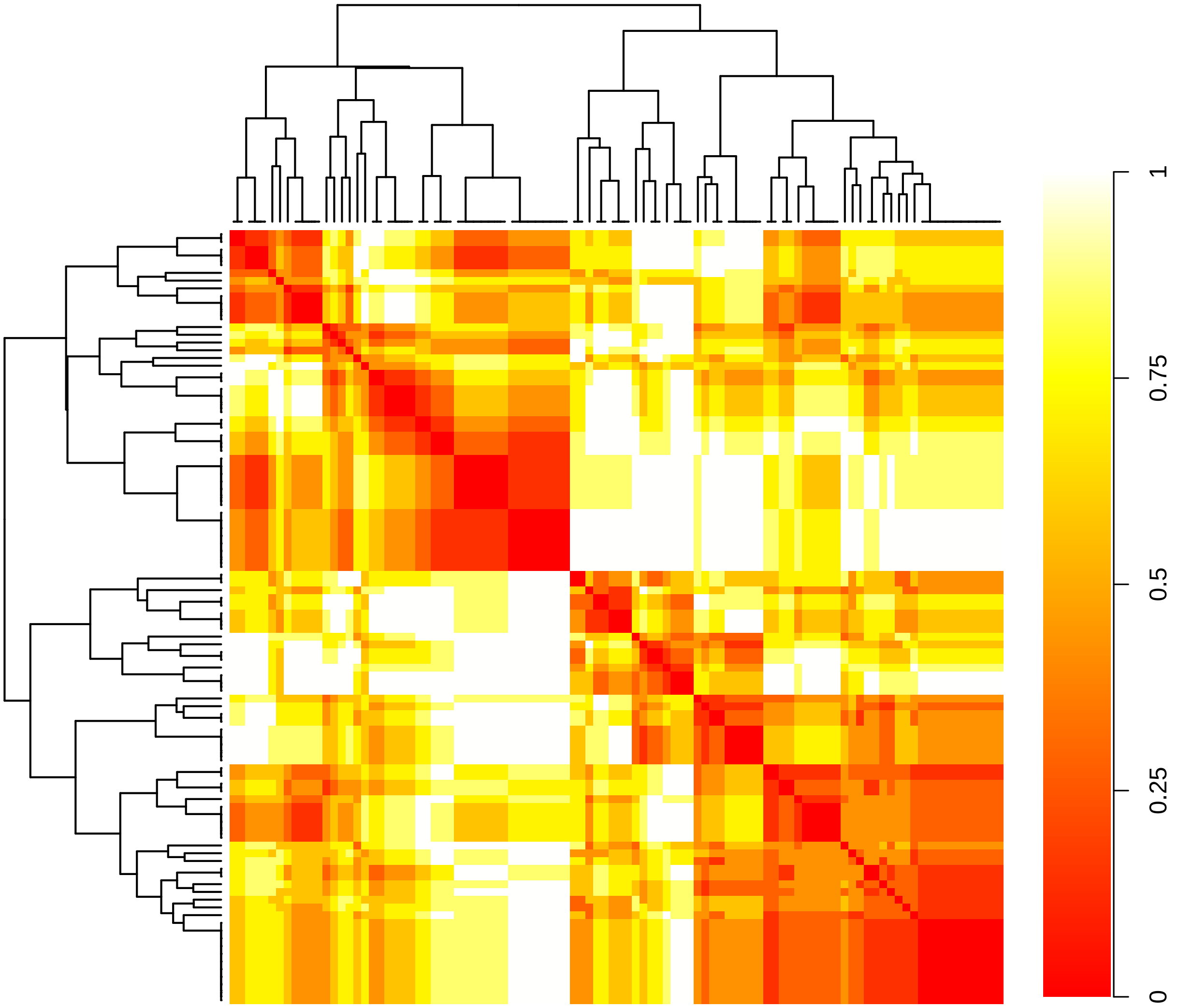

Figure 63: Pairwise secondary structure dissimilarity (1-Q3) in Cluster 21 (random sample of 100 fragments).

22 Structural variation in cluster number 22

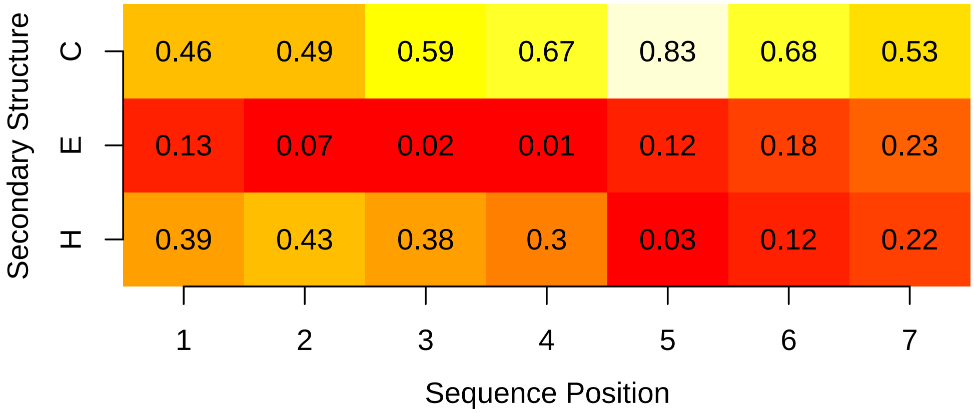

Figure 64: Position-specific secondary structure (STRIDE) in cluster 22.

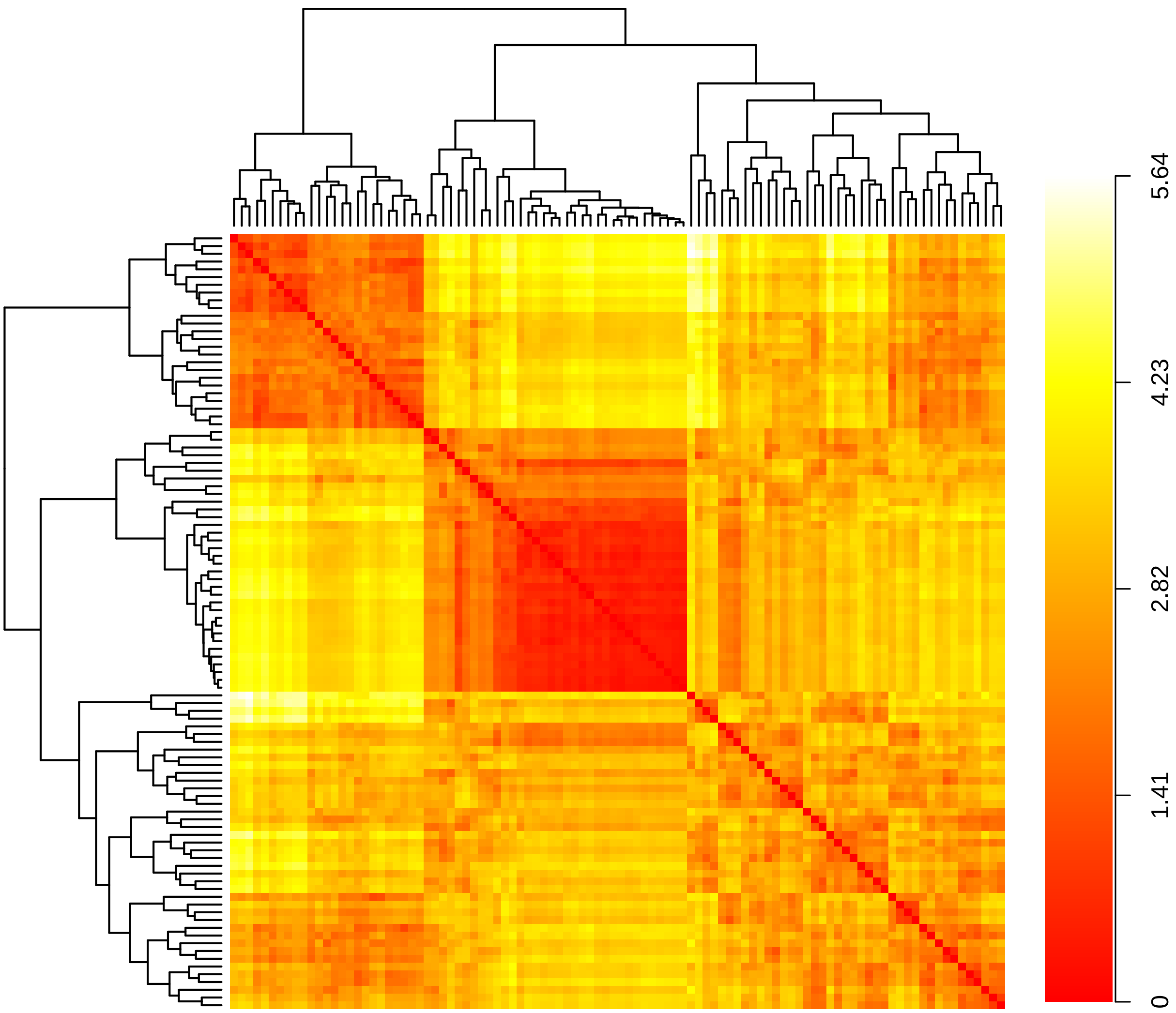

Figure 65: Pairwise RMSD in Cluster 22 (random sample of 100 fragments).

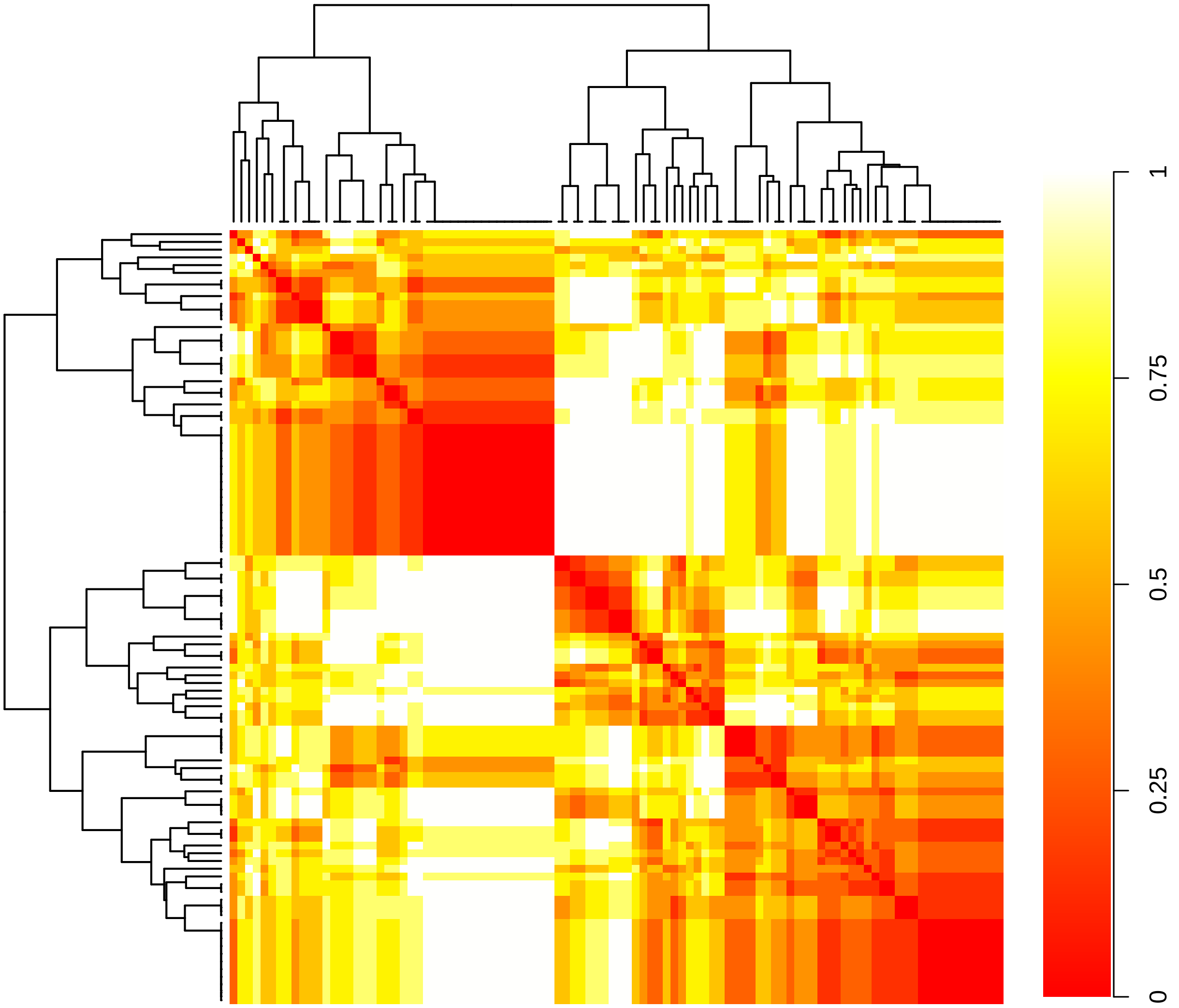

Figure 66: Pairwise secondary structure dissimilarity (1-Q3) in Cluster 22 (random sample of 100 fragments).

23 Structural variation in cluster number 23

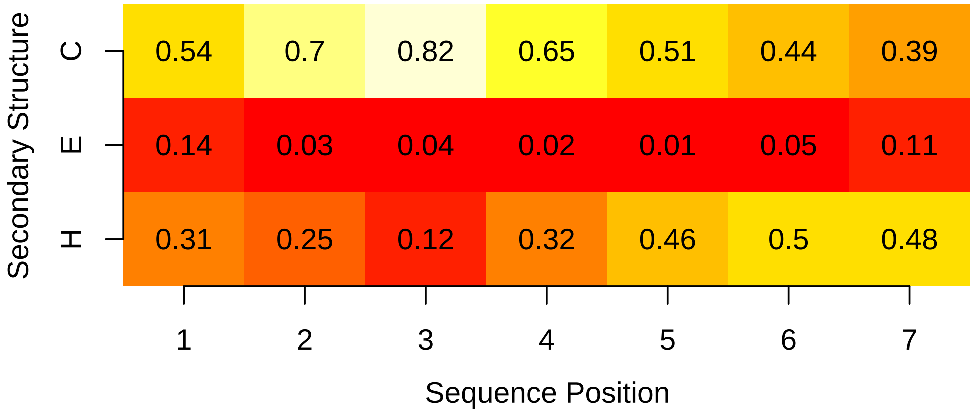

Figure 67: Position-specific secondary structure (STRIDE) in cluster 23.

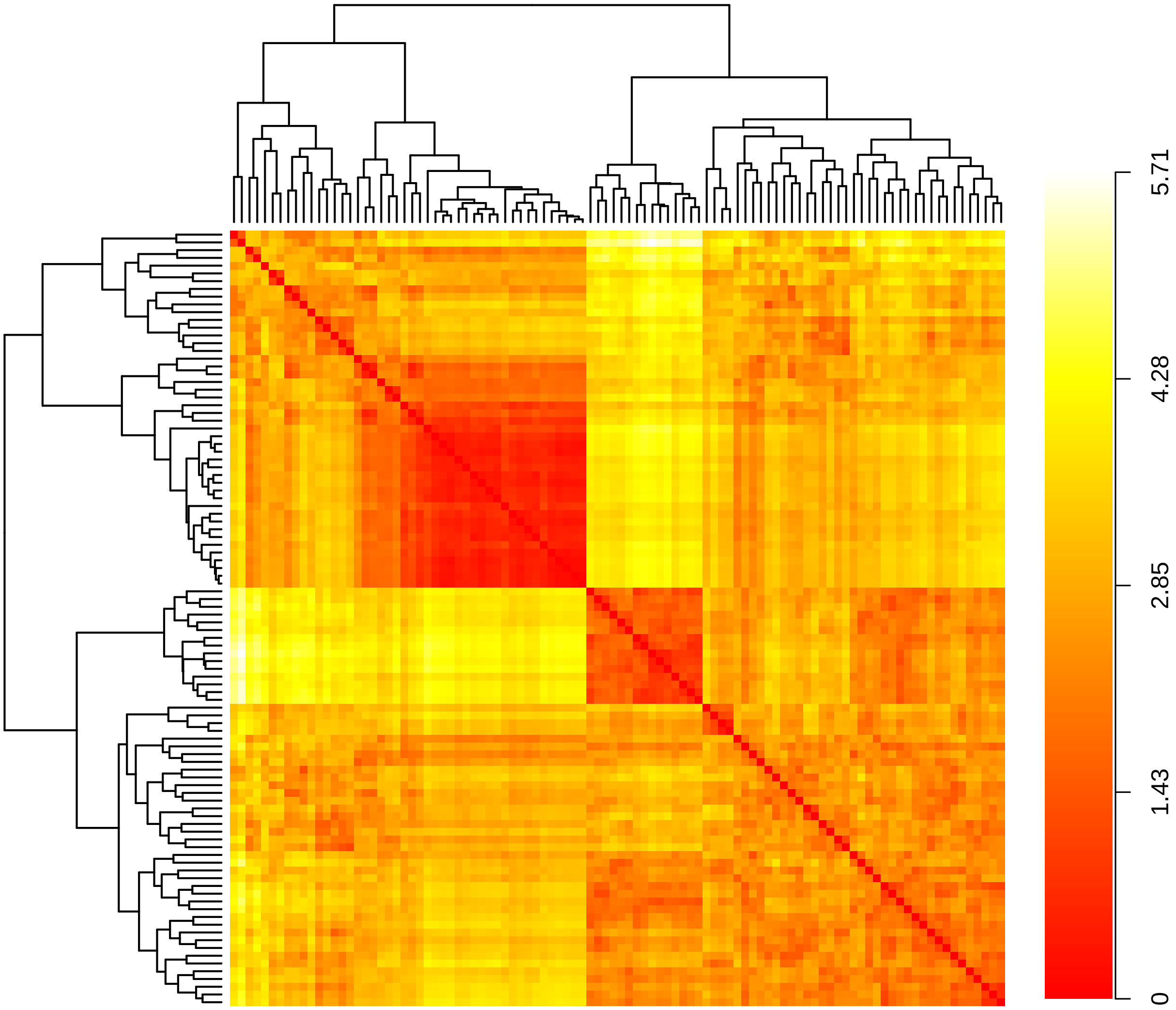

Figure 68: Pairwise RMSD in Cluster 23 (random sample of 100 fragments).

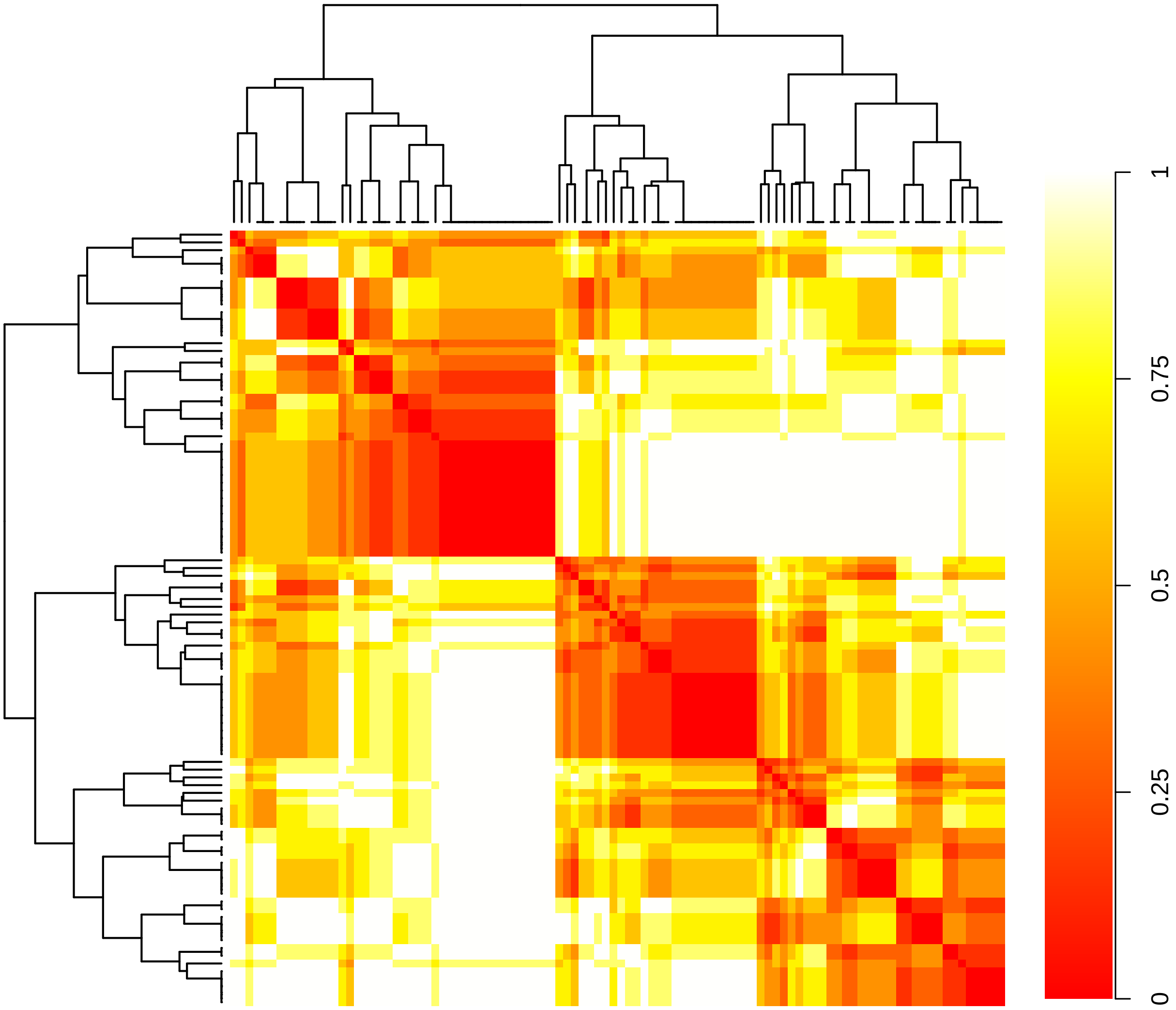

Figure 69: Pairwise secondary structure dissimilarity (1-Q3) in Cluster 23 (random sample of 100 fragments).

24 Structural variation in cluster number 24

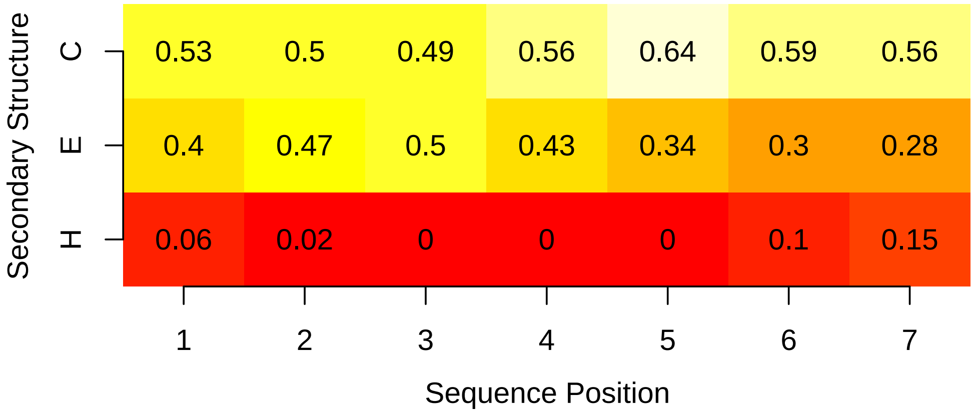

Figure 70: Position-specific secondary structure (STRIDE) in cluster 24.

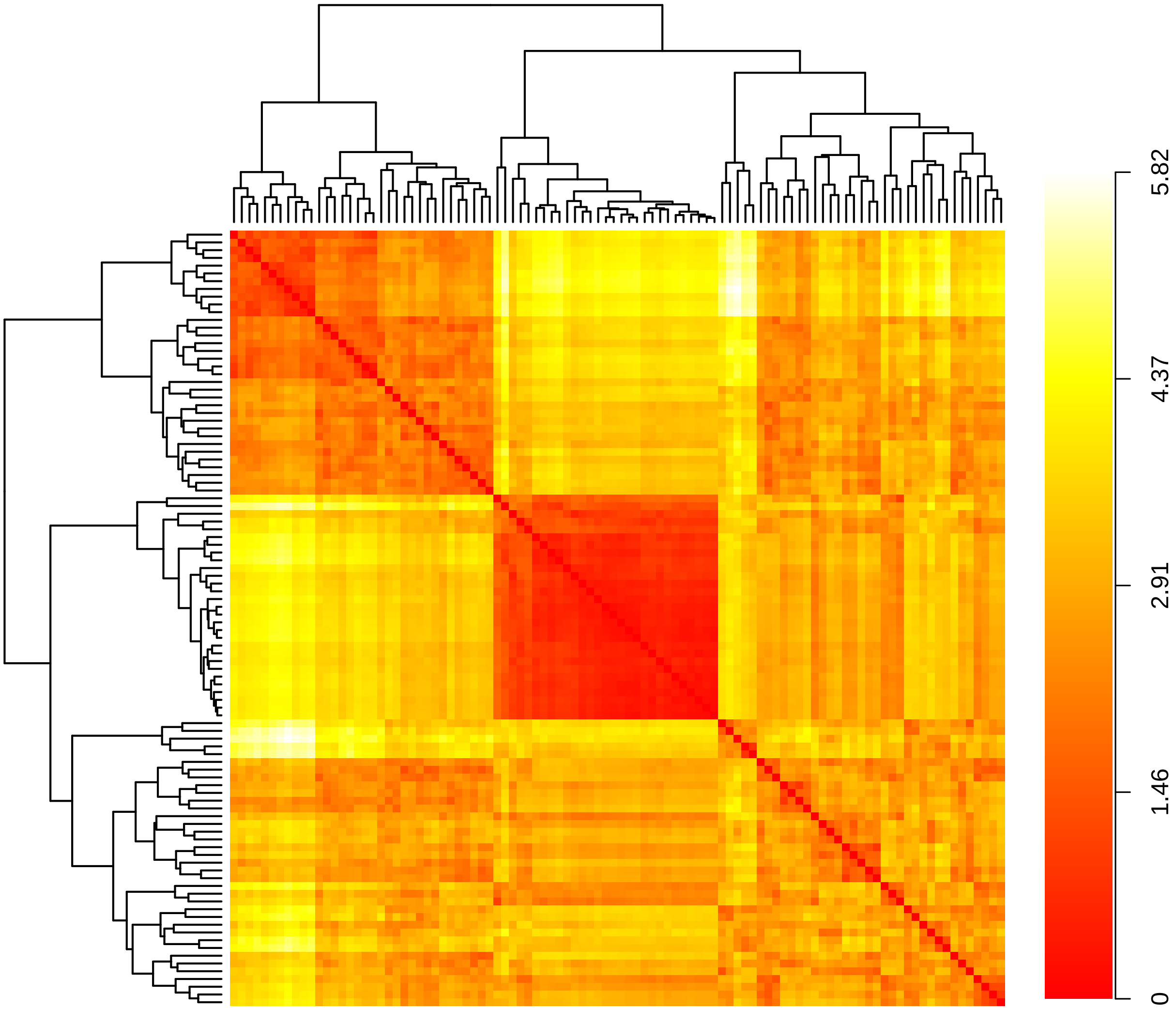

Figure 71: Pairwise RMSD in Cluster 24 (random sample of 100 fragments).

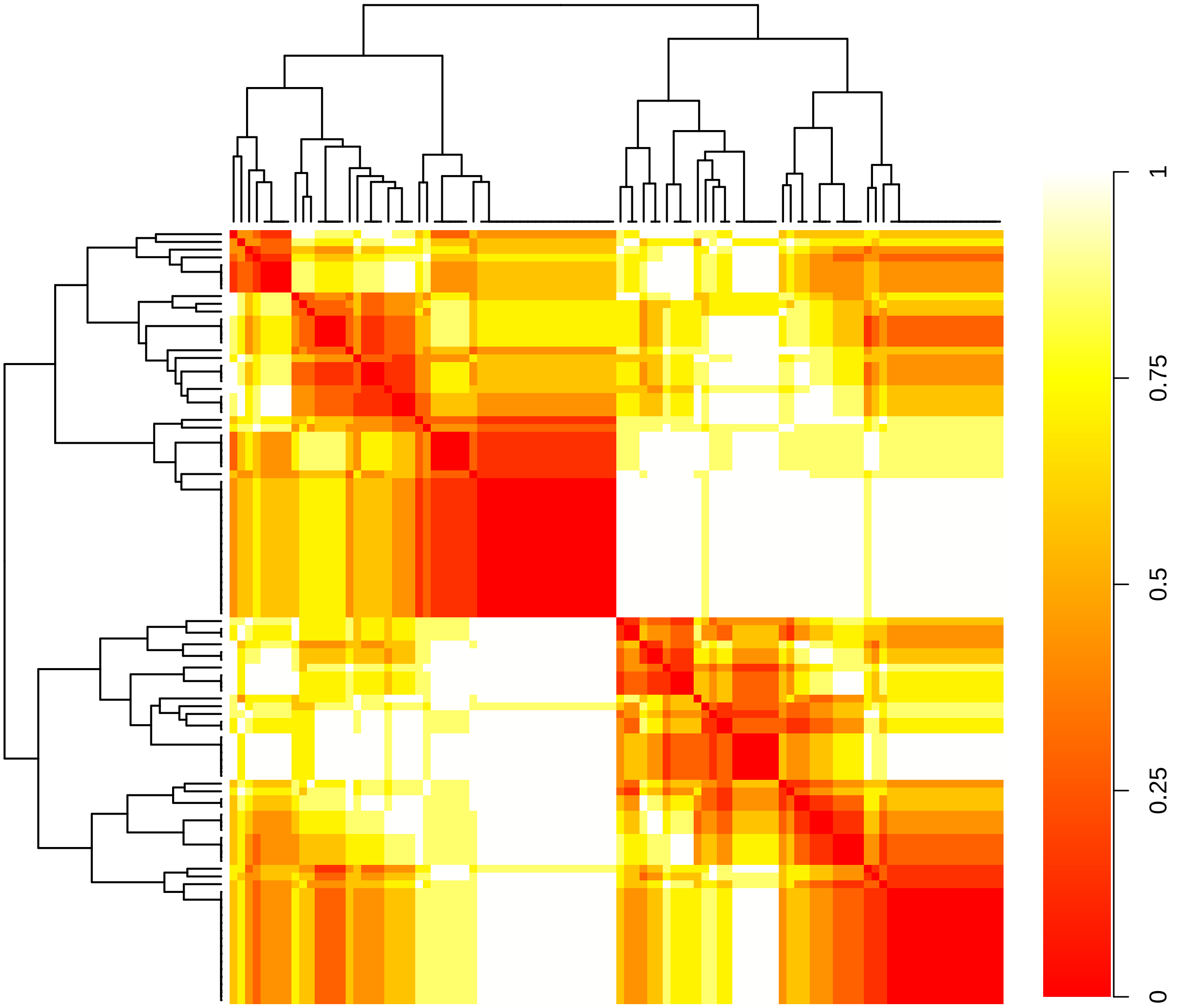

Figure 72: Pairwise secondary structure dissimilarity (1-Q3) in Cluster 24 (random sample of 100 fragments).

25 Structural variation in cluster number 25

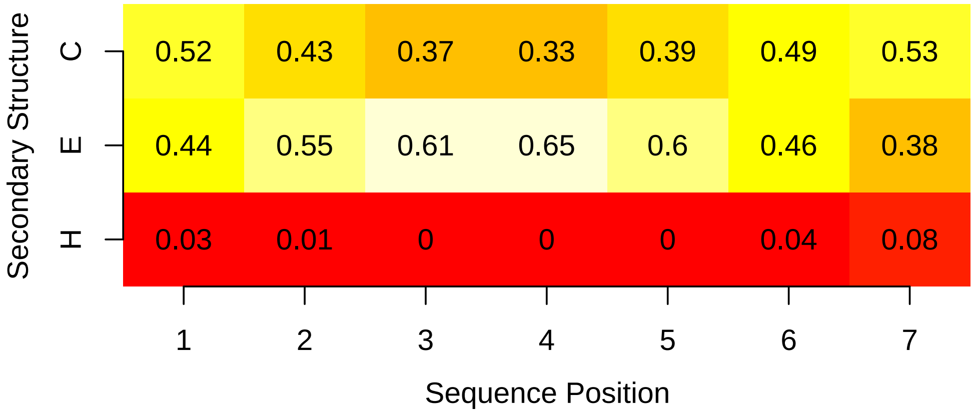

Figure 73: Position-specific secondary structure (STRIDE) in cluster 25.

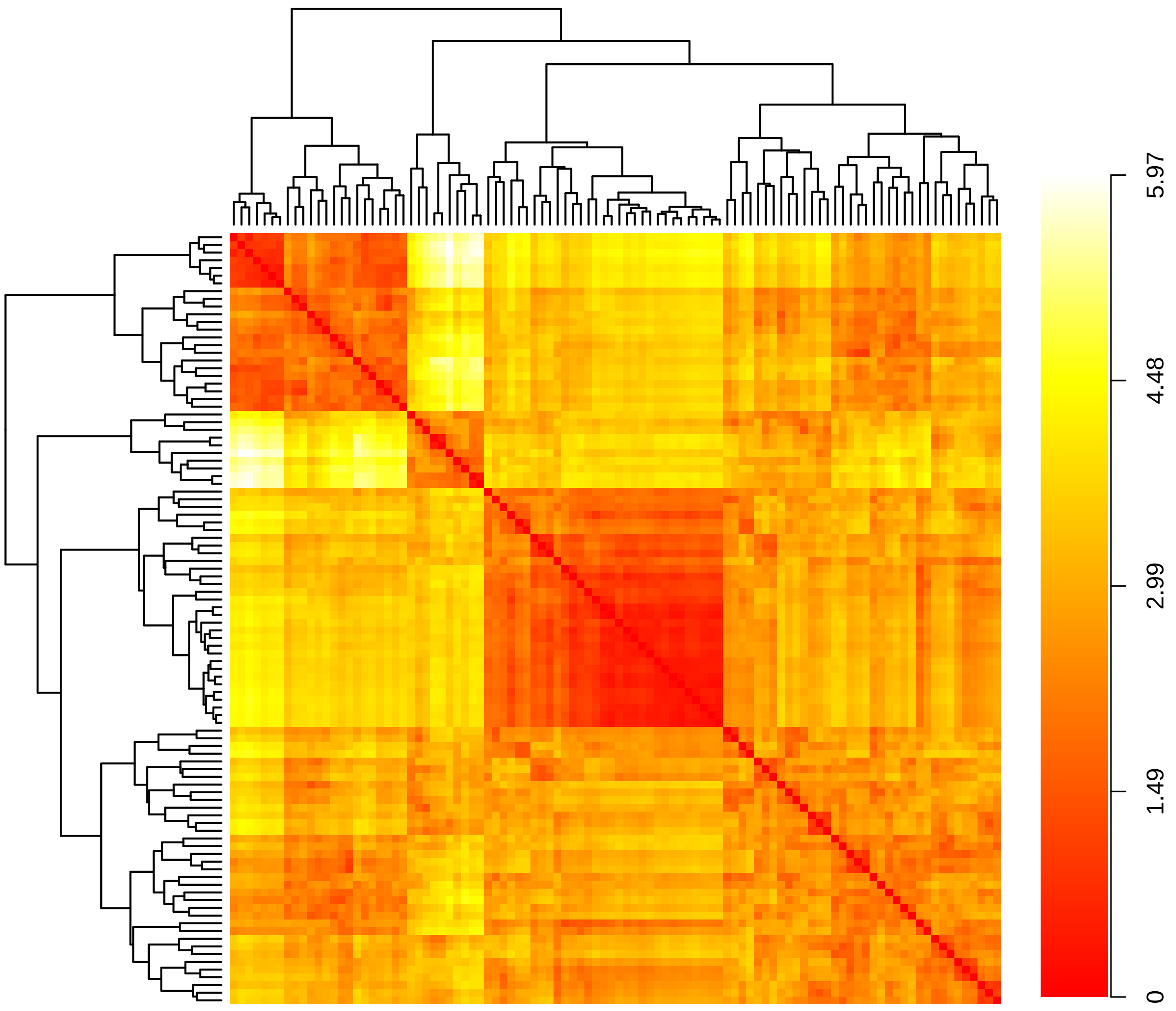

Figure 74: Pairwise RMSD in Cluster 25 (random sample of 100 fragments).

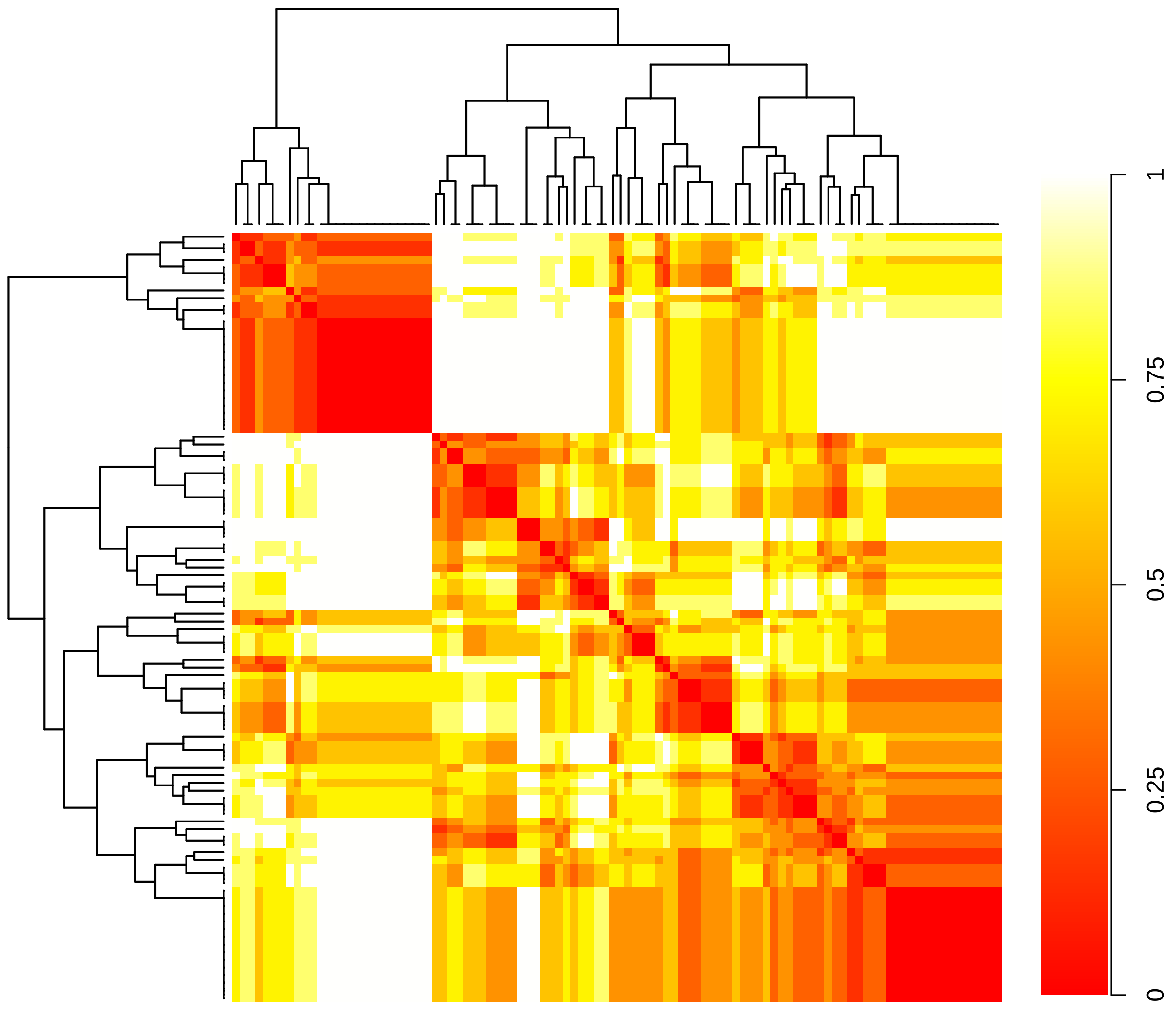

Figure 75: Pairwise secondary structure dissimilarity (1-Q3) in Cluster 25 (random sample of 100 fragments).

26 Structural variation in cluster number 26

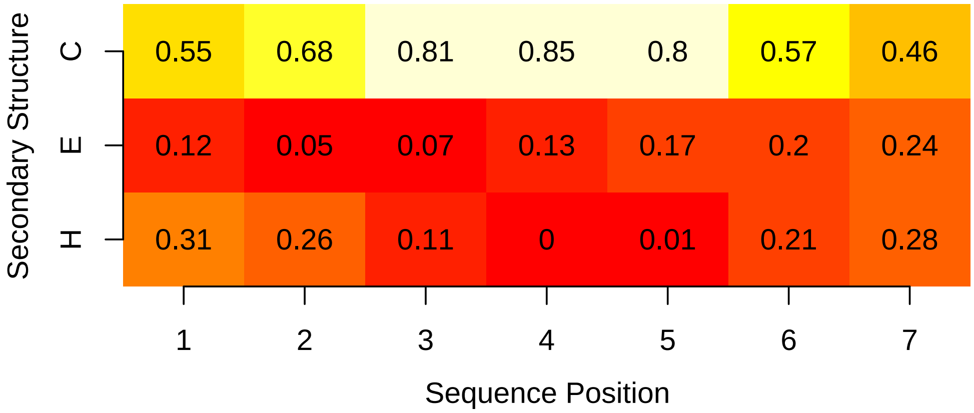

Figure 76: Position-specific secondary structure (STRIDE) in cluster 26.

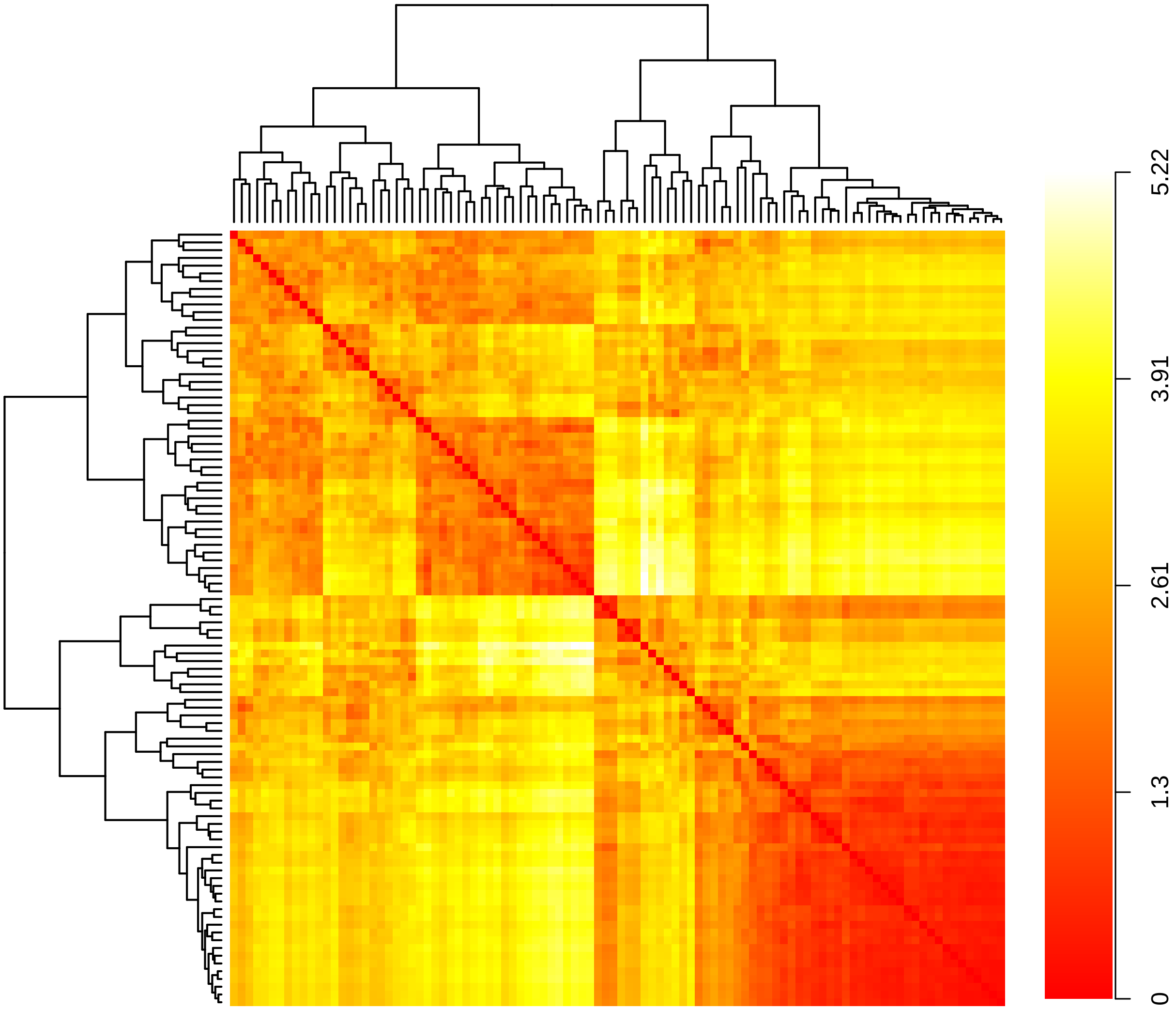

Figure 77: Pairwise RMSD in Cluster 26 (random sample of 100 fragments).

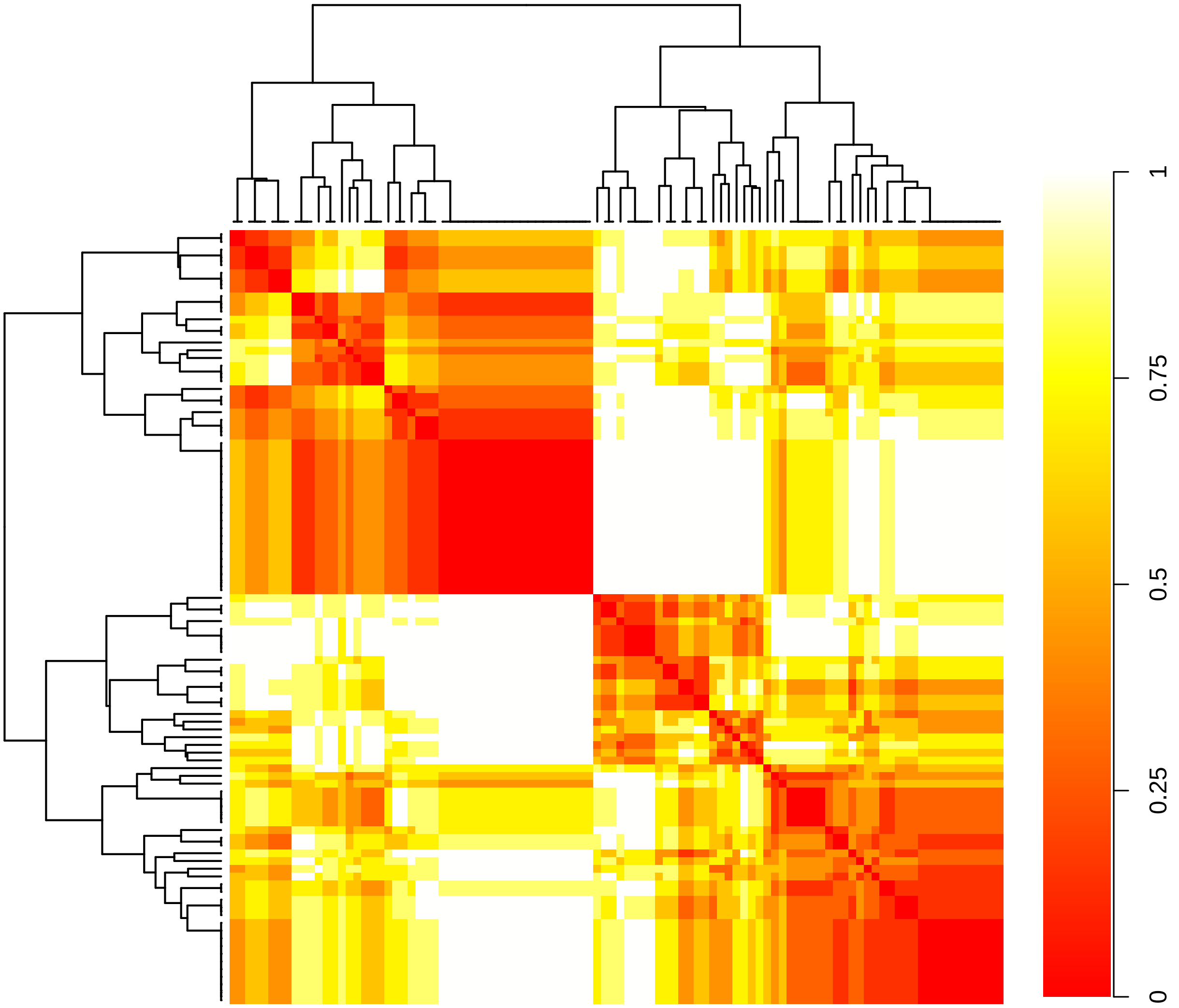

Figure 78: Pairwise secondary structure dissimilarity (1-Q3) in Cluster 26 (random sample of 100 fragments).

27 Structural variation in cluster number 27

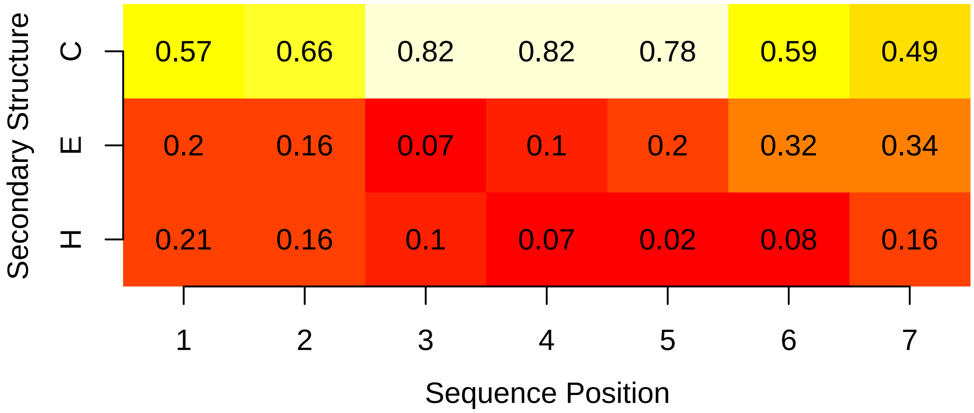

Figure 79: Position-specific secondary structure (STRIDE) in cluster 27.

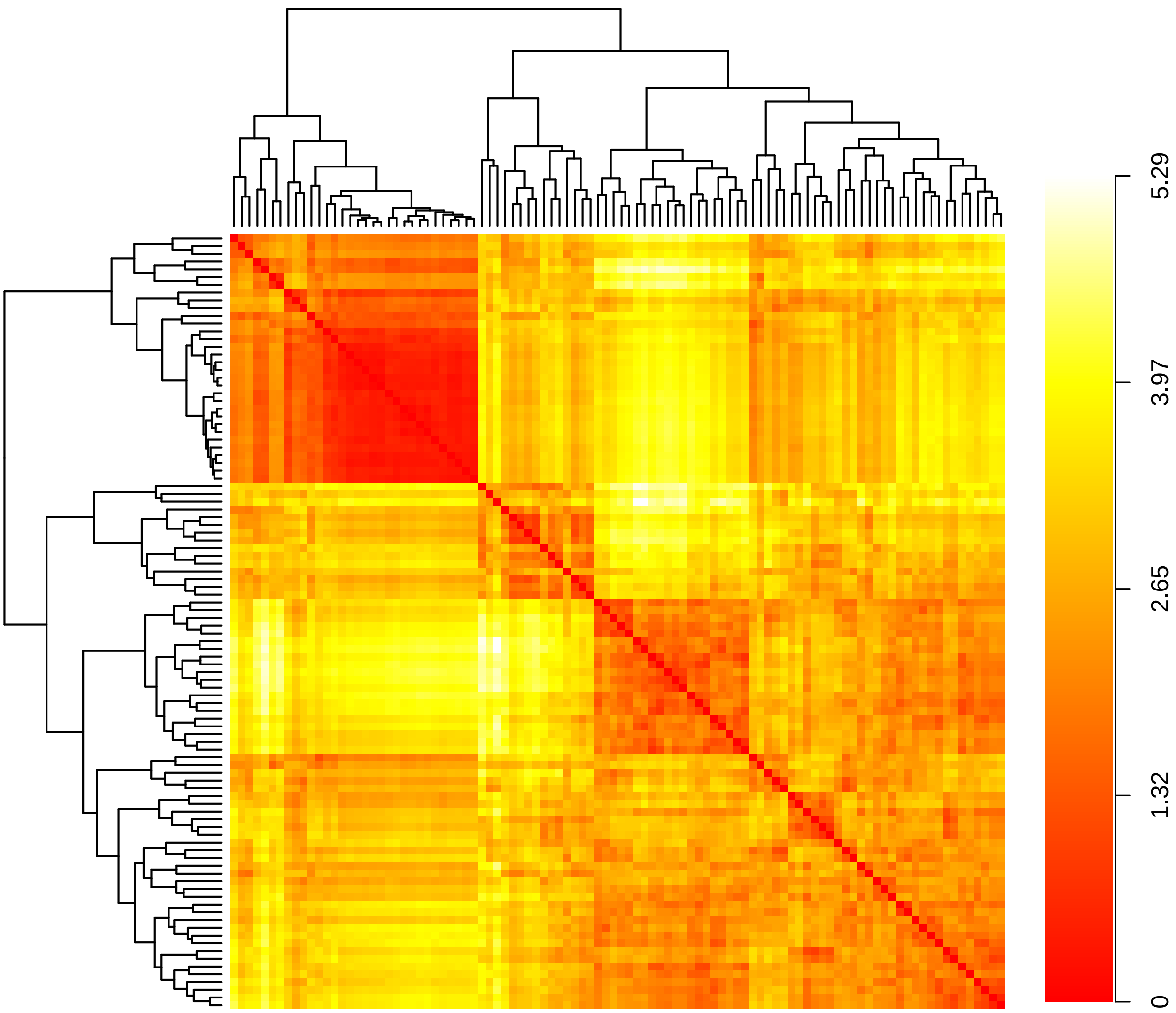

Figure 80: Pairwise RMSD in Cluster 27 (random sample of 100 fragments).

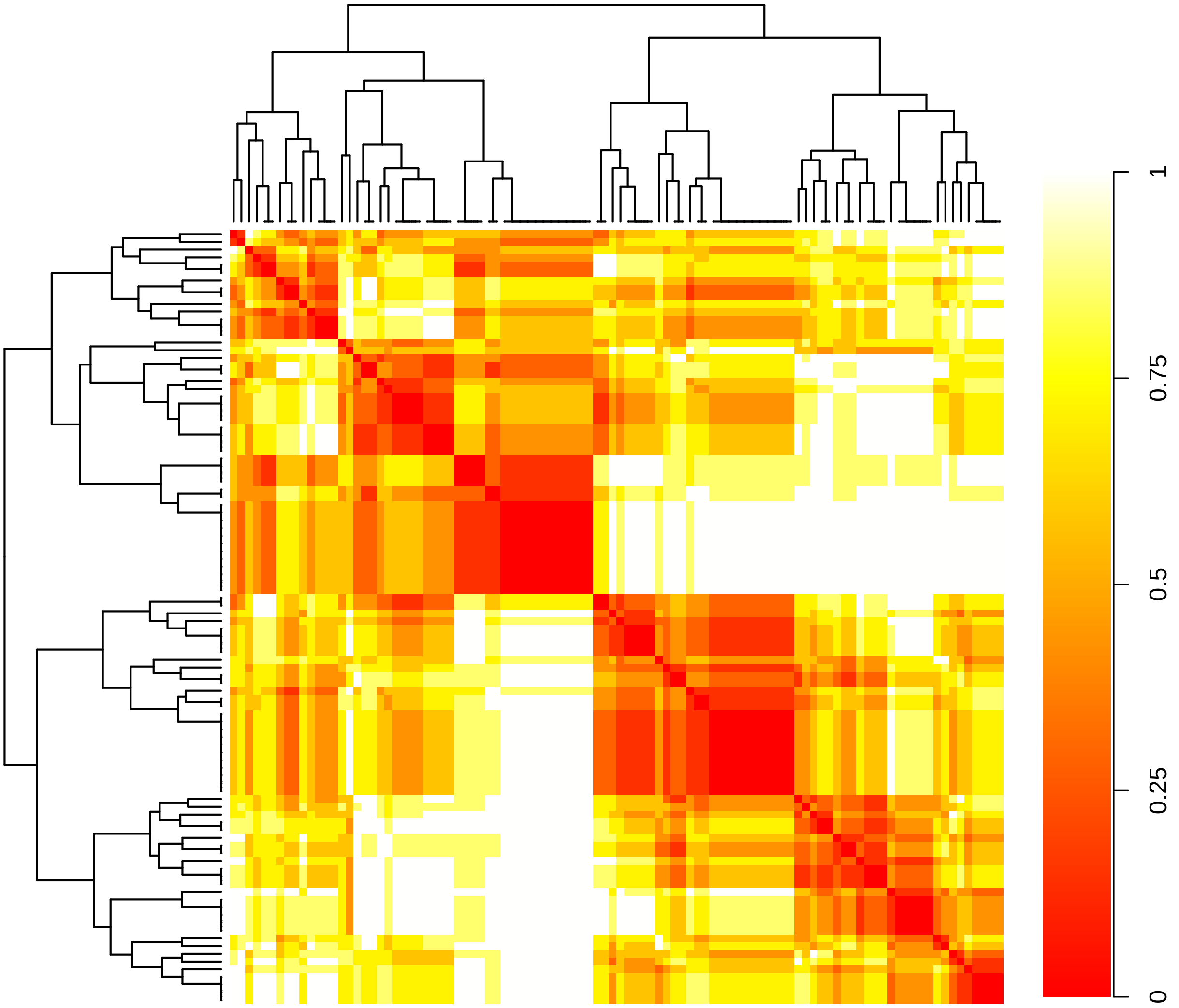

Figure 81: Pairwise secondary structure dissimilarity (1-Q3) in Cluster 27 (random sample of 100 fragments).
